# Impact of the Quality of Bowel Cleansing on the Efficacy of Colonic Cancer Screening: A Prospective, Randomized, Blinded Study

**DOI:** 10.1371/journal.pone.0126067

**Published:** 2015-05-07

**Authors:** Jürgen Pohl, Marc Halphen, Hans Rudolf Kloess, Wolfgang Fischbach

**Affiliations:** 1 Klinikum Friedrichshain, Berlin, Germany; 2 Norgine Limited, Harefield, United Kingdom; 3 Norgine GmbH, Marburg, Germany; 4 Klinikum Aschaffenburg, Aschaffenburg, Germany; University Hospital Llandough, UNITED KINGDOM

## Abstract

**Objectives:**

Efficacy of two low volume bowel cleansing preparations, polyethylene glycol plus ascorbate (PEG+Asc) and sodium picosulfate/magnesium citrate (NaPic/MgCit), were compared for polyp and adenoma detection rate (PDR and ADR) and overall cleansing ability. Primary endpoint was PDR (the number of patients with ≥1 polypoid or flat lesion recorded by the colonoscopist).

**Methods:**

Diagnostic, surveillance or screening colonoscopy patients were enrolled into this investigator-blinded, multi-center Phase IV study and randomized 1:1 to receive PEG+Asc (administered the evening before and the morning of colonoscopy, per label) or NaPic/MgCit (administered in the morning and afternoon the day before colonoscopy, per label). The blinded colonoscopist documented any lesion and assessed cleansing quality (Harefield Cleansing Scale).

**Results:**

Of 394 patients who completed the study, 393 (PEG+Asc, N=200; NaPic/MgCit, N=193) had a colonoscopy. Overall PDR for PEG+Asc versus NaPic/MgCit was 51.5% versus 44.0%, p=0.139. PDR and ADR on the right side of the bowel were significantly higher with PEG+Asc versus NaPic/MgCit (PDR: 56[28.0%] versus 32[16.6%], p=0.007; ADR: 42[21.0%] versus 23[11.9%], p=0.015), as was detection of flat lesions (43[21.5%] versus 25[13.0%], p=0.025). Cleansing quality was better with PEG+Asc than NaPic/MgCit (98.5% versus 57.5% considered successful cleansing). Overall, there were 132 treatment-emergent adverse events (93 versus 39 for PEG+Asc and NaPic/MgCit, respectively). These were mainly mild abdominal symptoms, all of which were reported for higher proportions of patients in the PEG+Asc than NaPic/MgCit group. Twice as many patients in the NaPic/MgCit versus the PEG+Asc group reported tolerance of cleansing solution as ‘very good’.

**Conclusions:**

Compared with NaPic/MgCit, PEG+Asc may be more efficacious for overall cleansing ability, and subsequent detection of right-sided and flat lesions. This is likely attributable to the different administration schedules of the two bowel cleansing preparations, which may positively impact the detection and prevention of colorectal cancer, thereby improving mortality rates.

**Trial Registration:**

ClinicalTrials.gov NCT01689792.

## Introduction

Colorectal cancer (CRC) is the third most common cancer worldwide, estimated to cause 9% to 12% of all cancer deaths in the United States, Canada and Europe [1–5], and accounts for every seventh case of cancer in Germany [6]. Screening has led to a reduction in the number of deaths caused by CRC [7]. Although colonoscopy is currently considered to be the gold standard for detecting and removing colonic adenomas and neoplasms [8–10], Rex *et al*. reported that 27% of adenomas ≤5 mm in size, 13% of adenomas 6–9 mm in size and 6% of adenomas ≥1 cm were missed by experienced colonoscopists [10].

Suboptimal colonoscopy performance can result from a number of factors, including ineffective bowel cleansing prior to colonoscopy [11]. Inadequate bowel cleansing occurs in about one in five patients [11,12], with the right side of the bowel most affected [9], and some data indicate that it is independently associated with increased risk of both polyps and adenomas being missed during the colonoscopy procedure [13]. Froehlich *et al*. reported that the rate of detection of polyps less than 9 mm in size was statistically significantly higher in patients with well-prepared compared to poorly-prepared bowels (21.8% versus 19.0%, p <0.0001), whereas detection of polyps greater than 9 mm or colon cancer was less dependent on cleansing quality [12]. In particular, suboptimal bowel cleansing is most likely to affect lesion detection in the right (proximal) side of the colon [14]. Many reports have shown that protection from distal colon cancer is easier to achieve than for proximal colon cancers, for which colonoscopy adds little or less protective benefit [14–18]. Effective proximal colon cleansing is also particularly important for the detection of the endoscopically subtle serrated, flat or depressed lesions, which are preferentially distributed in this part of the colon [14]. From a patient's perspective, bowel preparation has been identified as one of the most burdensome elements of colon screening [19].

Commonly-used bowel preparation regimens include low and high volume polyethylene glycol (PEG), an osmotic laxative, with and without additional purgatives, and the stimulant sodium picosulfate with the hyperosmotic magnesium citrate (NaPic/MgCit) [20]. A recent meta-analysis showed statistically significant improvements in segmental cleansing using PEG over sodium phosphate (NaP) in some dosage regimens, such as previous day dosing, and in studies focusing on cleansing of the proximal bowel [20].

To our knowledge, this is the first prospectively designed study to compare detection rates of colonic lesions following bowel cleansing with two different low volume preparations: the osmotically-acting PEG plus ascorbate (PEG+Asc; MOVIPREP) or the stimulant and hyperosmotic NaPic/MgCit (CitraFleet). The primary endpoint of this study is polyp detection rate (PDR), with adenoma detection rate (ADR) as the key secondary endpoint. It was expected that PEG+Asc would result in better bowel cleansing than NaPic/MgCit and thus higher PDR. Additionally, comparisons are made between treatments for PDR and ADR in the right versus the left side of the colon, detection of cancer, flat lesions and advanced risk lesions, colonoscopy completion rates, cleansing quality, and acceptability and tolerability. The primary endpoint was not met since the study was terminated following the planned interim analysis.

## Methods

### Study Design

The protocol for this trial and supporting CONSORT checklist are available as supporting information; see S1 CONSORT Checklist, S1 Protocol and S1 Protocol Amendment. The study was designed as a randomized, investigator (colonoscopist)-blinded, multi-center Phase IV interventional study to assess the efficacy, acceptability and tolerability of PEG+Asc versus NaPic/MgCit (both oral administration) for bowel cleansing prior to complete colonoscopy. The prospectively specified primary endpoint for this study was the PDR, defined as the number of patients with at least one polyp or flat lesion as recorded by the gastroenterologist performing the colonoscopy in relation to the total analysis population. The key secondary endpoint was ADR, defined as the number of patients with at least one adenoma, as confirmed by the pathologist in relation to the total analysis population. PDR and ADR by location (left- and right-sided detection rates, where the left side includes the rectum, sigmoid colon, descending colon and left half of the transverse colon, and the right side includes the right half of the transverse colon, the ascending colon, and the cecum) was also investigated. Other secondary endpoints were the cancer detection rate, flat lesion detection rate, advanced risk lesion detection rate (lesions >1 cm, with high-grade dysplasia and/or villous architecture), colonoscopy completion rate, colon cleansing quality (according to the Harefield Cleansing Scale [21]), and the acceptability and tolerability of the study medication. Classification of flat lesions was performed by the gastroenterologist according to the Paris classification of colon polyps [22]. All adenomas and flat lesions were sent to the pathologist and a histological diagnosis was confirmed, differentiating flat lesions (adenomatous or non-adenomatous), advanced-risk flat lesions and cancers.

Clinical trial registration number: NCT01689792 (http://ClinicalTrials.gov). This study was submitted to ClinicalTrials.gov for registration prior to study commencement, however due to an administrative delay, it was made publicly available on the registry after patient enrolment had begun. The authors confirm that all ongoing and related trials for this drug/intervention are registered.

### Ethics Statement

The study received Independent Ethics Committee approval on 13 September 2011 from Ethik-Kommission bei der Landesarztekammer Hessen, Germany.

### Study Population

A minimum of 400 patients (maximum 800 patients) aged between 40 and 80 years, with an indication for complete colonoscopy for diagnostic, surveillance or screening purposes (patients with a known personal or familial risk of colon neoplasia or aged between 55 and 80 and willing to undergo screening colonoscopy) were to be enrolled. Patients were recruited and written informed consent obtained by investigators at 17 specialized gastroenterology centers in Germany. All study centers regularly performed colonoscopies in ambulatory patients within the German healthcare system. Patients attended only two study visits, the screening visit and the colonoscopy visit (to be performed within 30 days of the screening visit).

Patients were excluded if they had a history of gastric emptying disorders, ileus, toxic megacolon, gastrointestinal obstruction, colonic perforation, colonic resection or a history of severe renal insufficiency (creatinine clearance <30 mL/min). Other exclusion criteria included known phenylketonuria, glucose-6-phosphate dehydrogenase deficiency, presence of congestive heart failure, acute life threatening cardiovascular disease, or a requirement for permanent medication (such as anti-epileptics) with associated stable serum concentrations.

The initial sample size calculation was based on PDR, assuming rates of 44% for PEG+Asc and 30% for NaPic/MgCit [23,24] and a difference between preparations assumed based on data from previous studies [25,26]. Based on a predicted 7% drop-out rate, a study that enrolled approximately 200 patients in each treatment group would have 80% power to detect the difference between a Group 1 proportion, p_A_, of 0.440 and a Group 2 proportion, p_B_, of 0.300 (odds ratio of 0.545) using a two-group Chi-square test with a two-sided significance level of 0.05. On this basis, a planned interim analysis was conducted once PDR data were available for 400 patients to allow adjustment of sample size (up to 800 patients) if required, or termination based on success or futility. The interim analysis and the re-calculation of sample size were based on an adaptive design as described by Bauer and Köhne [27] and were performed by the study statistician prior to any unblinding. Following this interim analysis, the required sample size for the study was calculated to require a further 1406 patients, considerably more than the stated maximum of 800 patients and the study was therefore terminated. Endpoints were assessed from the data available for the 394 patients (the intention to treat [ITT] population) who were analysed before the interim analysis.

### Study Treatment

Patients were randomized 1:1 to receive a single treatment with either PEG+Asc or NaPic/MgCit prior to colonoscopy. Eligible patients were consecutively allocated a randomization number according to a list generated centrally by the Contract Research Organization statistician (Pierrel Research, Europe GmbH, Germany). Randomization was performed in blocks of four, and study medication packages were distributed sequentially by the Investigator. This mode of patient selection and randomization was expected to minimize imbalance in risk factors and generate a homogeneous study population. Overall, 400 patients were screened from 17 study centers in Germany between 11 November 2011 and 28 November 2012. The last follow-up visit was on 28 January 2013.

Patients randomized to PEG+Asc received a total of 2 L study drug, in two parts: 1 L taken over 1 to 1.5 hours in the afternoon/evening prior to colonoscopy, after a fiber-restricted diet for the day of the first dose, and 1 L taken in 1 hour on the day of colonoscopy, as per the product label. Both doses were to be taken with at least 500 mL additional clear liquid. No solid food was permitted from the time of the first dose of study medication until after the patient had undergone colonoscopy. Colonoscopy was performed at least 1 hour (preferably 2 hours) after the end of PEG+Asc intake.

Patients randomized to NaPic/MgCit received one sachet of study medication dissolved in 150 mL cold water in the morning and another in the afternoon on the day before colonoscopy as per the product label. Each sachet was to be followed by consumption of 250 mL clear liquid per hour for the duration of the intestinal purging. A fiber-restricted diet was followed on the day prior to colonoscopy with no further solid food permitted after a light lunch taken at 12 pm. Only clear liquids were then to be taken before colonoscopy.

Due to the differences between administration schedules and product packaging, neither the patients nor the investigator responsible for dispensing study medication were blinded to study treatment. Colonoscopy, to be conducted and finished by 2 pm, was performed by an experienced gastroenterologist who was blinded to study medication and independent of the investigator who dispensed the medication. The gastroenterologist responsible for the colonoscopy documented the presence of polyps, flat lesions and carcinomas, and assessed the quality of colon cleansing during scope withdrawal using the Harefield Cleansing Scale [21]. Adenomas were subsequently identified by a pathologist. Following colonoscopy, patients received standard care before they were discharged from the study site. Prior to discharge, the investigator conducted a clinical assessment to ensure complete documentation of all side effects related to gut preparation and colonoscopy. Adverse event (AE) data were collected for up to 30 days after colonoscopy. Tolerance, taste and acceptability of study treatment were assessed by the patient using a questionnaire.

### Statistical Analyses

Statistical software analysis was performed using SAS, release 9.2 (SAS Institute Inc., Cary, NC, USA).

The numbers of patients with at least one polyp, adenoma, carcinoma, flat lesion or advanced high risk lesion were each determined and calculated as a percentage of the total analysis population. Data for PDR and ADR were compared between treatments using two-sided Chi-square tests with an α-level of 5%. Additionally, the 95% confidence intervals were determined for detection rates in each of the two treatment groups, and for the difference in rates between treatment groups (data not shown). Descriptive statistics were generated for all continuous efficacy and safety variables.

## Results

### Patient Demographics and Baseline Characteristics

**Fig 1:**
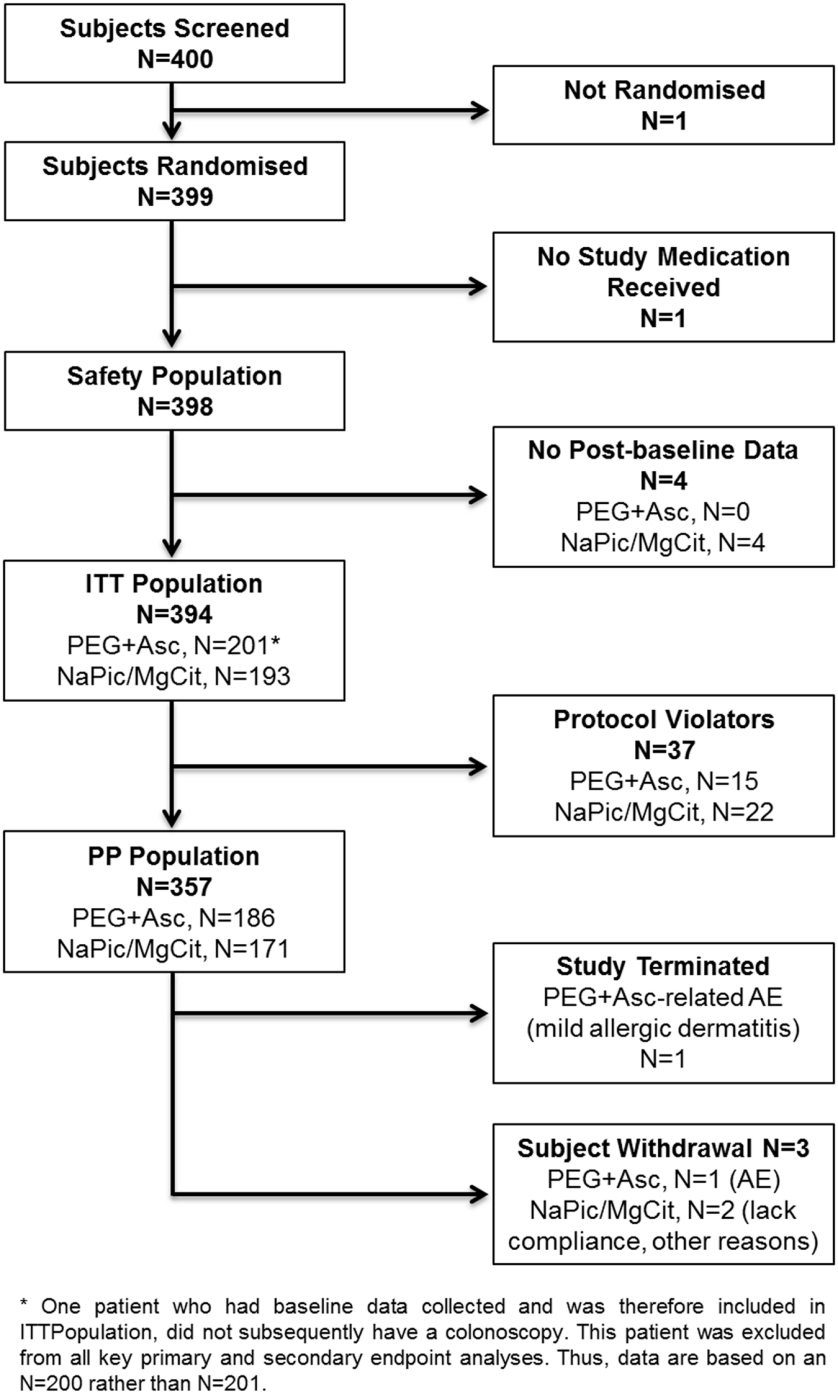
CONSORT diagram showing patient disposition.

Overall, 400 patients were screened from 17 study centers in Germany between 11 November 2011 and 28 November 2012. The numbers of patients included in the safety, ITT and per protocol (PP) populations are presented in Fig 1 (CONSORT diagram) and Table 1. All randomized patients were of European descent and baseline characteristics were generally comparable between treatment groups (Table 1). Importantly, there were no major differences between treatment arms in concomitant diseases (S1 Table) and concomitant medications (including opiates). The last follow-up visit was on 28 January 2013.

**Table 1:** Summary of Patient Demographics and Baseline Characteristics.

		PEG+Asc	NaPic/MgCit	Overall
Screened				400
Randomized				399^[a]^
Safety population		201	197^[b]^	398
ITT population		201^[c]^	193	394
Protocol Violation		15	22	37
PP population		186	171	357
Withdrawals^[d]^/reason		1(adverse event)	2(lack of compliance; other reasons)	3
Gender, n (%)^[e]^	Male	114 (56.7)	99 (50.3)	213 (53.5)
	Female	87 (43.3)	98 (49.7)	185 (46.5)
Age [years], mean (SD)^[e]^		59.5 (9.6)	60.1 (8.8)	59.8 (9.2)
BMI [kg/m^2^], mean (SD)^[e]^		27.5 (4.5)	26.5 (4.7)	27.0 (4.6)
Race, n (%)^[e]^	Caucasian	201 (100.0)	197 (100.0)	398 (100.0)
	Other	0	0	0
Obesity		28 (13.9%)	24 (12.2%)	52 (13.1%)

BMI = body mass index; ITT = intention to treat; PP = per protocol SD = standard deviation; N/n = number of patients; NaPic/MgCit = sodium picosulfate/magnesium citrate; PEG+Asc = polyethylene glycol plus ascorbate.

^[a]^ Of the 400 patients screened, one was not randomized as they were not eligible for treatment. Of the 399 randomized patients, one did not receive any study medication so was not included in the Safety, ITT or PP populations.

^[b]^ Post-baseline data were not available for four patients so these patients were not included in the ITT or PP populations.

^[c]^ One patient who had baseline data collected and was therefore included in ITT Population, did not subsequently have a colonoscopy. This patient was excluded from all key primary and secondary endpoint analyses.

^[d]^ During the treatment or follow-up period.

^[e]^ Data are for the safety population.

### Polyp Detection Rate

**Fig 2:**
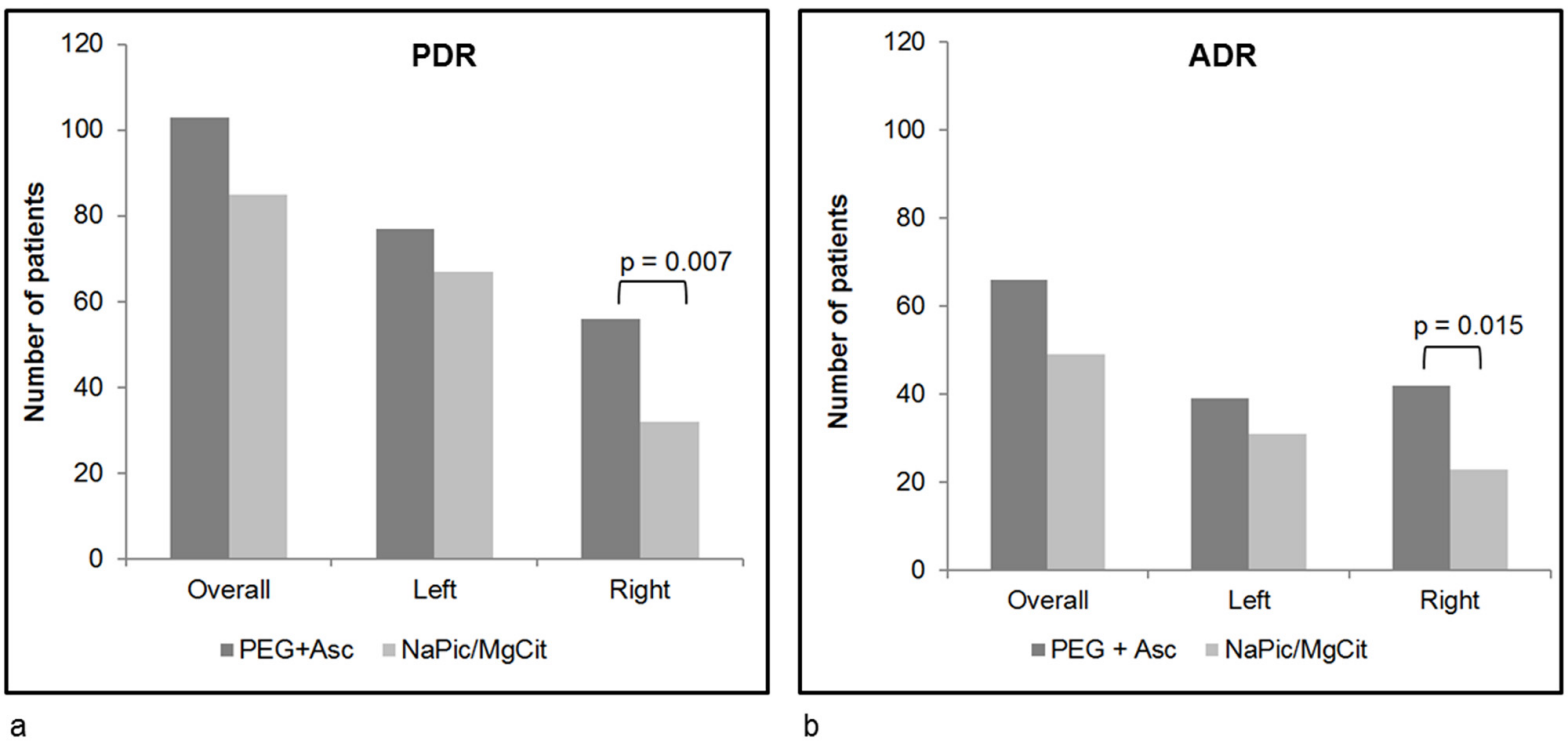
The number of patients with at least one polyp (a) or adenoma (b), overall and on the left and right sides of the colon (ITT population). The left side includes the rectum, sigmoid colon, descending colon, left half of the transverse colon, and the right side includes the right half of the transverse colon, the ascending colon, and cecum. Adenomas were confirmed by a pathologist. Statistical significance was determined by two-sided Chi-square tests. Data are missing for one patient in the PEG+Asc treatment group. ADR, adenoma detection rate; NaPic/MgCit, sodium picosulfate/magnesium citrate; PDR, polyp detection rate; PEG+Asc, polyethylene glycol plus ascorbate.

The primary endpoint of the study was the number of patients with at least one polyp or flat lesion identified during colonoscopy. At the time of the planned interim analysis, 188 (47.7%) patients were identified as having polyps, of which 103 had received PEG+Asc and 85 had received NaPic/MgCit (corresponding to 51.5% and 44.0% of patients in each respective treatment group [p = 0.14]; Fig 2a). As stipulated in the protocol, an interim analysis was performed after data for the primary endpoint became available for 400 patients included in the study; as a consequence the study was terminated since it was calculated that a further 1406 patients would be required to provide a definitive significant difference, a number that was not feasible for the current study and considerably higher than the planned maximum of 800 patients. Analyses of the remaining efficacy variables were therefore performed on the data from the 394 patients who were analysed prior to the interim analysis (the ITT population).

Assessments of PDR for the right and left sides of the colon were additional secondary endpoints of the study. The number of patients identified as having at least one polyp on the left side was slightly higher for those treated with PEG+Asc compared to NaPic/MgCit, although this difference was not statistically significant (77 [38.5%] and 67 [34.7%] patients for PEG+Asc and NaPic/MgCit, respectively, p = 0.44). However, PDR on the right side of the bowel was significantly higher for patients who had received PEG+Asc compared to NaPic/MgCit (56 [28.0%] and 32 [16.6%] patients, respectively, p = 0.007, Fig 2a). Notably, 30 patients treated with PEG+Asc had polyps detected on both sides of the bowel, whereas only 14 patients had polyps identified on both sides in the NaPic/MgCit treatment group.

### Adenoma Detection Rate

For the key secondary endpoint of the study (Table 2), ADR was higher for those who had received PEG+Asc (66 [33.0%]) compared to those who received NaPic/MgCit (49 [25.4%]), but this difference was not statistically significant (p = 0.097; Fig 2b). When ADR was analyzed by location (left- or right-sided adenomas), significantly more adenomas were detected on the right side of the colon when patients received PEG+Asc compared with those who received NaPic/MgCit (42 [21.0%] and 23 [11.9%] patients with at least one right-sided adenoma, respectively, p = 0.015). There was no statistically significant difference concerning the number of patients with left-sided adenomas (19.5% in the PEG+Asc compared to 16.1% in the NaPic/MgCit group, p = 0.373, Fig 2b).

**Table 2:** Summary Table of Key Outcomes.

Parameter Measured	PEG+Asc,Number (%) [CI], N = 200^[a]^	NaPic/MgCit,Number (%) [CI], N = 193	Proportion Difference, % [CI]	p-value
Total PDR	103 (51.5) [44.6, 58.4%]	85 (44.0) [37.0, 51.0%]	7.5 [-2.4, 17.3%]	0.139
PDR on right-side	56 (28.0) [21.8, 34.2]	32 (16.6) [11.3, 21.8]	11.4 [3.3, 19.6%]	0.007
PDR on left-side	77 (38.5) [31.8, 45.2%]	67 (34.7) [28.0, 41.4%]	3.8 [-5.7, 13.3%]	0.436
Total ADR	66 (33.0) [26.5, 39.5%]	49 (25.4) [19.2, 31.5]	7.6 [-1.3, 16.6%]	0.097
ADR on right-side	42 (21.0) [15.4, 26.6%]	23 (11.9) [7.3, 16.5%]	9.1 [1.8, 16.3%]	0.015
ADR on left-side	39 (19.5) [14.0, 25.0%]	31 (16.1) [10.9, 21.2%]	3.4 [-4.1, 11.0%]	0.373
Cancer DR	1 (0.5) [0.1, 2.8%]	0 (0.0%) [0.0, 2.0%]	0.5 [-1.5, 2.8%]	0.325
Advanced-risk lesion DR	11 (5.5) [2.3, 8.7%]	8(4.1) [1.3, 7.0%]	1.4 [-2.9, 5.6%]	0.535
Flat-lesion DR	43 (21.5) [15.8, 27.2%]	25 (13.0) [8.2, 17.7%]	8.5 [1.1, 16.0%]	0.025
Bowel-cleansing success (good/very good) (successful)	98.5%	57.5%		<0.0001

NaPic/MgCit = sodium picosulfate/magnesium citrate; PEG+Asc = polyethylene glycol plus ascorbate; CI = confidence interval; N = number of patients

PDR = polyp detection rate; ADR = adenoma detection rate; DR = detection rate.

^[a]^ = One patient who had baseline data collected and was therefore included in ITT Population, did not subsequently have a colonoscopy. This patient was excluded from all key primary and secondary endpoint analyses. Thus, data are based on an N = 200 rather than N = 201.

### Detection of Specific Lesion Types (Carcinomas, Flat Lesions and Advanced High Risk Lesions)

**Fig 3:**
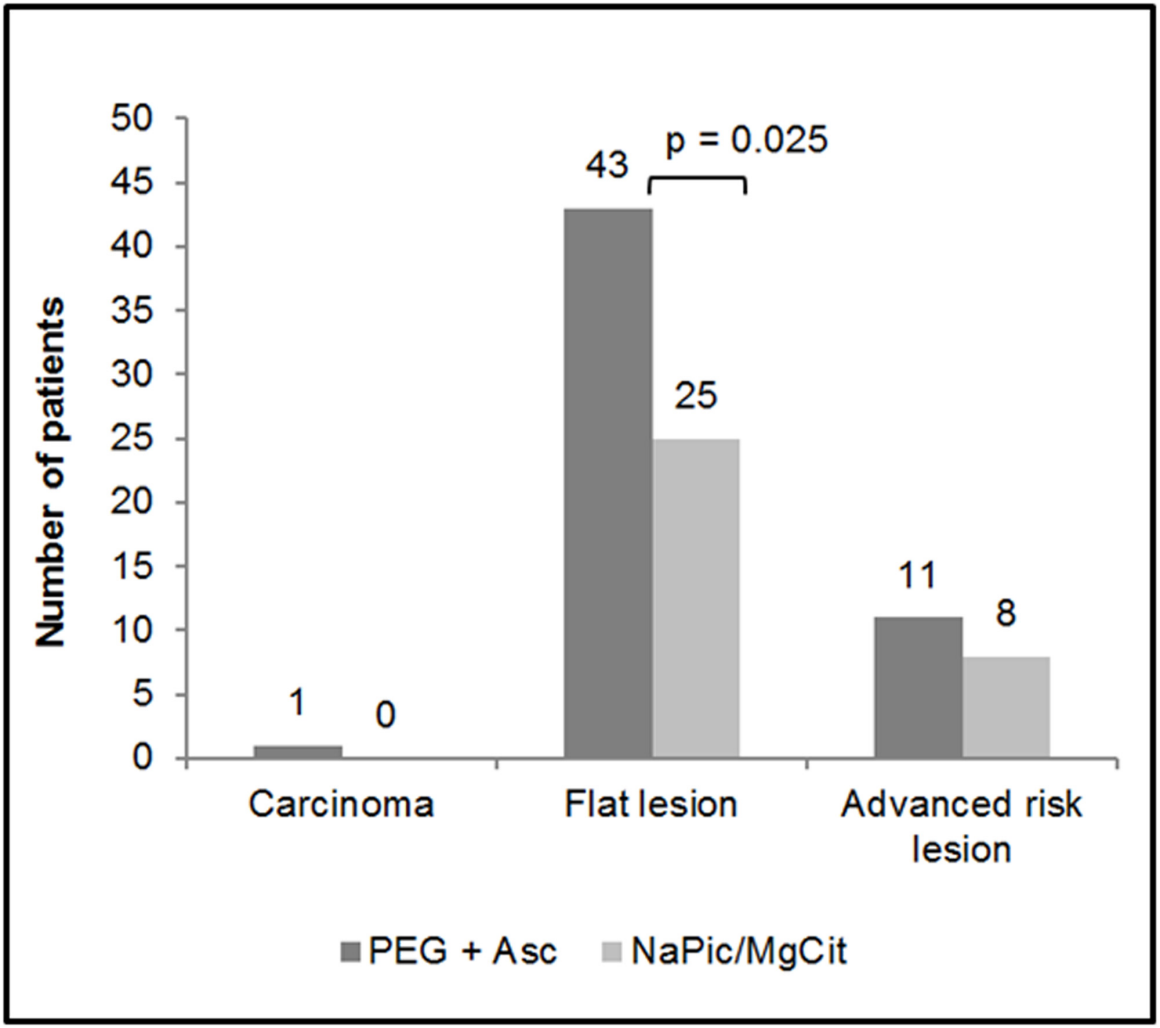
The numbers of patients with at least one carcinoma, flat lesion or advanced risk lesion (ITT population). Statistical significance was determined by two-sided Chi-square tests. Data are missing for one patient in the PEG+Asc treatment group. NaPic/MgCit, sodium picosulfate/magnesium citrate; PEG+Asc, polyethylene glycol plus ascorbate.

There was a trend towards a higher detection rate of carcinomas, flat lesions and advanced risk lesions in patients treated with PEG+Asc compared with NaPic/MgCit, although only the difference in flat lesion detection rates was statistically significant (21.5% versus 13.0% for the PEG+Asc and NaPic/MgCit groups, respectively, p = 0.025, Fig 3). Of the flat lesions detected, 62.8% (27/43) of the PEG+Asc group and 56.0% (14/25) of the NaPic/MgCit group, were adenomatous.

### Colonoscopy Completion Rate

The number of patients who had a completed colonoscopy was comparable between treatment groups with 200 (99.5%) patients completing in the PEG+Asc treatment group compared to 191 (99.0%) patients in the NaPic/MgCit group (p = 0.1489). The mean duration of colonoscopy was also similar in both treatment groups (19.9 minutes for patients who received PEG+Asc and 20.2 minutes for those who received NaPic/MgCit). Colonoscopy was interrupted for only two patients in the study (0.5%), both in the NaPic/MgCit treatment group.

### Colon Cleansing Quality

**Fig 4:**
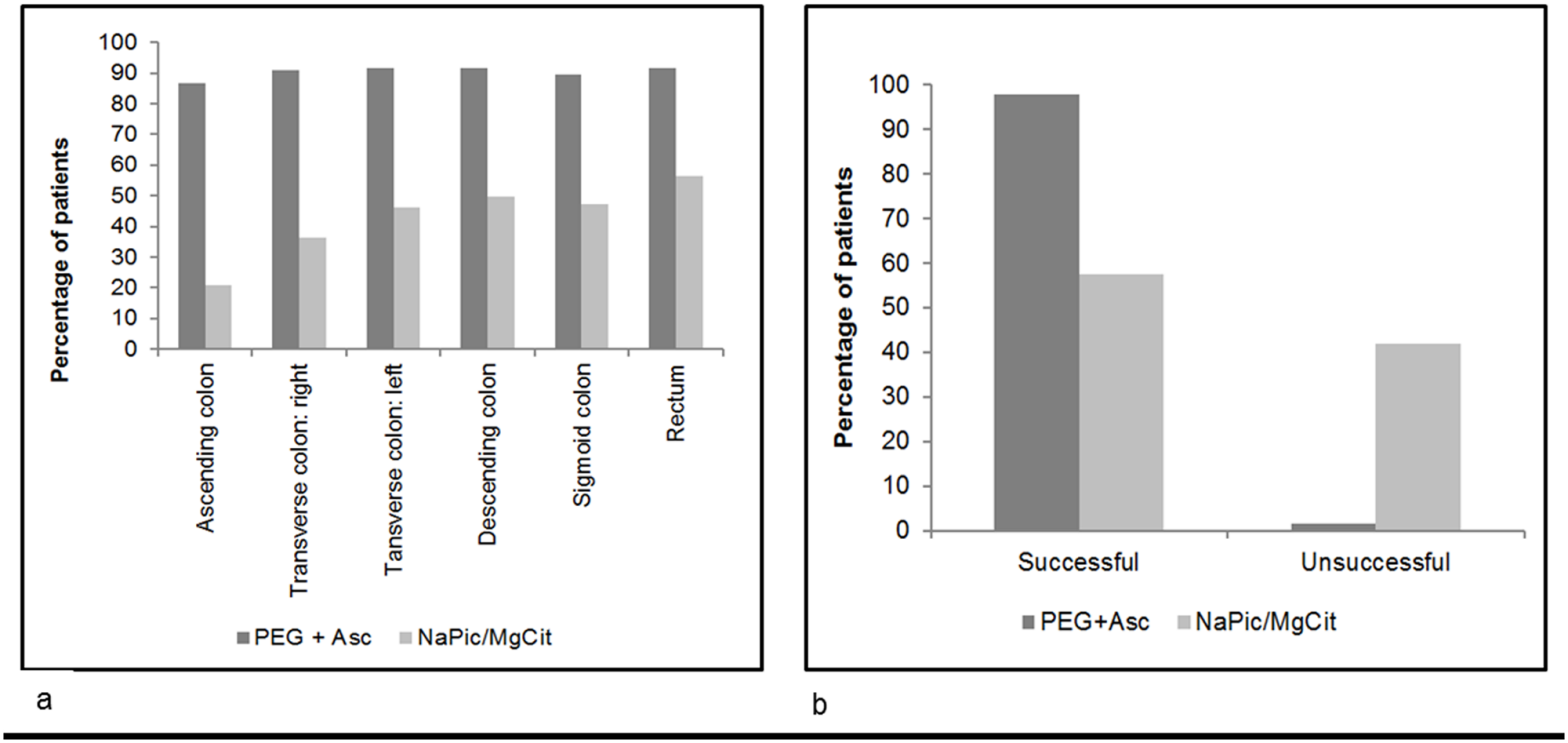
Assessments of cleansing using the Harefield Cleansing Scale [20] (ITT population). (a) The percentage of patients with Grade 4 (Very good) or Grade 3 (Good) cleansing quality in various areas of the colon. Results are presented for those patients who received PEG+Asc and NaPic/MgCit bowel preparations. NaPic/MgCit, sodium picosulfate/magnesium citrate; PEG+Asc, polyethylene glycol plus ascorbate. (b) Overall colon cleansing success of PEG+Asc compared to NaPic/MgCit assessed during withdrawal of the colonoscope.

The cleansing ability of PEG+Asc was better than NaPic/MgCit in all areas of the colon (Fig 4a). The numbers of patients who had cleansing quality of each grade (Grades 0–4) in each bowel section is presented in S2 Table. Similarly, when cleansing quality was assessed overall, PEG+Asc had better cleansing quality compared to NaPic/MgCit. In total, 98.5% of patients in the PEG+Asc group had a Harefield Cleansing Scale classification of A or B (all colon segments with either Very Good or Good cleansing or at least one segment with Moderate cleansing) compared to 57.5% for NaPic/MgCit (Fig 4b). The percentage of patients with an overall cleansing score of C or D (at least one colon segment with Bad or Very Bad cleansing) was higher for patients who received NaPic/MgCit compared to PEG+Asc (42.0% versus 1.5%). The difference in cleansing quality between PEG+Asc and NaPic/MgCit was statistically significant using Fisher’s Exact Test (p<0.0001).

**A** = All colon segments with Grade 3 (Good) or Grade 4 (Very good) cleansing quality,

**B** = At least one colon segment with Grade 2 (Moderate) cleansing quality, other segments with Grade 3 or 4,

**C** = At least one colon segment with Grade 1 (Bad) cleansing quality,

**D** = At least one colon segment with Grade 0 (Very bad) cleansing quality.

NaPic/MgCit, sodium picosulfate/magnesium citrate; PEG+Asc, polyethylene glycol plus ascorbate.

### Safety, Acceptability and Tolerability

Safety data are summarized in Table 3 and S3 Table Overall, more patients in the PEG+Asc treatment group experienced AEs (32.8%) compared with the NaPic/MgCit treatment group (17.3%), and this was statistically significant; however, most events were of mild intensity for both groups.

**Table 3:** Summary of Safety Data.

		PEG+Asc N = 201	NaPic/MgCit N = 197
**Deaths**		None	None
**SAEs**		3 patients	1 patient
		Lower gastrointestinal hemorrhage and post procedural hemorrhage (both mild, not related)	Post procedural complication (moderate, not related)
		Abdominal pain and post procedural complication (both mild, not related)	
		Post procedural hemorrhage (mild, not related)	
Withdrawals		1 patient	None
		Dermatitis allergic (mild, probably related)	
**TEAEs**			
Frequency		93 events in 66 (32.8%) patients	39 events in 34 (17.3%) patients
PTs affecting ≥1% treatment group		Nausea (14.4%)	Nausea (7.6%)
		Abdominal pain (10.9%)	Abdominal pain (4.1%)
		Abdominal pain upper (6.0%)	Abdominal pain upper (1.5%)
		Vomiting (3.5%)	Headache (1.5%)
		Post procedural hemorrhage (1.0%)	Vomiting (1.0%)
			Hemorrhoids (1.0%)
Intensity, n (%)	Mild	83 (89.2)	35 (89.7)
	Moderate	9 (9.7)	4 (10.3)
	Severe	1 (1.1)	0
Causality, n (%)	Probable	55 (59.1)	15 (38.5)
	Possible	29 (31.2)	16 (41.0)
	Unrelated	9 (9.7)	8 (20.5)

N/n = number of patients; NaPic/MgCit = sodium picosulfate/magnesium citrate; PEG+Asc = polyethylene glycol plus ascorbate; SAE = serious adverse event; TEAE = treatment emergent adverse event.

**Fig 5:**
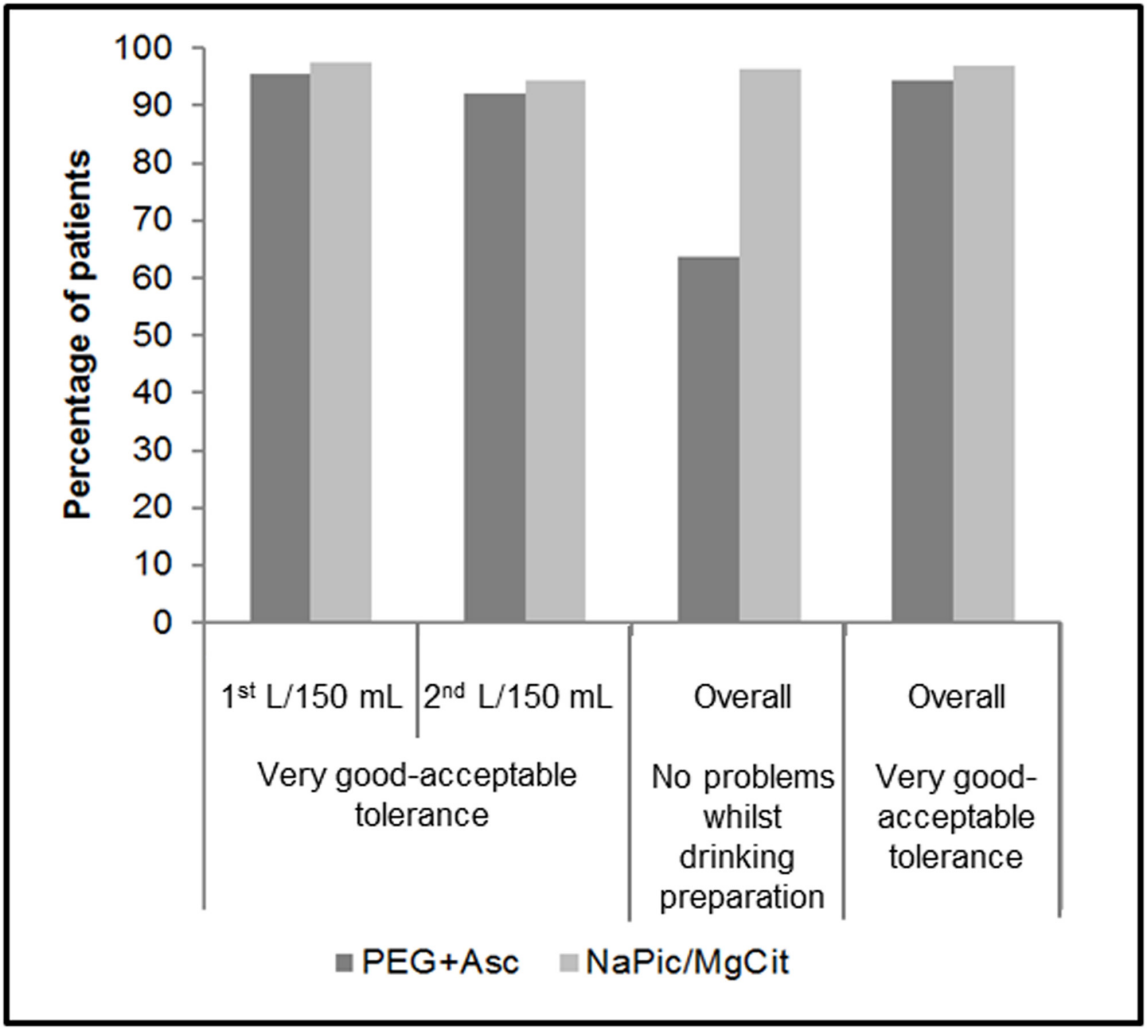
Tolerance of PEG+Asc and NaPic/MgCit assessed by patients via a questionnaire (Safety population). The percentages of patients who reported “Very good”, “Good” or “Acceptable” (Very Good—Acceptable) tolerance are presented for each treatment group together with the percentages of patients who reported that they had no problems while drinking the bowel cleansing solution. NaPic/MgCit, sodium picosulfate/magnesium citrate; PEG+Asc, polyethylene glycol plus ascorbate.

Patient tolerance to PEG+Asc and NaPic/MgCit bowel preparations was assessed by questionnaire. Overall 44.2% of the NaPic/MgCit group experienced a very good tolerance to the cleansing solution compared with 21.9% of the PEG+Asc group. No symptoms were experienced overall while drinking the solutions by 96.4% of the NaPic/MgCit group compared with 63.7% of the PEG+Asc group. (S4 Table). All of these differences between the groups were statistically significant. The majority of patients in both groups rated their overall tolerance of the bowel preparations as very good, good or acceptable (94.5% and 97.0% of patients in the PEG+Asc and NaPic/MgCit groups, respectively; Fig 5). The proportions of patients who reported experiencing symptoms of nausea, vomiting or abdominal pain or discomfort were higher for those in the PEG+Asc treatment group compared to the NaPic/MgCit group following administration of both the first and the second liter of bowel preparation; however, the proportions of patients experiencing any single symptom were low, ranging from 0% to 16.9%. The responses to each of the four questions asked in the questionnaire are summarized in S4 Table.

Patient acceptability of PEG+Asc and NaPic/MgCit bowel preparations was also evaluated by questionnaire (100 mm Visual Analog Scale) and is summarized in Table 4. Patient acceptability and satisfaction were generally high on average, with mean scores of above 70 mm for both assessments and both preparations. A higher proportion of patients in the NaPic/MgCit group reported that consumption of the gut cleansing solution was “Very easy” (69%) compared with the PEG+Asc group (49.3%), although when “Very easy” or “Easy” were considered together, this increased to 97.4% and 93.6%, respectively.

**Table 4:** Acceptability Assessment (Safety Population).

	PEG+Asc, N = 201	NaPic/MgCit, N = 197
**How did you find the product as a bowel cleansing solution? (VAS)** ^[a]^		
Mean [mm] (SD)	71.2 (22.09)	83.8 (17.56)
**Were you satisfied with the whole bowel cleansing preparation? (VAS)** ^[b]^		
Mean [mm] (SD)	75.4 (20.46)	82.8 (18.28)
**Drinking the bowel cleansing solution, as explained in the instructions, was**:		
Very easy, n (%)	99 (49.3)	136 (69.0)
Easy, n (%)	89 (44.3)	56 (28.4)
Quite difficult, n (%)	7 (3.5)	0
Very difficult, n (%)	2 (1.0)	5 (2.5)
Missing, n (%)	4 (2.0)	0

NaPic/MgCit = sodium picosulfate/magnesium citrate; PEG+Asc = polyethylene glycol plus ascorbate; SD = standard deviation; VAS = visual analog scale.

^[a]^ 100 mm VAS ranging from “Totally unacceptable” (0) to “Fully acceptable” (100).

^[b]^ 100 mm VAS ranging from: “Totally dissatisfied” (0) to “Very satisfied” (100).

## Discussion

The data from this study clearly demonstrate increased efficacy of PEG+Asc over NaPic/MgCit in terms of bowel cleansing quality and detection of flat lesions and lesions on the right (proximal) side of the colon. Studies have shown that colonoscopy provides little or less benefit in the prevention of proximal colon cancer [14–18], attributed to inadequate bowel preparation, failed detection of serrated, flat or depressed lesions, inadequate training of gastroenterologists, low cecal intubation rate and low polypectomy rate [14]. Suboptimal bowel cleansing is most likely to affect lesion detection in the right (proximal) side of the colon as mucus and chyme are released from the small intestine after the clearance of stools and these stick to the cecum and right colon [14]. Colon cleansing quality diminishes as the time interval between the end of preparation ingestion and the start of colonoscopy increases and the right side of the colon is particularly affected by this interval [28]. This effect will be most pronounced when there is a long gap between the end of bowel preparation and the start of colonoscopy. Low quality cleansing may result in several lesions being missed during colonoscopy, and interval cancers are particularly prevalent on the right side of the colon [28,29]. The data from the present study suggest that detection of lesions on the right side of the colon may be improved by use of PEG+Asc rather than NaPic/MgCit.

Although bowel preparation regimens are intended to provide perfect visibility of the mucosa, optimal neoplasia detection rate is the clinical aim; thus, cleansing quality scoring can be considered a surrogate marker. Importantly, inadequate colonic preparation has been associated with reduced adenoma detection rates [30]. The superiority of PEG+Asc compared with NaP, where bowel preparation was the primary outcome measure, has previously been reported [31]. The objective of this study was therefore to demonstrate that better cleansing translated into the real-life situation as better polyp detection. On this basis the key endpoints assessed were PDR and ADR, with colon cleansing quality evaluated as a secondary endpoint.

The baseline characteristics of the study population were generally comparable between treatment groups. While men represented a larger proportion of the PEG+Asc group than the NaPic/MgCit group, male gender was not a statistically significant predictor for PDR, ADR, right-sided PDR or right-sided ADR, as confirmed by two different analytical approaches (two univariate logistic regressions and a broader multivariate regression model); data not shown. Therefore, this imbalance was not considered to affect the conclusions of the study.

Flat lesions are predominantly located in the right side of the colon [14] and may be difficult to detect due to their flat morphology and frequency of inadequate bowel preparation in this area [9]. In the present study, the number of flat lesions detected was significantly higher in those patients who received PEG+Asc compared with NaPic/MgCit (p = 0.025), demonstrating the benefits of PEG+Asc over NaPic/MgCit in detecting lesions of this type. Of these flat lesions, a slightly greater proportion in the PEG+Asc group were the adenomatous flat lesion type (62.8% versus 56.0% in the NaPic/MgCit group) than non-adenomatous (hyperplasic) flat lesions. The cleansing ability of the two preparations was assessed using the Harefield Cleansing Scale, a validated method that overcomes some of the drawbacks of other validated scales including the Aronchick, Ottawa and Boston Scales [21]. The Harefield Cleansing Scale assesses cleansing of the whole mucosal surface of the colon using a 5-point ordinal scale (Grades 0–4) which is applied individually to the colon segments, allowing results to be expressed either on a segment-by-segment basis, aggregated into a 4-point overall grade (A-D) or condensed into a binary assessment of cleansing (successful/unsuccessful). The number of patients with Grade 3 (Good) or Grade 4 (Very good) cleansing after receiving PEG+Asc was higher for all areas of the bowel compared to those who received NaPic/MgCit, and the number of patients achieving the best overall cleansing classification (A) was also higher for the PEG+Asc group.

The observed differences between PEG+Asc versus NaPic/MgCit may result from differences in their mechanism of action. NaPic/MgCit consists of a stimulant laxative (NaPic) plus a hyperosmotic laxative (MgCit) [32]. NaPic acts by increasing the frequency and force of peristalsis whereas MgCit increases water content by attracting extracellular fluid efflux through the bowel wall and maintaining oral fluids in the lumen. NaPic requires hydrolysis by colonic bacteria in order to achieve its laxative effect [32,33]. The formulation is locally active and not absorbed in any detectable quantities [32], but due to its composition and mechanism of action, NaPic/MgCit can cause dehydration, electrolyte shift and magnesium retention [32]. Each dose of PEG+Asc combines PEG 3350, a high molecular weight non-absorbable macrogol polymer, with 10.6 g ascorbic acid/sodium ascorbate [32]. Approximately 10% of the ascorbic acid/sodium ascorbate is absorbed in the proximal small bowel, with the rest producing an osmotic effect within the gut, potentiating the purgative effects of PEG [32]. Although the mechanism of PEG is similar to that of osmotic laxatives such as NaP, MgCit and mannitol, it differs slightly in that the electrolyte solution is retained in the colon due to the osmotic effect of the polymer, meaning there is little fluid exchange across the colonic mucosal membrane. Consequently, the potential for systemic electrolyte disturbance is limited [9].

It is also possible that the different administration schedules for the two bowel preparations may have impacted their efficacy. In particular, in agreement with its label, the last dose of NaPic/MgCit was taken the afternoon before colonoscopy, whereas the last dose of PEG+Asc was taken on the morning of colonoscopy. This difference in administration timing potentially introduced a larger time difference between cleansing and colonoscopy for NaPic/MgCit, which has been reported to have a negative effect on the quality of bowel cleansing [34]. Better results in terms of colon cleansing and ADR have been obtained when colon preparation is achieved on the day of colonoscopy [35,36]. Of note, a randomized study of 193 patients using only NaPic/MgCit demonstrated a significant advantage from split-dose with nocturnal pause administration in terms of improved bowel cleansing (particularly proximal colon) [37]. In addition, the split-dose group with nocturnal pause perceived the cleansing process to be easier than the standard administration. However, this current study aimed to assess the effectiveness of the bowel preparations when administered according to the package leaflets of the two preparations. While the mean duration of colonoscopy was similar in both treatment groups, there was no analysis of the time between completion of the bowel preparation and the start of colonoscopy. A comparison of the two treatments administered using a split-dose regimen with nocturnal pause may be warranted in future studies.

Overall, both PEG+Asc and NaPic/MgCit were considered acceptable and tolerable by patients. Although a significantly higher proportion of patients in the NaPic/MgCit group found consumption of the gut cleansing solution “Very easy” compared with the PEG+Asc group. When “Very easy” or “Easy” were considered together, the groups were comparable (93.6% of patients who received PEG+Asc and 97.4% of patients in the NaPic/MgCit group). The observation that NaPic/MgCit was generally rated slightly higher than PEG+Asc in terms of patient acceptability and tolerability is likely to be related to the larger volume of solution patients were required to consume when they took PEG+Asc.

Recently, the 2 L PEG+Asc solution has become more frequently used than the traditional 4 L volume that was the standard for PEG-based bowel-cleansing formulations, as many patients find consuming a high volume of PEG unpleasant or intolerable [38], which may compromise the efficacy of these preparations due to poor patient compliance [34]. The efficacy of PEG+Asc (2 L preparation) has been demonstrated to be non-inferior to standard PEG with electrolytes (Klean-Prep, 4 L preparation), without compromising safety [38], and compared to PEG with electrolytes, PEG+Asc was also rated more acceptable and better tasting by patients [38]. However, the volume of solution that patients are required to consume remains higher for PEG+Asc than NaPic/MgCit, and is likely to account for the higher rating for NaPic/MgCit than PEG+Asc in terms of patient acceptability and tolerability.

Significantly more treatment-emergent AEs were experienced in the PEG+Asc group (32.8%) compared to the NaPic/MgCit group (17.3%). However, nearly all events related to study medication were mild gastrointestinal symptoms, including nausea, abdominal pain and vomiting. In a small number of instances the intensity was moderate. Only one severe event was observed in the PEG+Asc group, which was not related to study treatment. The AEs seen for PEG+Asc were in line with its known safety profile, which has been established in several large clinical studies, and also in clinical practice, and the overall risk/benefit evaluation is considered positive. Overall, the AE profile, acceptability and tolerability of NaPic/MgCit were more positive than PEG+Asc. However, given that tolerability was considered to be at least acceptable by over 90% of patients, the benefits of PEG+Asc in terms of identification of lesions, in particular those on the right side of the bowel, may be considered to outweigh the potential for more side effects and poorer patient acceptability, and could be considered sufficient to warrant its use in preference to NaPic/MgCit.

The study was terminated after the planned interim analysis as it was not statistically possible to accept or reject the null hypothesis that there was no difference between the bowel cleansing preparations in terms of overall PDR, and the subsequent recalculation of the sample size indicated that a number greater than the planned maximum 800 patients would be required. The basis for defining PDR rather than ADR as the primary outcome measure of the study was evidence published by Froehlich *et al*. suggesting that the cleansing quality of the bowel before colonoscopy impacted PDR without impacting detection of colon cancer [12]. The data presented in the current study show that PEG+Asc cleans all areas of the bowel more effectively than NaPic/MgCit when administered according to the relevant product labels, and that rates of detection of flat lesions overall and right-sided polyps and adenomas were significantly higher when PEG+Asc was used to cleanse the bowel before colonoscopy (p-values of 0.025, 0.007 and 0.015, respectively).

Similar to Froelich *et al*, there was no significant difference in carcinoma detection rate in the current study, but in addition, PDR was not significantly affected by bowel cleansing regimen despite the differences between treatments in overall cleansing quality. It should be noted that the lack of significance for carcinoma detection in the current study may reflect the very small rates of carcinoma detection in both treatment groups (0.5% and 0% for PEG+Asc and NaPic/MgCit, respectively).

In conclusion, the results of the current study suggest that PEG+Asc may be more efficacious, both in terms of overall cleansing ability and subsequent right-sided lesion detection, compared to NaPic/MgCit. This may be attributable to the different administration schedules of the two bowel cleansing preparations. As evidence suggests that the quality of bowel cleansing is pertinent to the quality of colonoscopy [12], the improvements in cleansing provided by PEG+Asc may positively impact the detection and prevention of CRC, and thus significantly improve mortality rates from this prevalent disease.

## Supporting Information

S1 CONSORT Checklist CONSORT checklist. (DOC)

S1 Protocol Trial protocol. (PDF)

S1 Protocol Amendment Protocol Amendment. (PDF)

S1 Table (DOCX)

S2 Table (DOCX)

S3 Table (DOCX)

S4 Table (DOCX)
