# Supplementary material for: Impact of the Quality of Bowel Cleansing on the Efficacy of Colonic Cancer Screening: A Prospective, Randomized, Blinded Study
Source: PLoS One. 2015 May 7;10(5):e0126067. doi: 10.1371/journal.pone.0126067 (PMC4423835; doi:10.1371/journal.pone.0126067)
Supplement: S1 Protocol — (PDF) [file pone.0126067.s002.pdf]

NOR-01/2011(PDR)

Protocol

A multi-centre, randomised, investigator-blinded study comparing the polyp detection rate of two different types of bowel preparation: a 2-litre solution (MOVIPREP®) versus a hyperosmotic and stimulant combined low volume bowel preparation (Sodium Picosulfate and Magnesium Citrate)

Version: 2.0

Version Date: 14 October 2011

EUDRACT Number: 2011-002364-25

**Sponsor:**	Norgine Ltd.<br>Norgine House, Widewater Place, Moorhall Road<br>Harefield, Uxbridge, UB9 6NS, United Kingdom
**Representative:**	[redacted]<br>Therapy Area Director<br>Norgine Ltd. Harefield, UK<br>Tel.: + 44 (0)1895 826 783
**Sponsors Medical Representative**	Hans Rudolf Kloess<br>Norgine GmbH, Marburg, Germany<br>Tel.: +49 (0) 642 19 85 222
**CRO:**	Pierrel Research Europe GmbH<br>Zeche Katharina 6, 45307 Essen<br>Tel.: +49 (0) 201 89 90 0
**Coordinating Investigator:**	Prof. Dr. med. Wolfgang Fischbach<br>Klinikum Aschaffenburg, Innere Medizin<br>Am Hasenkopf 1<br>63739 Aschaffenburg<br>Tel.: +49 (0) 6021 32 3011

Confidentiality Statement
The information contained within this document is confidential and is the property of Norgine Ltd. This material should only be used in connection with matters authorised by Norgine Ltd. The information should not be disclosed to others without prior written authorisation of Norgine's representative.

Table of Contents

1 OVERVIEW 6
2 LIST OF ABBREVIATIONS 11
3 SUMMARY 12
4 ADDRESSES AND RESPONSIBILITIES 14
5 INTRODUCTION 19
5.1 Aim of the Study 19
5.2 Rationale 20
5.3 Risk/Benefit Analysis 20
6 STUDY OBJECTIVES 22
7 STUDY DESIGN 23
7.1 Study Centres 24
7.2 Study Duration 24
8 SUBJECT POPULATION 25
8.1 Inclusion Criteria 25
8.2 Exclusion Criteria 25
8.3 Sample Size 26
8.4 Premature Termination of the Study 26
8.5 Subject Withdrawal 27
8.6 Closure of a Study Centre 27
8.7 Patient Replacement 28
9 STUDY MEDICATION 29
9.1 Packaging and Labelling 30
9.2 Accountability of Study Medication 30
9.3 Storage 31
9.4 Recording of Compliance 31
10 TREATMENT 32
10.1 Treatment Allocation 33
10.2 Randomisation and Blinding 34
11 CONCOMITANT MEDICATION 36

12 CONDUCT OF THE STUDY AND METHODS OF ASSESSMENT 37
12.1 Conduct of the Study 37
12.1.1 Visit 1 – screening visit 37
12.1.2 Visit 2 – colonoscopy visit 38
12.2 Follow up assessments 39
12.3 Methods of Assessment 39
12.3.1 Patient documentation form 39
12.3.2 Harefield Cleansing Scale 40
12.3.3 Classification of colon polyps 41
12.3.4 Classification of rectal polyps 41
12.4 Premature Study Termination 41
12.5 Restrictions during the Study 41
12.6 Laboratory Assessments 41
12.7 Blood Sampling 42
12.8 Pregnancy Test 42
13 PARAMETERS AND ENDPOINTS 43
13.1 Parameters Measured and Methods 43
13.2 Primary Endpoints 43
13.3 Secondary Endpoints 43
14 METHODS FOR RECORDING SAFETY 45
14.1 Methods for Recording Safety 45
14.2 Adverse Events, Serious Adverse Events, and Reporting 45
14.2.1 Categorisation of Adverse Events 45
14.2.2 Recording and Follow-up of Adverse Events 47
14.3 Serious Adverse Events 48
14.3.1 Definitions 48
14.3.2 Reporting requirements 49
14.3.3 Mandatory information for reporting an SAE 50
14.3.4 Reporting exemptions 50
14.4 Pregnancy 50
14.5 Deaths 51

14.6 Discontinuation/Withdrawal due to Adverse Events/Serious Adverse Events 51
14.7 Reporting to Competent Authorities/IECs/IRBs/Other Investigators 51
14.8 Safety Monitoring Plan 51
15 STATISTICAL METHODS 52
15.1 Determination of Sample Size 52
15.2 Definition of Study Populations for Analysis 52
15.2.1 Safety population 52
15.2.2 Intention to treat population 53
15.2.3 Per protocol population 53
15.3 Primary Endpoint 53
15.4 Secondary Endpoints 53
15.5 Procedures for Handling of Missing or Spurious Data 53
15.6 Statistical Methods 54
15.6.1 Analyses of demographic data and baseline characteristics 54
15.6.2 Analyses of efficacy variables 54
15.6.3 Safety and tolerability data 54
15.7 Interim Analysis 55
15.8 Subgroup analyses 55
16 ETHICAL CONSIDERATIONS AND INSURANCE 56
16.1 Subject Information and Informed Consent 56
16.2 Ethics Committee(s) 56
16.3 Amendments to the Protocol 56
16.4 Insurance 56
16.5 Legal Aspects 57
17 GENERAL OBLIGATIONS, AGREEMENTS AND ORGANISATION 58
17.1 Investigators Brochure 58
17.2 Data Protection and Confidentiality of the Investigator 58
17.3 Source Data Documentation within the CRF 58
17.4 CRFs and Handling 58
17.5 Monitoring (Quality Control) 59

17.6 Audit (Quality Assurance) and Inspection 60
17.7 Storage of Study Documents and Investigator Site File 60
17.8 Confidentiality 60
17.9 Notification of authorities - BfArM and Other Governmental Institutions ... 60
17.10 Publication 61
18 SCHEDULE OF ASSESSMENT/STUDY FLOWCHART 62
19 References 63
20 APPENDICES 64
20.1 PARIS Classification for Colon Polyps 64
20.2 Labels for Study Medication: 66
20.2.1 Label for MOVIPREP® 66
20.2.2 Label for Sodium Picosulfate and Magnesium Citrate preparation (CitraFleet®) 68
20.3 Template for Patient Documentation Form 70
20.3.1 Patient documentation form for MOVIPREP® 70
20.3.2 Patient documentation form for CitraFleet® 80
20.4 SmPC for MOVIPREP® 90
20.4.1 English Version 90
20.4.2 German Version 98
20.5 SmPC of CitraFleet® 107
20.5.1 English version, translated from German version (see section 20.5.2) 107
20.5.2 German version 114
20.6 Declaration of Helsinki: 122

1 OVERVIEW

Study Number:	NOR-01/2011 (PDR)
Title:	A multi-centre, randomised, investigator-blinded study comparing the polyp detection rate of two different types of bowel preparation: a 2-litre solution (MOVIPREP®) versus a hyperosmotic and stimulant combined low volume bowel preparation (Sodium Picosulfate and Magnesium Citrate)
Study Drug:	**MOVIPREP®** containing 100 g PEG 3350, 7.5 g sodium sulfate with 4.7 g ascorbic acid and 5.9 g ascorbate, electrolytes (2.691 g NaCl, 1.015 g KCl), 0.340 g lemon flavour and sweetener (aspartame-acesulfame).
Active Comparator Drug:	**Hyperosmotic and stimulant combined low volume bowel preparation** (Sodium Picosulfate and Magnesium Citrate)<br><br>**CitraFleet®** Each sachet (15.08 g) contains the following active ingredients: 10.0 mg sodium picosulfate, 3.5 g magnesium oxide, 10.97 g citric acid monohydrate
Phase:	IV
Type:	Randomised, investigator blinded interventional multi-centre study
EudraCT Number:	2011-002364-25
Objectives:	1. To compare the polyp and adenoma detection rate of MOVIPREP® versus an oral Sodium Picosulfate/Magnesium Citrate solution<br><br>2. To assess the correlation between the cleansing quality and the detection rate of the two types of bowel cleansing preparations.
Study Design:	This is a multi-centre, randomised, investigator-blinded interventional study in outpatients and inpatients undergoing a morning colonoscopy. Blinding will be maintained by assignment of an investigator responsible for dispensing the Investigational Medicinal Product (IMP) and assessing Adverse Events (AE's) and an independent and blinded gastroenterologist

	to perform the colonoscopy and efficacy assessments. Gut cleansing will be performed using a course of either 2 litres of MOVIPREP® gut lavage solution with 1 litre (or more) of extra clear liquids, or 2 x 150 mL of oral Sodium Picosulfate/Magnesium Citrate preparation followed by 250 ml clear liquid per hour before a morning colonoscopy.<br><br>Each participating centre will use both types of preparation according to the randomisation list.
Primary Endpoint:	Polyp detection rate (PDR) defined as number of patients with at least one polyp or flat lesion as recorded by the endoscopist.
• Key Secondary Endpoint<br><br>• Additional Secondary Endpoints	• Adenoma detection rate (ADR) defined as number of patients with at least one adenoma as confirmed by the pathologist.<br><br>• ADR and PDR by location:<br>• left-sided (rectum, colon sigmoideum, colon descendens, left half of colon transversum),<br>• right-sided (right half of colon transversum, colon ascendens, caecum).<br><br>• Cancer detection rate, defined as number of patients with at least one malignancy in relation to the total analysis population.<br><br>• Flat lesion only detection rate.<br><br>• Advanced risk lesion detection rate (lesions > 1 cm, low grade and/or villous).<br><br>• Colonoscopy completion rate.<br><br>• Colon cleansing quality, as reported by the gastroenterologist, according to the Harefield Cleansing Scale©.<br><br>• Acceptability and tolerability of the study medication.
Study Duration:	Each patient recruited into the study will be treated once with either MOVIPREP® or an oral Sodium Picosulfate/Magnesium Citrate preparation (CitraFleet®). Patients will undergo gut cleansing prior to colonoscopy starting the day before the procedure, according to the presently recommended instructions for MOVIPREP® or CitraFleet®. Recruited patients will receive study medication at the screening visit and will be given detailed

	intake instructions. The scheduled colonoscopy will be performed within 30 days after the screening visit. The end of treatment assessment will be conducted after completion of the colonoscopy procedure when the subject is ready to leave the endoscopy unit.
Study Population:	It is intended to complete the colonoscopy in a minimum of 400 (200 MOVIPREP® and 200 CitraFleet®) patients; based on the results from an interim analysis a maximum of 800 patients (400 MOVIPREP® and 400 CitraFleet®) may be included.<br><br>Inclusion Criteria:<br><br>1. Patient's written informed consent must be obtained prior to inclusion.<br>2. Male or female outpatients or inpatients aged 40 to 80 years with an indication for complete colonoscopy.<br>3. Willing to undergo a colonoscopy for diagnostic or surveillance purposes<br>4. Patients with a known personal of familial risk of colon neoplasia, willing to undergo a screening colonoscopy<br>5. Willing, able and competent to complete the entire procedure and to comply with study instructions.<br>6. Females of childbearing potential must employ an adequate method of contraception.<br><br>Exclusion Criteria:<br><br>Patients will not be eligible to take part in the study if:<br><br>1. History of gastric emptying disorders.<br>2. History of ileus, toxic megacolon, gastrointestinal obstruction and colonic perforation.<br>3. History of phenylketonuria.<br>4. Known glucose-6-phosphate dehydrogenase deficiency.<br>5. Known hypersensitivity to macrogol 3350, sodium sulphate or ascorbic acid/sodium ascorbate.

	6. History of colonic resection.<br>7. Requirement for permanent medication and associated stable serum concentrations (e.g. neuroleptic drugs).<br>8. Presence of congestive heart failure (NYHA III + IV).<br>9. Acute life-threatening cardiovascular disease.<br>10. Documented history of severe renal insufficiency (creatinine clearance <30 ml/min).<br>11. Other contraindication described in the summary of product characteristics (SmPC) of either preparation.<br>12. Patient has a condition, clinically significant laboratory results, or is in a situation which, in the investigator's opinion, may put the patient at significant risk, may confound the study results, or may interfere significantly.<br>13. Application of any unlicensed medication within the previous 3 months or participation in any other research study in the last 3 months.<br>14. Females who are pregnant, nursing or planning a pregnancy.<br>15. Patients who, in the opinion of the investigator, may not be compliant with the study requirements.<br>16. Previous participation in this clinical study.
Efficacy Parameters:	The primary efficacy parameter will be the total PDR, defined as the number of patients with at least one polyp or flat lesion as recorded by the endoscopist.<br>Secondary efficacy parameters are: ADR defined as the number of patients with at least one adenoma in relation to the total analysis population, number of patients with at least one flat lesion, number of patients with cancers, number of left-sided and right-sided polyps, flat lesions and poly adenomas, advanced high risk lesions, quality of cleansing, acceptability and tolerability per treatment group.
Safety Parameters:	A standardised patient questionnaire will assess all AEs related to the preparation used.
Statistical Analysis:	<u>Sample size and expected differences: (calculations for PDR)</u><br>A difference in PDR of 14% of MOVIPREP against CitraFleet®

	can be assumed as a starting point.<br><br>A PDR of 44% is expected for MOVIPREP® versus 30% for CitraFleet®. This PDR detection is based on studies documented in literature (Piarra Blanco, Lee) and the 14% difference on studies published by Cohen (2010) (39% vs. 20%) and Matro (2010) (37% vs. 26%).<br><br>Type of analysis:<br><br>An interim analysis is to be conducted when data for the primary endpoint (i.e. PDR) are available for the first 400 patients included into the study.<br><br>The interim analysis shall indicate whether the assumptions at the beginning of the study have been adequate. Based on the results of the interim analysis, either premature termination of the study due to success or futility, or continuation of the study and recalculation of the sample size will follow.<br><br>The interim analysis as well as the final analysis will be performed based on an adaptive design as described by Bauer and Köhne (1994).<br><br>Power calculations:<br><br>A two-group Chi-square test with a 0.05 two-sided significance level will have 80% power to detect the difference between a Group 1 proportion, pA, of 0.440 and a Group 2 proportion, pB, of 0.300 (odds ratio of 0.545) when the sample size in each group is 186. Assuming a drop-out rate of 7%, approximately 400 patients will be required with a randomisation rate of 1:1.<br><br>However, these assumptions are based on study populations described in the literature which do not exactly reflect the characteristics of the study population in this study. It can be expected that the difference in the detection rates will be lower due to a lower cleansing capability difference between the two preparations. Therefore, the time point of the interim analysis would be a 'best case' scenario and lead to termination of the study due to early success if the basic assumption for the difference in rates is true. On the other hand, the interim analysis will give the opportunity to increase the sample size within a realistic range or to stop the study due to futility.

2 LIST OF ABBREVIATIONS

ADR	Adenoma Detection Rate
AE	Adverse Event
GCP	Good Clinical Practice
CRF	Case Report Form
CRO	Contract Research Organisation
CSR	Clinical Study Report
ECG	Electrocardiogram
HLMBP	Hyperosmotic and Low Volume Marketed Bowel Preparation
ICH	International Conference of Harmonisation of Technical Requirements for Registration of Pharmaceuticals for Human Use
IMP	Investigational Medicinal Product
ITT	Intention To Treat
PDF	Patient Documentation Form
PDR	Polyp Detection Rate
PEG + E	Polyethylene Glycol plus Electrolyte
PP	Per Protocol
SAE	Serious Adverse Event
SmPC	Summary of Product Characteristics
SUSAR	Suspected Unexpected Serious Adverse Reaction
VAS	Visual Analogue Scale
VRS	Verbal Rating Scale

3 SUMMARY

In recent years, the procedure of colonoscopy has gained a growing importance with respect to diagnosis and prevention of colorectal cancer. It is of particular importance for a successful colonoscopy that thorough cleansing of the gut is achieved prior to the colonoscopy. If adequate gut cleansing is not obtained, the colonoscopy may need to be repeated or existing pathological findings may not be observed. As a result, the efficiency of the colonoscopy as a tumour screening tool is reduced.

The use of iso-osmolar polyethylene glycol plus electrolyte (PEG+E) solutions (e.g. Golytely, Golytely RSS) has been well established within the last two decades as a safe and effective gut cleansing approach and represents an established standard for colon preparation. Norgine has successfully developed MOVIPREP®, a modified low-volume (2-litre) PEG+E-containing gut-lavage solution with the addition of ascorbic acid (10 g ascorbic acid per litre gut lavage solution). The efficacy and safety of this solution is similar to a standard 4-litre PEG+E gut cleansing solution, but with an improved patient acceptance.

The ultimate goal of the colonoscopy is to diagnose, and when possible remove, neoplastic lesions, including adenomas, flat lesions as serrated adenomas, other polyps, and malignancies in the colon and rectum. The quality of the colon cleansing is of crucial importance for a successful screening and diagnosis. Polyp detection rate (PDR) and adenoma detection rate (ADR) are established criteria for colonoscopy quality. They are defined as the number of patients within a population in whom at least one polyp or one adenoma was detected. The PDR, along with ADR overall and by location in the colon, as well as the cancer detection rate will be assessed in this present study. Furthermore, this study is designed to investigate the correlation between ADR/PDR and colon cleansing quality as assessed with the Harefield Cleansing Scale©, a 5-step scale used by the investigator. The study endpoints will be compared between MOVIPREP® and CitraFleet®, a hyperosmotic and stimulant combined low volume marketed bowel preparation containing sodium picosulfate and magnesium citrate as active ingredients.

Additionally, the acceptability and tolerability of both preparations will be documented and analysed using a visual analogue scale (VAS, 100 mm) and 3- or 5-point verbal rating scales (VRS) on the Patient Documentation Form (PDF) prior to colonoscopy. A safety evaluation of the study medication will also be performed.

The present study aims at a comparison of PDR and ADR and a comparison of the correlations between colon cleansing quality and neoplastic lesion detection between MOVIPREP® and CitraFleet®. The differences in flavour, volume, and timing of intake play an important role in the acceptability of the solutions. This must be set in relation to the quality of bowel cleansing, as a higher cleansing quality and PDR/ADR may justify a reduced satisfaction with the solution on the patient side; however, low tolerability and acceptability may hinder sufficient intake of an efficacious preparation.

This is a single country, multi-centre, randomised, investigator-blinded, phase IV study

in patients undergoing colonoscopy to compare the PDR and ADR of MOVIPREP® versus CitraFleet® as well as the correlation between colon cleansing quality and PDR/ADR between the two preparations.

The study will be conducted according to the legal requirements of all applicable German laws and the ICH-GCP-guidelines. Prior to the start of the study, all required Ethics Committee approvals will be obtained.

4 ADDRESSES AND RESPONSIBILITIES

Coordinating Investigator:

Name: Prof. Dr. med. Wolfgang Fischbach

Address: Klinikum Aschaffenburg
Innere Medizin
Am Hasenkopf 1
63739 Aschaffenburg, Germany
Tel.: +49 (0) 6021 32 3011
Fax: +49 (0) 6021 32 2024

E-mail: wolfgang.fischbach@klinikum-aschaffenburg.de

Sponsors Representative:

Name: [redacted]

Address: Norgine Ltd.
Norgine House, Widewater Place, Moorhall Road
Harefield, Uxbridge, UB9 6NS
Tel.: +44 (0) 1895 826 783
Fax: +44 (0) 1895 825 865

E-mail: [redacted]

Sponsor's Medical Representative:

Name: Hans Rudolf Kloess

Address: Norgine GmbH, Im Schwarzenborn 4,
Marburg, 35041
+49 (0) 642 19 85 222

E-mail: hrkloess@norgine.com

If the sponsor's representative cannot be reached, the following dedicated 24-hour emergency number can be used to contact a Norgine study physician for resolution of urgent safety questions +44 (0) 1748 828 787.

Sponsor's Project Manager:

Name:	[redacted]
Address:	Norgine Ltd. Norgine House, Widewater Place, Moorhall Road, Harefield, Uxbridge, UB9 6NS, United Kingdom
Tel.:	+44 (0) 1895 413 688
E-mail:	[redacted]

CRO's Project Manager:

Name:	[redacted]
Address:	Pierrel Research Europe GmbH<br>Zeche Katharina 6<br>45307 Essen, Germany
Tel.:	+49 (0)171 5694-499
E-mail:	[redacted]

CRO for Monitoring:

Address:	Pierrel Research Europe GmbH<br>Zeche Katharina 6<br>45307 Essen, Germany

Data Management:

Name:	[redacted]
Address:	Pierrel Research Europe GmbH<br>Zeche Katharina 6<br>45307 Essen, Germany
Tel.:	+49 (0)201 8990-514
Fax:	+49 (0)201 8990-434
E-mail:	[redacted]

Statistical Analysis:

Name: [redacted]

Address: Pierrel Research Europe GmbH
Zeche Katharina 6
45307 Essen, Germany

Tel.: +49 (0)201 8990-402

Fax: +49 (0)201 8990-434

E-mail: [redacted]

Medical Writing:

Name: [redacted]

Address: Pierrel Research Europe GmbH
Zeche Katharina 6
45307 Essen, Germany

Tel.: +49 (0)201 8990-437

Fax: +49 (0)201 8990-251

E-mail: [redacted]

Authorisation

Authorisation of the protocol and obligations: The undersigned confirm that the protocol, the CRFs and the appendices contain the necessary information and guidelines for the conduct of this study. The study will be performed and recorded according to this protocol and all legal obligations and agreements will be followed as laid out below.

We have read the attached protocol "A multi-centre, randomised, investigator-blinded study comparing the polyp detection rate of two different types of bowel preparation: a 2-litre solution (MOVIPREP®) versus a hyperosmotic and stimulant combined low volume bowel preparation (Sodium Picosulfate and Magnesium Citrate)" and agree to abide by all provisions set forth therein. We agree to comply with the International Conference on Harmonisation Guideline for Good Clinical Practice (ICH-GCP), EU Clinical Trials Directive, national and local regulations and the Declaration of Helsinki.

Date: 07 NOV 2011

[illegible]

[redacted]

Sponsors Representative

(continued on next page)

Authorisation
(continued)

Date: 04.11.11 ______________________

Hans Rudolf Kloess, Sponsors Medical Representative

Date: 4.11.2011 ______________________

Prof. Dr. med. Wolfgang Fischbach, Coordinating Investigator

Date: ______________________ ______________________

Principal Investigator

5 INTRODUCTION

5.1 Aim of the Study

PEG 3350 plus electrolytes (PEG+E) containing solutions have been used worldwide for bowel cleansing prior to any clinical procedures such as colonoscopy or x-ray examination for many years. Their effectiveness and safety has been well established in numerous controlled clinical trials and in daily practice. However, the large amount of liquid to be ingested (4 litres) has constituted a drawback to the use of these solutions. To overcome the problem of the need to drink a high volume of up to 4 litres of this slightly salty solution in a few hours (1 litre per hour), a similar solution (MOVIPREP®) with a requirement for a lower volume intake (2 litres) with similar efficacy has been developed and licensed in a number of EU countries and the US.

The use of a combination of ascorbic acid and sodium ascorbate or bisacodyl in addition to PEG + E has enabled a reduction of the total amount of cleansing solution required to be drunk prior to colonoscopy. PEG + ascorbic components have been shown to deliver better results with respect to colon cleansing and adenoma detection than PEG + bisacodyl [1]. Ascorbic acid is an atoxic compound, even if given in very high doses. It is absorbed by the small bowel mucosa using sodium dependent carriers which proved to be saturable, leading to an inverse relationship between ingested and absorbed dose. Humans can consume high quantities of ascorbic acid orally (up to 100 g/day) without any safety concerns. Adverse reactions after the oral consumption of very high doses of ascorbic acid are mainly represented by diarrhoea which can be attributed to the osmotic effect of the non absorbed fraction of the molecule. In addition, ascorbic acid has the advantage of producing a pleasant taste.

The confirmatory proof of the efficacy and safety of MOVIPREP®, containing high doses of ascorbic acid and ascorbate within a PEG + E-containing gut-lavage solution, has now been established in a number of large clinical studies in patients undergoing diagnostic and/or interventional colonoscopy. These studies have demonstrated that MOVIPREP® is a highly effective and safe gut preparation to use prior to colonoscopy, with an improved patient acceptance/tolerance. Several studies have demonstrated that MOVIPREP® enables gut cleansing in a similar fashion to the standard 4-litre PEG+E solutions with up to 90% of good ratings and no differences in the safety profile, including high risk patients.

With several other bowel cleansing preparations marketed, it is necessary to find reliable criteria to assess the bowel cleansing qualities. The prime goal of any bowel preparation is to allow for the detection and secondary removal of as many adenomas and neoplastic colonic lesions as possible, thus to reduce the risk of colorectal cancer. It has been shown that both PDR and ADR were correlated to the quality of cleansing prior to the colonoscopy [2,3]. Therefore, both PDR and ADR can be considered as reliable objective markers for measuring the efficacy of a bowel preparation. PDR and ADR are defined as the number of patients in whom at least one polyp or one adenoma

was identified [4,5]. However, it cannot be assumed a priori that the bowel cleansing quality assessed by the physician allows conclusions to be drawn on the expected PDR/ADR. It is therefore necessary to investigate not only the PDR/ADR, but also the correlation between these rates and the cleansing quality score. Furthermore, the possibility of different cleansing preparations delivering different PDRs or ADRs with similar cleansing quality assessment and vice versa has to be ruled out, as the assessment of cleansing quality is based on the subjective rating of the responsible physician.

With this randomised, investigator-blinded multi-centre study, the efficacy, tolerability, acceptability, and safety of a single dose of MOVIPREP® are investigated in comparison to an oral Sodium Picosulfate/Magnesium Citrate preparation (CitraFleet®). The study population comprises out- and inpatients with clinical gastrointestinal symptoms or know colonic neoplasia risk factors, who are undergoing colonoscopy for diagnostic purposes or colon cancer screening. Colonoscopies will be performed in the morning, as a previous study [6] has shown that the ADR is significantly higher in the morning than in the afternoon. The study will take place in approximately 20 centres in Germany. Shaukat et al. [7] have shown that polyp detection is independent from the investigator performing the colonoscopy. Therefore, centre-related differences in PDR/ADR are not expected.

5.2 Rationale

The aim of the present study is to evaluate the impact of MOVIPREP® compared to an oral Sodium Picosulfate/Magnesium Citrate preparation (CitraFleet®) on PDR including flat lesions, ADR, and cancer detection rate, as well as acceptability and tolerability. All PEG 3350-based gut-lavage solutions are challenging to consume in the required volumes, particularly the 4-litre preparations, but also 2-litre preparations like MOVIPREP®. An improved colon cleansing quality compared to a hyperosmotic and stimulant low volume solution, leading to a better detection of polyps and precancerous lesions in the colon and rectum may justify some possible inconveniences related to the use of MOVIPREP®. In addition, MOVIPREP® and CitraFleet® are compared directly with respect to tolerability and acceptability. Together, these aspects are intended to lead to a better consideration of overall advantages and disadvantages of either bowel preparation.

A randomised, investigator-blinded design to assess a minimum of 400 and a maximum of 800 patients was chosen to demonstrate performance, tolerability and acceptability of MOVIPREP® versus CitraFleet®.

5.3 Risk/Benefit Analysis

The conventional method for gut cleansing prior to colonoscopy involves the use of a high volume of gut lavage solution (such as 4 litres) containing PEG + E. More recently, a PEG 3350-based preparation with ascorbic acid (MOVIPREP®) has allowed the reduction of the consumed volume to 2 litres without an impact on the positive

safety profile and good efficacy rating.

The efficacy and acceptability of MOVIPREP® in gut cleansing has been demonstrated in several clinical studies. Consequently, MOVIPREP® is currently marketed in a number of European countries as well as the United States of America.

The safety profile of MOVIPREP® has been established in several large clinical studies, but also in clinical practice. The overall risk benefit evaluation for MOVIPREP® is positive.

Oral Sodium Picosulfate/Magnesium Citrate preparations are currently approved for marketing in the United States of America and several European countries. Their efficacy, tolerability, and safety have been investigated in several clinical studies. As with MOVIPREP®, their safety profile has been established in large clinical studies and in clinical practice.

6 STUDY OBJECTIVES

The objectives of this study are:

1. To compare the polyp and adenoma detection rate of MOVIPREP® versus an oral Sodium Picosulfate/Magnesium Citrate solution,
2. To assess the correlation between the cleansing quality and the detection rate of the two types of bowel cleansing preparations.

7 STUDY DESIGN

This is a randomised, investigator-blinded, multi-centre, clinical phase IV study assessing the PDR, ADR, cancer detection rate, colonoscopy completion rate, cleansing quality as well as acceptability and tolerability of MOVIPREP® versus oral Sodium Picosulfate/Magnesium Citrate (CitraFleet®). The study will be performed in out- and inpatients undergoing colonoscopies in specialised gastroenterology units in Germany.

A minimum of 400 patients with relevant clinical symptoms for gastrointestinal disorders or familial or personal risk of colon neoplasia will undergo a complete selective colonoscopy. The patients will be randomised to either MOVIPREP® or CitraFleet® treatment groups with a randomisation ratio of 1:1. An interim analysis will be performed as soon as 400 patients are enrolled and have completed colonoscopy to assess whether a recalculation of the sample size will be necessary. Based on this interim analysis, further patients may be included, resulting in a total maximum of 800 patients, or the study may be terminated. The colonoscopies will be completed before 2 p.m. on the day of the investigation.

Patients meeting the inclusion and exclusion criteria will be asked to participate in this study at a routine screening visit. Patients will be given an Informed Consent Form and will be asked to sign the form after discussing any questions with the investigator. Thereafter the responsible investigator will select and dispense the study medication with sequential numbering ensuring that detailed intake instructions are provided to the patient. Dispensing of study medication will be fully documented. The colonoscopy will be scheduled within 30 days of the screening visit.

The colonoscopy procedure and assessment of efficacy criteria will be performed by a gastroenterologist who must be independent of the investigator responsible for dispensing study medication and performing safety assessments. This gastroenterologist must remain blinded to the study medication the patients received. Therefore, patients, investigator and study personnel with access to the study medication must not reveal the patients' treatment in preparation for colonoscopy.

On the day before the colonoscopy (Day -1), the patient will start the study medication intake and if necessary will follow a standardised diet according to the requirements of the bowel preparation (see section 10.1).

Administration of MOVIPREP®

- Day -1: In the afternoon/evening (up to 11 p.m.): 1 litre MOVIPREP® solution followed by at least 0.5 litres of clear liquid.
- Day 0: (day of colonoscopy) from 5 a.m. onwards: 1 litre MOVIPREP® solution followed by at least 0.5 litres of clear liquid.

- There must be a minimum period of 1 hour, preferably at least 2 hours, between the end of the intake of the second dose of the bowel cleansing solution and the beginning of the colonoscopy.

Each litre will be drunk within 1 to 1.5 hours followed by at least 0.5 litres of any additional clear fluid, which may include water, clear soup, fruit juice without pulp, soft drinks, tea and/or coffee (without milk).

Administration of CitraFleet®

- Day -1 (morning): 150 mL CitraFleet® solution followed by 250 mL clear liquid per hour to avoid dehydration while the washout effect persists,
- Day -1: (afternoon): 150 mL CitraFleet® solution followed by 250 mL clear liquid per hour to avoid dehydration while the washout effect persists,
- The morning dose should be taken at about 7 a.m., the afternoon dose between 2 p.m and 4 p.m in the afternoon,
- There will be no administration of CitraFleet® on the day of colonoscopy.

The gastroenterologist performing the colonoscopy will ensure that the planned colonoscopy is performed according to the time schedule given above.

7.1 Study Centres

This is a randomised, investigator-blinded multi-centre clinical phase IV study testing the adenoma and polyp detection rate after administration of MOVIPREP® versus CitraFleet® for bowel preparation prior to a colonoscopy. The study will be conducted in specialised gastroenterology units performing regularly colonoscopies in ambulatory patients within the German healthcare system.

7.2 Study Duration

Patients will have the planned colonoscopy within 30 days of the screening visit. The date of the colonoscopy will be appointed at the screening visit. Patients will be required to start the gut preparation in the morning (CitraFleet®) or afternoon/evening (MOVIPREP®) of the day before the colonoscopy. All assessments stated in the study flow chart will be documented. The total duration of this study is expected to be approximately one year. The beginning of patient enrolment is expected in September 2011 once IEC approval has been obtained. The recruitment time is planned to be approximately 6 months. The last visit of the last patient enrolled will be considered as the end of the study.

8 SUBJECT POPULATION

A minimum of 400 and a maximum of 800 patients are planned to complete the study in approximately 20 specialised gastroenterology sites in Germany. All subjects must be willing to undergo a complete colonoscopy. The colonoscopy must be completed before 2 p.m. on the day of colonoscopy (Day 0).

8.1 Inclusion Criteria

1. Patient's written informed consent must be obtained prior to inclusion.
2. Male or female outpatients or inpatients aged 40 to 80 years with an indication for complete colonoscopy.
3. Willing to undergo a colonoscopy for diagnostic or surveillance purposes
4. Patients with a known personal of familial risk of colon neoplasia, willing to undergo a screening colonoscopy
5. Willing, able and competent to complete the entire procedure and to comply with study instructions.
6. Females of childbearing potential must employ an adequate method of contraception.

8.2 Exclusion Criteria

1. History of gastric emptying disorders.
2. History of ileus, toxic megacolon, gastrointestinal obstruction and colonic perforation.
3. History of phenylketonuria.
4. Known glucose-6-phosphate dehydrogenase deficiency.
5. Known hypersensitivity to macrogol 3350, sodium sulphate or ascorbic acid/ sodium ascorbate.
6. History of colonic resection.
7. Requirement for permanent medication and associated stable serum concentrations (e.g. neuroleptic drugs).
8. Presence of congestive heart failure (NYHA III + IV).
9. Acute life-threatening cardiovascular disease.
10. Documented history of severe renal insufficiency (creatinine clearance <30 mL/min).
11. Other contraindication described in the summary of product characteristics (SmPC) of either preparation.

12. Patient has a condition, clinically significant laboratory results, or is in a situation which, in the investigator's opinion, may put the patient at significant risk, may confound the study results, or may interfere significantly.
13. Application of any unlicensed medication within the previous 3 months or participation in any other research study in the last 3 months.
14. Females who are pregnant, nursing or planning a pregnancy.
15. Patients who, in the opinion of the investigator, may not be compliant with the study requirements.
16. Previous participation in this clinical study.

Each investigator will be supplied with a Non-Evaluable-Patient-Log. The investigator will record every patient asked to participate in the study and the reasons for not enrolling the patient (e.g. refusal of the patient, exclusion criteria).

8.3 Sample Size

Recruitment has to be sufficient to ensure that at least 400 patients are fully evaluable for the primary endpoint after the intake of study medication.

An interim analysis will be conducted when data for the primary endpoint (i.e. PDR) are available for the first 400 patients enrolled into the study. A difference in PDR of 12.5% of MOVIPREP® against CitraFleet® can be assumed as a starting point.

The interim analysis shall indicate whether the sample size assumptions at the beginning of the study have been adequate. Following this interim analysis, either premature termination of the study due to success or futility, or continuation of the study and recalculation of the sample size are possible. The maximum number of patients to be enrolled will be 800.

Based on the results of the interim analysis the assumptions for the sample size calculation will be checked and the sample size will be recalculated and adapted if necessary.

8.4 Premature Termination of the Study

The study will be terminated prematurely if:

- New toxicological or pharmacological findings, serious AEs or frequent AEs that invalidate the positive benefit-risk assessment,
- AEs occur in such prominence (i.e. severity and frequency) that the proposed schedule can no longer be adhered to,
- Rate of recruitment is inadequate to ascertain a timely and orderly completion of the study within a reasonable time frame,
- The scheduled interim analysis shows that the maximum sample size of

800 patients will not be sufficient for assessment of the primary endpoint,

- Significant protocol deviations occur at a frequency implicating the valid and safe conduct of the study,
- The sponsor decides to discontinue the study.

8.5 Subject Withdrawal

The decision to withdraw a patient from the study prematurely is the responsibility of the patient himself or the investigator. The entire study might be stopped if there are unexpected safety concerns.

If the participation of any patient ceases prematurely, the reasons leading to withdrawal from the study should be described in detail in the additional comments section of the Case Report Form (CRF). Regardless of the reason for withdrawing, a final medical examination should be performed and documented. The CRF must be completed as fully as possible.

Once the study is interrupted, no further participation in the study is possible for a patient. Overall, in case of premature termination of a patient the following information should be provided:

- Date and time of last administration of study medication,
- Date of last contact between investigator and patient,
- Reasons for the premature termination,
- Decision for termination made by:
  - investigator,
  - patient.

8.6 Closure of a Study Centre

The study can be terminated at a particular study centre by decision of the investigator, coordinating investigator or sponsor. Reasons for terminating the study at a study centre can be the non-enrolment of patients or non-compliance with the study protocol.

<u>Criteria for discontinuation of the study at one centre:</u>

1. A study centre can be excluded if major violations from the study protocol are discovered.
2. A drop-out rate of more than 30% of initial recruited patients may result in discontinuation of the study at the centre.
3. Centres which will not recruit sufficient numbers of patients per month may be discontinued from the study. If necessary, other centres will be recruited in order

to ensure a sufficient number of patients in the time allowed for the study.

8.7 Patient Replacement

Patients who withdraw after consenting but before starting the bowel preparation will be replaced to ensure that a minimum of 400 patients with complete datasets are enrolled. The maximum number of patients to be dosed with either MOVIPREP® or CitraFleet® is 800.

9 STUDY MEDICATION

IMP: MOVIPREP®

The ingredients of MOVIPREP® are contained in two separate sachets to be reconstituted in water to obtain 1 litre gut cleansing solution.

Sachet A contains the following active substances:

• Macrogol 3550 (PEG)	100 g
• Sodium sulphate anhydrous	7.5 g
• Sodium chloride	2.691 g
• Potassium chloride	1.015 g

Sachet B contains the following active substances:

• Ascorbic acid	4.7 g
• Sodium ascorbate	5.9 g

Other ingredients: 0.340 g lemon flavour and sweetener (aspartame acesulfame)

Pharmaceutical form: Powder for oral solution (per 1 litre) in two sachets

Route of administration: Oral

Posology: The treatment (one pack) will consist of 2 litres MOVIPREP®. One pack will contain four sachets: 2 x PEG + E ('A') and 2 x Ascorbic Acid/Ascorbate ('B'). Two sachets (1 x 'A' plus 1 x 'B') will be dissolved in water to obtain 1 litre gut cleansing solution. Each litre will be drunk within 1 to 1.5 hours followed by at least 500 mL of any additional clear fluid, which may include water, clear soup, fruit juice without pulp, soft drinks, tea and/or coffee (without milk).

Active comparator: Oral Sodium Picosulfate/Magnesium Citrate (CitraFleet®)

The ingredients of CitraFleet® are contained in a sachet to be reconstituted in water, each to obtain a 150 mL gut cleansing solution.

Each sachet contains the following active substances:

• Sodium picosulfate	10.0 mg

	• Magnesium oxide	3.5 g
	• Citric acid monohydrate	10.97 g
	Other ingredients: potassium hydrogen carbonate, saccharin sodium, lemon flavour.	
Pharmaceutical form:	Powder for oral solution (per 150 mL) in sachet	
Route of administration:	Oral	
Posology:	The treatment (one pack) will consist of 2 x 150 ml CitraFleet®. One pack contains two sachets of CitraFleet®. One sachet will be dissolved in water to obtain 150 mL gut cleansing solution. The first 150 ml will be drunk in the morning (before 8 a.m.) followed by 250 mL of any additional clear fluid per hour, which may include water, clear soup, fruit juice without pulp, soft drinks, tea and/or coffee (without milk).<br><br>Breakfast and lunch will consist of low residue foods (see below, section 10.1). The second 150 mL will be drunk in the afternoon (2 p.m. - 4 p.m.), again followed by 250 mL clear fluid per hour. No solid food will be allowed for supper.	

9.1 Packaging and Labelling

MOVIPREP® will be provided in a carton containing four sachets (two sachets 'A' and two sachets 'B'). CitraFleet® will be provided in a box containing two identical sachets. Each box of the study medication will be packed and labelled according to pertinent legal requirements in Germany by Norgine Ltd (examples of label are attached in the appendices of the protocol).

The original labels of the medication will be replaced to clearly identify the packages as study medication.

The study medication will be supplied via Norgine Ltd. to the study centre where it will be stored at room temperature and dispensed by the investigator.

9.2 Accountability of Study Medication

An appropriate authorised person will distribute one box containing a single dose with either MOVIPREP® or CitraFleet® to each patient. The content of the box needs to be consumed according to the detailed intake instructions prior to the planned colonoscopy. The returned used and unused sachets will be counted by the study nurse and returned to Norgine Ltd. for verification and destruction. The procedure will be

documented. The patients will fully document the intake of each sachet of the study medication in an accountability log.

9.3 Storage

The study medication must only be accessible to the Principal Investigator and his authorised personnel. Sachets containing MOVIPREP® or CitraFleet® will be stored in their original packaging below 25°C in a secure storage area at each site. Reconstituted solution should be stored below 25°C. The solutions may be refrigerated but must be kept covered.

9.4 Recording of Compliance

In order to confirm compliance, the following checks will be performed:

- Recording of the number of sachets and the approximate volume consumed as well as date and time of ingestion in a patient documentation form (PDF), which will be handed out to the patient together with the study medication.
- Drug accountability checks by the monitor at the study site to ensure correct allocation of treatment and to reconcile the number of packs received at site against number of patients included. If any unused sachets are returned by the patient these will be checked by the monitor and returned to Norgine Ltd.

Study medication will be shipped to the study site and the amount and condition of the received drug will be recorded on drug accountability forms. At the end of the study or as directed, all unused medication will be returned to Norgine Ltd. (Hengoed, UK). The drug accountability record must be kept current and should contain all dates and quantities of study medication received at the study site or returned to Norgine Ltd.

In addition, a drug dispensing form must be kept and will contain the following information:

- Patient number (patient identification).
- Date and quantity of study drug dispensed to the patient.
- Date and quantity of study drug returned to the study site.
- Date and quantity of used study drug packs returned to the study site.

These inventories must be available for inspection during each monitoring visit. The study site is responsible for the accountability of all used and unused study medication.

10 TREATMENT

A single treatment with MOVIPREP® comprises one carton containing two sachets ‘A’ and two sachets ‘B’. A shaker is also provided to dissolve the powder in 1L of water. The dosing will consist of 2 litres of MOVIPREP®. Each litre is constituted with one sachet ‘A’ and one sachet ‘B’, which is dissolved in a 1 litre solution using water. The solution should be stirred until the powder has completely dissolved and the MOVIPREP® solution is clear or only slightly hazy. This may take up to 5 minutes. The solution can be cooled in the fridge but must be kept covered. The first litre should be drunk within 1 to 1.5 hours in the afternoon/evening before the colonoscopy followed by at least 500 mL of clear fluid, followed by the second litre of MOVIPREP® in the morning of the colonoscopy plus at least 500 mL of additional clear liquid. There should be a period of at least 1 hour, preferably at least 2 hours, between the last intake of gut lavage solution and start of colonoscopy.

A single treatment with CitraFleet® comprises one carton with two identical sachets. Each sachet is to be dissolved in approximately 150 mL of cold water and stirred for 2-3 min until the powder is dissolved. The solution may become hot during dissolving of the powder; in this case the patient may let it cool until the temperature is sufficient for drinking. The first 150 mL should be drunk in the morning of the day before colonoscopy plus 250 mL of additional clear liquid per hour. The second 150 mL should be drunk in the afternoon of the same day followed by 250 mL of clear liquid per hour.

The colonoscopy should be completed before 2 p.m. If the colon preparation is not sufficient for a complete colonoscopy, it must not be repeated for study-related data collection.

The treatment box containing the study medication will be given to each patient at the screening visit for consumption at home prior to the colonoscopy. The contact details of the study site will be required on the treatment label prior to dispensing the study medication. In addition, patients will be given a participation card containing contact and notification details.

The study medication for the study will be released and supplied via Norgine Ltd., New Road, Tir-Y-Berth, Hengoed, Mid Glamorgan, CF82 8SJ, United Kingdom.

Medication will only be released to the investigators after all regulatory documentation (including approval of the protocol by the Competent Authority and an independent Ethics Committee) is provided.

The application of the study medication will be documented in drug accountability forms. The drug accountability records must be kept and should contain all the dates and quantities of study medication received at the study site or returned to Norgine Ltd.

10.1 Treatment Allocation

<u>MOVIPREP® solution will be used as follows:</u>

Day –1 (day before the day of colonoscopy): in the afternoon/evening (up to 11 p.m.): Oral intake of 1 litre solution within 1 to 1.5 hours (approximately 250 mL every 15 minutes) plus at least 500 mL clear fluid. Additional clear liquid may be consumed as desired.

Day 0 (day of colonoscopy): from 5 a.m. onwards: oral intake of 1 litre solution within 1 hour plus at least 500 mL clear liquid. No other solid food intake is allowed until colonoscopy is completed.

Start of colonoscopy: There must be a break of at least 1 hour, preferably 2 hours, between the end of the intake of the last glass of the MOVIPREP® gut-lavage solution and the beginning of the colonoscopy to allow full effectiveness of the gut-lavage solution.

Diet to be followed:

- Morning: normal breakfast;
- Lunch time: light solid food;
- Supper time (up to 1 hour before the start of the gut preparation): clear soup, yoghurt;
- No further solid food until the end of the colonoscopy;
- Clear liquid is allowed, as desired during the whole gut cleansing period.

<u>CitraFleet® solution will be used as follows:</u>

Day -1 (day before the day of colonoscopy): the contents of the first sachet are to be taken prior to breakfast before 8 a.m., followed by approximately 250 mL of water or another clear fluid per hour. A breakfast with low residue food (see below) can be taken between 8 a.m. and 9 a.m., tea or coffee with milk and sugar may be drunk during mid-morning. A lunch containing low-residue food may be eaten at 12 pm. After this lunch, the patient must not ingest anything but clear liquids. The content of the second sachet is to be taken between 2 p.m. and 4 p.m., followed by 250 mL of clear fluid per hour. Supper can be taken at 7 p.m., but must not consist of solid food, only clear fluid (e.g. clear soup) is allowed. The solution should be used immediately after reconstitution.

After 9 p.m. no further food is to be consumed.

Day 0 (day of colonoscopy): On the day of colonoscopy, only clear fluids may be taken in the morning before the procedure.

Clear fluids include water, black tea or coffee (without milk), soft drinks, cordials (no red or purple juices with the exception of cranberry juice), clear soups without solid bits, and gelatine that is neither red nor purple.

Low residue foods include:

- Fats (use sparingly): butter, margarine;
- Eggs: boiled or poached (not scrambled or fried);
- Cereal: crisped rice cereal or cornflakes (no bran);
- Cheese: cream, cottage or cheese sauce;
- Potatoes (no skin): boiled, creamed, mashed, baked;
- Pasta (white only): plain macaroni, spaghetti, noodles etc.;
- Rice (white only): plain, boiled;
- Meat/Fish: minced or well cooked tender, lean, beef, lamb, ham, veal, pork, fish, shellfish;
- Gravy: using stock cubes (white flour or corn flour may be used to thicken);
- Bread: white bread/toast;
- Sugar/sweetener: white sugar, brown sugar, sweetener;
- Dessert: clear jelly.

10.2 Randomisation and Blinding

This is a randomised, investigator-blinded study. The randomisation ratio will be 1:1 to either MOVIPREP® or CitraFleet® bowel preparation. Randomisation will be performed in blocks of four; the packages of study medication will be distributed sequentially. Once the investigator has confirmed a patient's eligibility, a consecutive randomisation number will be allocated to each patient. This procedure must be precisely adhered to, i.e. numbers should not be exchanged or omitted. The investigator will dispense the study medication only to patients eligible for and randomised in the study. It is prohibited to use the investigational products for any other purpose. The study medication will be self-administered by the patient according to the administration instructions.

Due to different packaging composition, volumes, flavours, and intake schedule for both preparations, patients will not be blinded in this study. The investigator blinding will be ensured by a share of responsibilities between two physicians: the colonoscopy will be performed by a blinded gastroenterologist who will have no access to CRFs, patient questionnaires or other patient data that may permit unblinding, whereas

dispensing and retrieval of study medication, instruction of patients and AE assessment will lie with a second investigator. To allow assessment of compliance, the patients will receive an accountability log to capture intake times and volumes of their bowel preparation, as well as possible protocol violations (e.g. unscheduled intake of solid food).

Sealed code break envelopes that will allow emergency unblinding of the respective treatment for the individual patient will be provided to the study centre (one set), the contract research organisation (CRO) (one set) and the sponsor (one set). The code break envelope for a patient may only be broken by the investigator in case of emergency when knowledge of the study treatment is required in order to institute suitable treatment. In this case, the date, time and reason for opening the code break envelope must be documented and confirmed by the investigator's signature.

11 CONCOMITANT MEDICATION

Any concomitant medication that patients require will be given as usual. The patient will be made aware that medication used during the preparation might be flushed out of the system due to the induced artificial diarrhoea. Exceptions are any other investigational or unapproved drug within the last 90 days, but also any other known products to cause gut cleansing (e.g. Golytely, Golytely RSS, saline laxatives, drastic laxatives, enemas). The regular intake of non-drastic laxatives will be recorded in detail. Patients will be informed that laxatives must not be used on the day of preparation and on the day of colonoscopy.

If the physician is of the opinion that such prohibited concomitant medication is necessary for the patient, it should be administered, but this will lead to exclusion of the patient.

12 CONDUCT OF THE STUDY AND METHODS OF ASSESSMENT

All consecutive patients planned to undergo a selective complete colonoscopy for colorectal cancer screening will be asked to participate in the study (screening visit). A questionnaire will be completed to record the patients that refused to participate and their reasoning, or those that were not compliant with inclusion and exclusion criteria.

12.1 Conduct of the Study

12.1.1 Visit 1 – screening visit

All patients will be given detailed instructions about the gut cleansing procedure and the colonoscopy procedure. In addition, after full oral explanation of the study, the investigator will supply the patient with an information sheet. Should the patient agree to participate in the study, two copies of the informed consent form will be signed, one to be retained with the investigator and the second with the patient. The informed consent will always be obtained in writing and needs to be given prior to the conduct of any study procedures, including the distribution of study medication. Patients will be sequentially assigned a screening number starting with S001 per study site upon commencement of screening. All patients will undergo the following assessments to check suitability against the inclusion/exclusion criteria:

- Demographic data (age, gender, ethnic origin),
- Medical history (and current illnesses),
- Eligibility for diagnostic colonoscopy,
- Eligibility for colon cancer screening (patients with familial or personal risk of colon neoplasia),
- Concomitant medication,
- Physical examination, including vital signs (body weight, height, blood pressure, pulse rate, body temperature),
- Pregnancy test, if applicable.

Patients who are considered eligible for the study will be allocated a randomisation number. MOVIPREP® or CitraFleet® will be supplied to eligible patients according to the randomisation scheme. Detailed intake instructions for the respective medication plus a participation card will be given to each patient. Intake instructions will contain details of the diet and fluid intake requirements. A PDF designed for tolerability and acceptability assessment of the study medication will be distributed to the patients (the full questionnaires for MOVIPREP® and CitraFleet® are included in Appendix 20.4).

After an explanation of the questions in this form, patients will record their tolerance assessment of the gut cleansing preparation (either MOVIPREP® or CitraFleet®), taste evaluation, acceptability assessments and any occurrence of pre-defined symptoms.

Investigators will be provided with detailed instructions on the procedure to be followed in the event of a Serious Adverse Event (SAE) or pregnancy.

After satisfactory completion of the screening visit, the date for colonoscopy will be scheduled (within 30 days of the screening visit).

12.1.2 Visit 2 – colonoscopy visit

On the day prior to colonoscopy, patients will start their bowel preparation. After ingestion of each dose of bowel preparation solution, patients will answer the corresponding questions in the PDF.

In the morning of the colonoscopy day, patients will come to the study site to perform the planned colonoscopy. The following assessments will be performed by the investigator prior to the colonoscopy:

- Review of concomitant medication,
- Review of AEs since the screening visit,
- Assessment of study medication compliance,
- PDF collection and review,
- Body weight, height, blood pressure, pulse rate, and body temperature,
- Drug accountability.

The patient will then undergo a full colonoscopy, to be completed by 2 p.m. The procedure will be conducted by an experienced gastroenterologist independent from the investigator responsible for dispense of study medication. This gastroenterologist will be blinded to the study medication.

The following assessments will be performed by the gastroenterologist responsible for colonoscopy:

- Documentation of polyps, adenomas and carcinomas,
- Colon cleansing quality using the Harefield Cleansing Scale©.

If a complete colonoscopy is not possible due to insufficient colon cleansing, a repeated bowel preparation is not allowed within the scope of this study. If a repetition of the colonoscopy is deemed medically necessary by the investigator, any data documented during such a procedure must not be used for the present study.

Standard care after the colonoscopy will be performed before the patient can leave the study site. Prior to discharge from the study site, the investigator will conduct a clinical

assessment after the colonoscopy to ensure complete documentation of all side effects related to the gut preparation and colonoscopy procedure. Discharge from the colonoscopy unit without any outstanding medical concerns will represent the end of the study for each patient.

12.2 Follow up assessments

Patients will be instructed to contact their physician if additional AEs occur within 30 days of the colonoscopy.

12.3 Methods of Assessment

12.3.1 Patient documentation form

Tolerance assessment

1. Five-point VRS per litre/150 mL intake of MOVIPREP®/CitraFleet®

Patients will rate the tolerance of the MOVIPREP®/CitraFleet® solution intake by answering the question in the PDF: *How did you tolerate the first/second litre/150 mL of the MOVIPREP®/CitraFleet® gut cleansing solution?* Possible answers are "very good", "good", "acceptable", "bad" and "very bad".

2. Three-point VRS to assess the degree of difficulty drinking the gut cleansing solution

After completion of the intake the following question will be answered in the PDF: *Did you experience problems during the drinking of the gut cleansing solution?* Possible answers are "none", "some" and "many".

3. Five-point VRS on overall tolerance rating

After completion of the intake the following question will be answered in the PDF: *The tolerance of the MOVIPREP®/CitraFleet® gut cleansing solution was:* "very good", "good", "okay", "bad" or "very bad.

4. Pre-defined symptoms assessment during the intake

Which of the following symptoms occurred during the intake of the first/second litre/150 mL of MOVIPREP®/CitraFleet®? Answers include "none", "nausea", "vomiting", "abdominal discomfort" and "abdominal pain". The pre-defined symptoms will be assessed by the investigator for clinical significance and documented as adverse events.

Taste evaluation

1. VAS (100 mm) taste evaluation after each litre/150 mL intake of

MOVIPREP®/CitraFleet®

Patients will evaluate the taste after each dose by answering the following question in the PDF: *How did you judge the taste of the gut cleansing solution after the first/second litre/150 mL?* Rating will be provided using a 100 mm VAS taste evaluation after each litre rated from: “very bad” [0] to “very good” [100].

2. Three-point VRS on overall taste evaluation

After completing the dose the following question will be answered in the PDF: *The taste of the gut cleansing solution is:* “good”, “okay” or “bad”.

<u>Acceptability</u>

1. Compliance with the intake instructions using the documentation for the intake of MOVIPREP®/CitraFleet® and additional liquid.

The intake of the MOVIPREP®/CitraFleet®solution and additional liquid will be documented in detail and used for a compliance calculation, as well as being assessed for an effect on the acceptability of MOVIPREP®/CitraFleet®.

2. Four-point VRS to assess the ease of drinking MOVIPREP®/CitraFleet®

Patients will rate the ease of drinking the gut cleansing solution by answering the following question in the PDF: *Drinking the test product, as explained in the instructions, was:* with the possible answers: “very easy”, “easy”, “quite difficult” and “very difficult”.

3. VAS (100 mm) to assess the overall satisfaction with the gut cleansing procedure

Patients will record their satisfaction with the whole gut cleansing preparation using a 100 mm VAS satisfaction rated from: “very satisfied” [0] to “totally dissatisfied” [100].

12.3.2 Harefield Cleansing Scale

The gastroenterologist performing the colonoscopy will score the quality of the bowel cleansing for each of the predefined colon areas according to the Harefield Cleansing Scale© as follows:

- Score 4 (very good): Colon empty and clean
- Score3 (good): Presence of clear liquid in the gut, but easily removed by suction
- Score 2 (moderate): Brown liquid or semisolid remaining amounts of stool, fully removable by suction or displaceable, thus allowing a complete visualisation of the gut mucosa
- Score 1 (bad): Semisolid amounts of stool, only partially removable with a risk of incomplete visualisation of gut mucosa

- Score 0 (very bad): Semisolid or solid amounts of stool, consequently colonoscopy incomplete or needs to be terminated

A final grading will assess the overall quality of the colonoscopy preparation as follows:

A: all colon segments clean (score 3-4),

B: at least one colon segment with remaining small amounts of stool, fully removable or displaceable (score 2) no influence on colonoscopy results,

C: at least one segment with only partially removable stools (score 1) colon cannot be fully inspected or

D: at least one colon segment, which cannot be examined related to the presence of remaining stool (score 0).

This grading (A, B, C or D) will be calculated automatically with regard to the 0-4 grade scale for each segment without the intervention of the colonoscopist (Attachment 4).

12.3.3 Classification of colon polyps

Detection rates of flat lesions, polyps with high risk for malignancy and malignant lesions in the colon and rectum will be analysed separately. Classification of adenomatous lesions will be performed according to the PARIS classification of colon polyps (see Appendix 20.1).

12.3.4 Classification of rectal polyps

Hyperplasic polyps of the rectum will only be included if they meet the following criteria (serrated, >1cm and dysmorpheous).

12.4 Premature Study Termination

The patient's participation may be discontinued at any point during the study at their request, or if the physician considers it is in the patient's best interest, or if the patient is not willing to comply with the requirements of the study protocol.

12.5 Restrictions during the Study

There are no additional restrictions to adhere to during the study.

12.6 Laboratory Assessments

There will be no study-specific laboratory assessments.

12.7 Blood Sampling

There will be no study-specific blood drawings.

12.8 Pregnancy Test

At Visit 1, a urine pregnancy test will be performed in female patients of childbearing potential. Testing kits authorised for marketing in Germany will be used.

13 PARAMETERS AND ENDPOINTS

13.1 Parameters Measured and Methods

The primary endpoint (PDR) will be established on the basis of the endoscopic record of the colonoscopy. The key secondary endpoint (ADR) will be established on the basis of the pathological records of the removed adenomas and biopsies. All the other endpoints, except the acceptability and tolerability of the study medication, will be assessed by the gastroenterologist performing the colonoscopy. These data will be collected using a CRF covering only the efficacy parameters. The safety assessments will be performed and recorded in the CRF by an independent investigator also responsible for dispensing study medication.

Patients will complete a PDF during the intake of the gut cleansing solution. The PDF contains a questionnaire to document the intake of MOVIPREP® or CitraFleet®. In addition, a number of questions addressing tolerance and acceptability will be documented by means of VAS and VRS included in the PDF. The degree of gut cleansing will be measured by the colonoscopist employing the Harefield Cleansing Scale© to determine overall cleansing of the colon (from A to D).

13.2 Primary Endpoints

The primary endpoint of this study is the PDR. It is defined as the number of patients with at least one polyp (including flat lesions) found by colonoscopy in relation to the total analysis population.

13.3 Secondary Endpoints

The key secondary endpoint of this study is:

1. ADR, (including flat lesions) defined as number of patients with at least one adenoma as recorded by the pathologist in relation to the total analysis population.

Additional secondary endpoints are:

2. ADR and PDR by location:
   - left-sided (rectum, colon sigmoideum, colon descendens, left half of colon transversum)
   - right-sided (right half of colon transversum, colon ascendens, caecum)
3. Cancer detection rate, defined as number of patients with at least one malignancy in relation to the total analysis population.
4. Flat lesion only detection rate.
5. Advanced risk lesion detection rate (lesions >1 cm, low grade and/or villous).

6. Colonoscopy completion rate.
7. Colon cleansing quality, as reported by the gastroenterologist, according to the Harefield Cleansing Scale©.
8. Acceptability and tolerability of the study medication (according to the questionnaires included in Appendix 20.3).

14 METHODS FOR RECORDING SAFETY

14.1 Methods for Recording Safety

Patients will receive a questionnaire which includes a number of questions on the tolerability of the study medication. The patients will be asked about certain symptoms (none, nausea, vomiting, abdominal discomfort, abdominal pain) which may occur after ingestion of each dose of the study medication. Another question will cover the tolerability of each dose of the study medication (very good, good, acceptable, bad, very bad). The overall rating of the gut preparation will be assessed with a set of questions covering overall tolerability, satisfaction, and problems during intake. For overall tolerance and satisfaction, a VAS will be used. For all other questions, a VRS will be applied. The PDFs for MOVIPREP® and CitraFleet® are included in Appendix 20.3 of this study protocol.

Patients will be asked about any AEs following the screening visit. Also, the PDF will be reviewed prior to colonoscopy by the investigator to detect any clinically relevant AEs documented in the pre-defined symptom question. Training on reporting serious AEs (SAEs) and pregnancies will be provided to the investigator.

AEs which are serious must be reported by telephone or fax to Norgine Ltd. within 24 hours. The investigator may be asked to provide follow-up information by telephone or letter.

14.2 Adverse Events, Serious Adverse Events, and Reporting

An AE is the development of an undesirable medical condition or the deterioration of a pre-existing medical condition following or during exposure to a pharmaceutical product, whether or not considered causally related to the treatment. An undesirable medical condition can be symptoms (e.g. nausea, chest pain), signs (e.g. tachycardia, enlarged liver) or the abnormal results of an investigation (e.g. laboratory findings, electrocardiogram (ECG)). In clinical studies an AE can include an undesirable medical condition occurring at any time, including run-in or washout periods, even if no study treatment has been administered.

This definition includes events occurring from the time of the patient giving informed consent until the end of the study.

14.2.1 Categorisation of Adverse Events

14.2.1.1 Intensity classification

AEs will be classified as mild, moderate, or severe according to the following criteria:

Mild: symptoms do not alter the patient's normal functioning.

Moderate: symptoms produce some degree of impairment to function, but are not hazardous, uncomfortable or embarrassing to the patient.

Severe: symptoms definitely hazardous to well-being, significant impairment of function or incapacitation.

14.2.1.2 Causality classification

The relationship of an AE to the study medication will be classified according to the following:

Probable: A reaction that: follows a reasonable temporal sequence from administration of the drug, follows a known or expected response pattern to the suspected drug; is confirmed by improvement on stopping or reducing the dosage of the drug or could not be reasonably explained by the known characteristics of the patient's clinical state.

Possible: A reaction that follows a reasonable temporal sequence from administration of the drug and that follows a known or expected response pattern to the suspected drug but that could readily have been produced by a number of other factors.

Unrelated: Any event for which there is sufficient and conclusive information that the event is not related to the study drug.

14.2.1.3 Suspected unexpected serious adverse reactions

Suspected unexpected serious adverse reactions (SUSARs) are serious adverse reactions related to the study medication which are both unexpected and drug-related. All SUSARS will be reported to the EC and health authority as per EU and German laws.

The expectedness of an AE/reaction shall be determined by the sponsor according to the most recent SmPC of MOVIPREP®.

14.2.1.4 Abnormal physical examination findings

Abnormal physical examination findings post dosing will be recorded as AEs if the investigator considers they are clinically significant.

14.2.1.5 Other investigation abnormal findings

Other abnormal test findings will be recorded as AEs if the investigator considers they are clinically significant. They may include but are not limited to ECG changes, factors that require changes in the dosage of study medication or administration schedule, discontinuation of the study medication or factors that require intervention or diagnostic

evaluation to assess the risk to the patient.

14.2.2 Recording and Follow-up of Adverse Events

Detection and surveillance

Each patient will be instructed to report any relevant negative change of his/her medical condition (whether considered an AE or not) spontaneously and without delay; he/she will then be asked for further information with regard to the time of onset, duration and intensity of such events. In addition, the PDF section with the pre-defined symptoms will be reviewed for clinically relevant AEs.

Recording of adverse events

A carefully written record of every single AE shall be kept in the CRF by the investigator. Records of AEs shall include the following:

- Description of the event (investigators' term),
- Date and time of onset,
- Date and time of resolution,
- Intensity: 1: mild, 2: moderate, 3: severe (see above),
- Therapy for the event: 0: none, 1: drug, 2: other, 3: 1+2; if applicable: specify on the concomitant medication CRF page,
- Outcome: 1: recovered, 2: not yet recovered, 3: sequels, 4: fatal, 5: unknown,
- Seriousness including life-threatening: 0: no, 1: fatal, 2: immediately life-threatening, 3: permanently or severely disabling, 4: requires or prolongs hospitalisation, 5: congenital anomaly, 6: medically significant,
- Course: 1: continuous, 2: intermittent, 3: once,
- Relation to investigational medicinal product: 0: unrelated, 1: possibly related, 2: probably related,
- Results of a repeated exposure (rechallenge) if applicable.

All AEs have to be followed until the outcome is determined, and/or the end of the planned period of follow-up (30 days after Visit 2). For the purposes of EOT data collection related to unresolved AEs, the outcome may be recorded as “not yet recovered”.

All SAEs will be followed until clinical recovery is complete or until the event has stabilised. For the purposes of data collection related to unresolved but stabilised

SAEs, the outcome may be recorded as "not yet recovered". Follow-up may continue after end of treatment if necessary. The findings of these follow-up investigations will be communicated to Norgine Ltd.

Hospitalisation

The study does not per se require hospitalisation of the study patients. Any required hospitalisation during the study participation must be assessed for the occurrence of adverse events.

Medical surveillance during the study

The study is conducted under close medical surveillance. The patients are instructed to inform the investigator without any delay of any perceived negative change of their medical condition.

14.3 Serious Adverse Events

14.3.1 Definitions

All SAEs (as defined below) regardless of treatment group or suspected relationship to the study medication must be reported immediately (within 24 hours of the investigator's knowledge of the event) to Norgine's Global Pharmacovigilance Group, Fax: +44 (0)1895 453732. If the immediate report is done by telephone, this must be followed by detailed written reports using the SAE report form within 24 hours of the investigator's knowledge of the event.

An SAE is any AE that:

1. results in death,
2. is life threatening, that is any event that places the patient at immediate risk of death from the reaction as it occurred. It does not include a reaction that, had it occurred in a more severe form, might have caused death,
3. results in in-patient hospitalisation or prolongation of existing hospitalisation, excluding admission for social or administrative reasons,
4. results in a persistent or significant disability/incapacity, where disability is a substantial disruption of a person's ability to conduct normal life functions,
5. results in congenital anomaly/birth defect in the offspring of a patient who received the study medication,
6. is an important medical event that may not result in death, be life-threatening, or require hospitalisation when, based upon appropriate medical judgement, may jeopardise the patient and may require medical or surgical intervention to

prevent one of the outcomes listed in this definition. Examples of such medical events include allergic bronchospasm requiring intensive treatment in an emergency room or at home, blood dyscrasias or convulsions that do not result in in-patient hospitalisation, or the development of drug dependency or drug abuse.

Regardless of the above criteria, any additional AE that the sponsor or an investigator considers serious should be immediately reported to the sponsor and will be entered into Norgine's SAEs database system.

- Hospitalisation is defined as any in-patient admission (even if less than 24 hours). For in-patients, hospitalisation also includes transfer within the hospital to an acute/intensive care in-patient unit.
- Prolongation of hospitalisation is defined as any extension of an in-patient hospitalisation beyond the stay anticipated/required in relation to the original reason for the initial admission, as determined by the investigator or treating physician. For protocol-specified hospitalisation in clinical studies, prolongation is defined as any extension beyond the length of stay described in the protocol. Prolongation in the absence of a precipitating, treatment-emergent, clinical AE (i.e. not associated with the development of a new AE or worsening of a pre-existing condition) may meet criteria for "seriousness" but is not an adverse experience and thus is not subject to immediate reporting to the sponsor.
- Pre-planned or elective treatments/surgical procedures should be noted in the patient's screening documentation. Hospitalisation for a pre-planned or elective treatment/surgical procedure should not be reported as an SAE unless there are complications or sequelae which meet the criteria for seriousness described above.

14.3.2 Reporting requirements

Any SAE must be reported immediately (within 24 hours), independent of the circumstances or suspected cause, if it occurs or comes to the attention of the investigator at any time during the study period.

SAE report forms must be submitted to:

Global Pharmacovigilance
Norgine Ltd.
Norgine House, Widewater Place, Moorhall Road,
Harefield, Uxbridge, UB9 6NS, United Kingdom

Fax: +44-1895-453732

Email: global_pharmacovigilance@norgine.com

The sponsor's study physician can be contacted during office hours on:

Tel: +49-642 198 5222

The following dedicated 24-hour emergency number can be used to contact a Norgine study physician for resolution of urgent safety questions:

+44 (0) 1748 828 787.

Any SAE with a suspected causal relationship to the study medication occurring at any other time after completion of the study must be promptly reported.

14.3.3 Mandatory information for reporting an SAE

The following information is the minimum that must be provided to the sponsor pharmacovigilance contact within 24 hours for each SAE:

- Study number
- Centre number
- Subject number
- AE
- investigator's name and contact details

The additional information included in the SAE form must be provided to the sponsor or representative as soon as it is available. Upon receipt of the initial report, the sponsor will ask for the investigator's causality assessment if it was not provided with the initial report.

The investigator should report a diagnosis or a syndrome rather than individual signs or symptoms. The investigator should also try to separate a primary AE considered as the foremost untoward medical occurrence from secondary AEs which occurred as complications.

14.3.4 Reporting exemptions

There are no reporting exemptions.

14.4 Pregnancy

Pregnancy per se does not classify as an AE. However, AEs related to a pregnancy have to be reported like any other AEs and pregnancy must be reported using the Pregnancy Report Form. Patients should be instructed to notify the investigator in case of pregnancy during the study or within 30 days after discontinuing study medication.

If pregnancy is suspected during the study, study medication must be immediately withheld until the result of a laboratory pregnancy test is available.

Should pregnancy be confirmed, the subject must be withdrawn from study participation and the sponsor must be notified within 24 hours of the day the investigational site becomes aware of the pregnancy.

A pregnancy has to be followed-up until after the delivery of the child and reported to the sponsor accordingly.

14.5 Deaths

All AEs resulting in death either during the study period or within 28 days after the last dose of the study medication must be reported as an SAE within 24 hours of the investigator's knowledge of the event.

The convention for recording death is as follows:

- AE term: lead cause of death (e.g. multiple organ failure, pneumonia, myocardial infarction).
- Outcome: fatal.

The only exception is if the cause of death is unknown (i.e. sudden or unexplained death), in which case the AE term may be 'Death' or 'Sudden death'.

14.6 Discontinuation/Withdrawal due to Adverse Events/Serious Adverse Events

Discontinuation/withdrawal due to AEs should be distinguished from discontinuation/ withdrawal for other reasons.

Discontinuation of the study medication due to a SAE must be reported immediately to the sponsor's Representative. In all cases the investigator must ensure the patient receives appropriate medical follow-up.

14.7 Reporting to Competent Authorities/IECs/IRBs/Other Investigators

The sponsor will submit reports of SUSARs occurring during the study to the Competent Authorities, IECs, IRBs and other investigators concerned by the study medication. Reporting will be done in accordance with the applicable regulatory requirements and Norgine procedures.

14.8 Safety Monitoring Plan

A safety monitoring plan will be generated for the study to document adverse event reporting requirements.

15 STATISTICAL METHODS

15.1 Determination of Sample Size

A difference in PDR of 14% of MOVIPREP® against CitraFleet® can be assumed as a starting point.

A PDR of 44% is expected for MOVIPREP® versus 30% for CitraFleet®. This PDR detection is based on studies documented in literature (Piarra Blanco, Lee) and the 14% difference on studies published by Cohen (2010) (39% vs. 20%) and Matro (2010) (37% vs. 26%) [1,6,8,10,11].

However, these assumptions are based on study populations described in the literature which do not exactly reflect the characteristics of the study population in this study. It can be expected that the difference in the detection rates is lower due to a decreased difference in cleansing capability between the two preparations. Therefore, the time point of the interim analysis would be a 'best case' scenario and lead to termination of the study due to early success if the basic assumption for the difference in rates is true. On the other hand, the interim analysis will give the opportunity to increase the sample size within a realistic range, or to stop the study due to futility.

An interim analysis is to be conducted when data for the primary endpoint (i.e. PDR) are available for the first 400 patients included into the study.

A two group Chi-square test with a 0.05 two-sided significance level will have 80% power to detect the difference between a Group 1 proportion, p_A, of 0.440 and a Group 2 proportion, p_B, of 0.300 (odds ratio of 0.545) when the sample size in each group is 186. Controlling for a drop-out rate of about 7%, approximately 400 patients will be required to be randomised into this study with a randomisation rate of 1:1 up to the interim analysis.

The interim analysis shall indicate whether the assumptions at the beginning of the study have been adequate. Either premature termination of the study is possible due to success or futility, or continuation of the study and recalculation of the sample size.

Based on the results of the interim analysis, the assumptions for the sample size calculation will be checked and the sample size will be recalculated and adapted if necessary.

15.2 Definition of Study Populations for Analysis

Evaluation of efficacy is performed for the ITT population and for the PP population. The safety evaluation is performed for the safety population.

15.2.1 Safety population

The safety population includes all patients for whom it cannot be excluded that they took the study medication at least once.

15.2.2 Intention to treat population

The intention to treat (ITT) population includes all patients for whom it cannot be ruled out that they took the study medication at least once, who are eligible for the study, and for whom post baseline data are available.

15.2.3 Per protocol population

The per protocol (PP) population includes all patients of the ITT population who finished the study according to protocol or who terminated the study prematurely because of an event related to the study medication (per definition: premature termination because of an adverse event, that was classified by the investigator as at least ‘possibly related’ to the study medication).

15.3 Primary Endpoint

The primary efficacy endpoint will be the total PDR defined as number of patients with at least one polyp or flat lesion, as recorded by the gastroenterologist performing the colonoscopy in relation to the total analysis population.

15.4 Secondary Endpoints

The key secondary endpoint of this study is:

1. ADR, (including flat lesions) defined as number of patients with at least one adenoma as recorded by the pathologist in relation to the total analysis population.

Additional secondary endpoints are:

2. ADR and PDR by location:
    - left-sided (rectum, colon sigmoideum, colon descendens, left half of colon transversum)
    - right-sided (right half of colon transversum, colon ascendens, caecum)
3. Cancer detection rate, defined as number of patients with at least one malignancy in relation to the total analysis population.
4. Flat lesion only detection rate.
5. Advanced risk lesion detection rate (lesions >1 cm, low grade and/or villous).
6. Colonoscopy completion rate.
7. Colon cleansing quality, as reported by the gastroenterologist, according to the Harefield Cleansing Scale©.
8. Acceptability and tolerability of the study medication (according to the questionnaires included in Appendix 20.3).

15.5 Procedures for Handling of Missing or Spurious Data

Not applicable.

15.6 Statistical Methods

15.6.1 Analyses of demographic data and baseline characteristics

Demographic data (age, gender, ethnic origin) and baseline characteristics (medical history and current illnesses, concomitant medication, vital signs, results of physical examination) will be analysed descriptively.

15.6.2 Analyses of efficacy variables

In the context of the primary endpoint, the number of patients with at least one polyp will be determined and also percentage based on the total analysis population. PDR and ADR will be compared between the two treatment groups using the Chi-square test two-sided on an α-level of 5%. Additionally, the 95% confidence intervals two-sided for PDRs/ADRs will be determined for the two treatment groups, and for the difference in PDR/ADR rates between treatment groups the 95% confidence interval two-sided will be determined as well.

The analysis will be carried out primarily based on the ITT population and for sensitivity reasons also based on the PP population.

The ADR according to patients with at least one adenoma as recorded by the gastroenterologist and confirmed by the pathologist will be analysed analogously to the primary endpoint, i.e. group comparison will be based on the Chi-square test, and additionally the 95% confidence intervals for single rates and for the difference in rates will be determined.

The incidence of cancers will be analysed descriptively. Frequencies and percentages will be determined by treatment group and overall.

Left sided (rectum, colon sigmoideum, colon descendens, left half of colon transversum) and right sided (right half of colon transversum, colon ascendens, and caecum) polyps and adenomas will be analysed using frequency tables showing absolute frequencies and percentages overall and by treatment group.

The quality of cleansing will be evaluated based on the physician's evaluation following the Harefield Cleansing Scale©. In general, for continuous variables summary statistics will be provided by treatment group and overall showing statistics for location and variation. For discrete variables frequency tables will be provided.

15.6.3 Safety and tolerability data

Acceptability and tolerability of the preparation, according to the PDF, will be analysed descriptively providing summary statistics by treatment group and overall. A

standardised patient questionnaire will assess all AEs related to the preparation. Additionally, these AEs will be transferred to the CRF documentation and will be analysed accordingly. Summary statistics will be provided based on the MedDRA coding by preferred term (PT) and system organ class (SOC), and also by intensity and causal relationship.

15.7 Interim Analysis

An interim analysis is planned after data for the primary endpoint are available for 400 patients are included into the study, so that data for the primary endpoint are available for at least 372 patients. The purpose of the interim analysis is to indicate whether the assumptions made at the beginning of the study have been adequate. In consequence, either termination of the study is possible due to success or futility, or continuation of the study and recalculation of the sample size. Based on the results of the interim analysis, the assumptions for the sample size calculation will be checked and the sample size will be recalculated and adapted if necessary up to an upper limit of 800 patients.

In addition, the interim analysis and the final analysis will be performed based on an adaptive design as described by Bauer and Köhne (1994) [9].

15.8 Subgroup analyses

Subgroup analyses can be performed as long as sufficient data are available. The primary endpoint will additionally be investigated for the subgroup of patients showing sufficient colon cleansing for complete colonoscopy.

16 ETHICAL CONSIDERATIONS AND INSURANCE

16.1 Subject Information and Informed Consent

Prior to study start, the patients will receive a full explanation of the study, study medication, and all possible side effects in writing and additional verbal explanations. All patients must fully understand the explanations before signing the informed written consent. The consent form has to be signed and dated personally by the patient and the investigator, which will be stored with the investigator for a period of 15 years. The patient will receive a copy of the signed consent form. The monitor will check the consent form for all screened patients to ensure that consent has been granted prior to the conduct of any study specific procedures.

16.2 Ethics Committee(s)

Prior to study start, the protocol, SmPC, informed consent form, curriculum vitae of the coordinating investigator, and a copy of the insurance policy will be submitted to the relevant Ethics Committee. Upon receipt of the above mentioned approvals the responsible authorities will be informed of the start of patient recruitment.

All SAEs occurring during the study, which might endanger the subject or the entire study, will be reported to the involved Ethics Committee and the higher authorities.

16.3 Amendments to the Protocol

Changes to the protocol which may have an influence on the scientific rationale, the risk/benefit ratio, the health aspects of the healthy volunteers, or constitute a substantial change to the original application will require re-submission to the Ethics Committee. Patients who are actively participating in the study will be re-consented if required. The signed amendments will be attached to the protocols in use. The amendments will replace the relevant sections in the original protocol.

Administrative or technical changes of the protocol which have no influence on the health aspects of the patients will also require a change to the protocol. This change of the protocol will be submitted to the Ethics Committee for notification only and will be attached to the protocol in use.

16.4 Insurance

The patient will be informed of the insurance coverage and the address of the insurance company. According to the insurance conditions, the policy holder or authorised third party has to inform the patient with regard to the patient's insurance and the relevant obligations, especially concerning the obligations of the patients as:

1. The patients must not have alternative medical treatment (except in cases of

emergency or approved by the investigator).

2. An impairment of health which might occur due to the clinical study must be forwarded to the insurance company immediately via Norgine.

3. The notification of Norgine should be handled by the investigator.

16.5 Legal Aspects

This study will be performed according to the principles of the Declaration of Helsinki (Seoul, October 2008). Each patient will be informed using the patient information form, as attached, that participation in the study is voluntary and they may withdraw from the study without giving any reason and without loosing any benefit. Patients will be fully informed regarding the study medication and possible side effects, as well as a full explanation regarding the relevance and nature of the study. They will be informed of their obligations with regard to the insurance coverage. According to the insurance policy the patient is obliged not to have an alternative medical treatment during the clinical study unless in the case of an emergency or the investigator has been informed and agreed before hand. The patient must not participate in another clinical study and has to notify an impairment of health which might occur due to the clinical study immediately to the investigator, Norgine Ltd. or the insurance company. Prior to participating in the study, patients are required to sign the consent form to declare voluntary participation in the study, follow all obligations stated in the protocol and willingness to follow the instructions of the investigator. They must be prepared to answer questions which are raised during the conduct of the study. Patients declare their consent that the data obtained during the clinical study might be forwarded for checks to the sponsor, the relevant authorities or relevant federal authorities.

The applicable German Federal Drug Law will be fully followed during the clinical study. All physicians involved in the clinical study are obliged to conduct and carry out the study according to the Guidelines of Good Clinical Practice (ICH-GCP), EU Clinical Trials Directive and the Declaration of Helsinki. Similarly, all employees from Norgine Ltd. involved in the clinical study will strictly adhere to ICH-GCP and all requirements stated in the EU Clinical Trials Directive.

17 GENERAL OBLIGATIONS, AGREEMENTS AND ORGANISATION

17.1 Investigators Brochure

The study centre will be informed of the non-clinical and clinical medical knowledge by the sponsor. As soon as new results are available the Principal Investigators will be updated with respect to new safety information. The reference information will be provided in the form of the currently approved SmPCs of MOVIPREP® and CitraFleet®.

17.2 Data Protection and Confidentiality of the Investigator

The name of the patient and any other confidential data will be protected by the investigator. Should the patient's name need to be identified, this will only be revealed following the legal obligation of the physician. The investigator will particularly pay attention that the patient names are obscured on CRFs or additional documents provided to the sponsor.

17.3 Source Data Documentation within the CRF

The study documents will be source data for several parameters:

- The endoscopic and pathological reports will be source data for documentation of ADR, PDR, cancer detection rate, colonoscopy completion rate, and colon cleansing quality,
- The investigator rating for colon cleansing of each case in the CRF will be source data,
- PDFs completed by each patient will be regarded as source data.

17.4 CRFs and Handling

Norgine Ltd. will supply the study site with CRFs, and an Investigator Site File.

The CRFs will contain three part Non Carbon Requiring (NCR) copies in order to facilitate documentation. One page will remain with the investigator; the other two parts will be collected by the monitor. If any corrections or amendments become necessary, data clarification forms will be forwarded to the investigator. The respective completed PDFs will be collected, reviewed and attached to the corresponding CRF for collection by the monitor.

All CRFs, including non-completed pages and clinical study data, will be stored by Norgine Ltd. for a period of at least 15 years after termination of the study. The

consent forms and re-identification lists will be archived with the investigator for at least 15 years after termination of the study.

It is planned that some of the required study data will be recorded directly on the CRF (i.e. there will be no prior written or electronic record data), and the CRF will be considered source data.

17.5 Monitoring (Quality Control)

It is agreed that the conduct of the study will be monitored by a responsible monitor of Pierrel Research. The above mentioned persons as well as sponsor representative and authorised members of the relevant authorities are permitted to review the various documents of the study on request (including, but not limited to, the protocol, CRF and patient records). The above mentioned persons will strictly follow the legal requirements as required in the relevant data protections laws.

All documentation forms should be completed using a black ball-point pen, and must be legible. The investigator is advised to transfer any information which is requested in the CRF immediately in order to minimise the time required for completion of CRFs. This will enable a timely handover of completed parts of CRFs.

The study monitor will visit the centre regularly in order to check the accuracy of the CRFs. Monitoring visits will be conducted at the study site depending on the rate of enrolment. It is the monitor's responsibility to inspect the completed CRFs at regular intervals throughout the study to verify adherence to the protocol, the completeness, accuracy and consistency of the data. The monitor must have access to the documentation charts and medical records needed to verify the entries on the CRF. The investigator must agree to co-operate with the monitor to ensure that any questions or problems detected during the monitoring visit are resolved. In order to allow regular monitoring, the next date for the monitoring visit should be agreed on during the actual monitoring visit. Investigators and monitors should attempt to maintain the monitoring visit schedule to ensure that agreed dates for monitoring visits are not postponed. Monitoring visits will be conducted at the study site depending on the actual rate of subject enrolment.

The investigator may delegate the authority for completing the CRF and fulfilling other study functions to named individuals. A signature form must be completed and maintained up to date to document this delegation of responsibility. The investigator or authorised staff will sign and date the indicated places of the CRF, including the returned completed PDFs, to indicate a thorough inspection of the data on the CRF, completeness and certification of the content. Any errors on the CRF should be crossed by a single line but not obliterated, the correction and reason for correction inserted where appropriate, and the change initialled and dated by the investigator or an authorised member of the study staff. The use of correction fluid or tape is prohibited. Data Query Forms will be forwarded to the investigator to resolve any inconsistencies.

In order to ensure the quality of clinical data across all patients and sites, a clinical data

review ("in house" monitoring) will be performed on the completed CRF data by Pierrel Research. During this review, patient data will be checked for consistency, omissions and any apparent discrepancies. In addition, the data will be reviewed for adherence to the protocol and ICH-GCP. To resolve any questions from the clinical data review, written data queries and/or corrections will be forwarded to the investigator via the monitor for completion and return.

The relevant staff of Norgine Ltd. or other authorised persons will be permitted to review any clinical or laboratory equipment following agreement with the investigator.

17.6 Audit (Quality Assurance) and Inspection

This study may undergo a quality assurance audit, as part of a routine site audit program. It is agreed following the ICH-GCP guidelines, an independent auditing of all facilities used in the study (e.g. laboratories) and data collected can be performed by a staff member of Norgine Ltd., other persons authorised by Norgine Ltd. (who are not involved in the study) or members of the relevant authorities. During this audit, access to all study related records is required to compare the original subject files with the information given in the CRF, adherence to the protocol and to the relevant guidelines of ICH-GCP. Any clinical or laboratory equipment can also be audited. A certificate of the auditing will be written and signed by the auditor.

17.7 Storage of Study Documents and Investigator Site File

The investigator will be supplied with an Investigator Site File for permanent storage of all documents applicable to this clinical study.

17.8 Confidentiality

All study documentation and CRF data must be kept strictly confidential and may not be disclosed to third parties. All members of staff at the study site and within Norgine are obliged to comply with this requirement.

17.9 Notification of authorities - BfArM and Other Governmental Institutions

The participation of investigators in a clinical study will be notified to the competent higher federal authority and to the competent local authorities, as legally required in Germany.

The data regarding pharmacology and toxicology, the study protocol, name and address of investigator(s) and the approval by the Ethics Committee(s) will be deposited with the federal authority, as legally required. The higher authorities will further be supplied with all relevant documents as legally required.

17.10 Publication

After completion of the study, an integrated Clinical Study Report (CSR) will be written based on the statistical analysis and the review of the analysis with regard to the medical results. The basis for the CSR is all aspects as detailed in this protocol. The final integrated report will be signed by the author of the report, the coordinating investigator and the assigned representative of Norgine Ltd.).

Publication(s) of the results is encouraged after appropriate time for review and written agreement by Norgine Ltd. It is intended that all investigators actively involved in the study will be co-authors of the publication/presentation with the position been determined by the efforts for the study (i.e. number of subjects enrolled). The list of authors will include the coordinating investigator and the names of the investigators who have studied at least 15 patients, with one name for each centre. Norgine Ltd will also be mentioned. If the journal does not permit a full list of all authors in the title section, the maximal number of authors will be listed by their input into the study and the remaining investigators will be included into an acknowledgement. Norgine Ltd will be authorised to check any manuscripts to be published at least six weeks before despatch to a journal.

18 SCHEDULE OF ASSESSMENT/STUDY FLOWCHART

	Screening	Colonoscopy visit	End of study
	Visit 1 (Day 0)	Visit 2 (up to Day 30)	Follow-up period (for 30 days after Visit 2)
Demographic data	X		
Medical history (and concomitant diseases)	X		
Concomitant medication	X	X	
Body weight, height, blood pressure, pulse rate, body temperature	X	X	
Physical examination	X		
Informed consent	X		
Eligibility for colonoscopy	X		
Pregnancy test	X		
Inclusion and exclusion criteria	X		
Randomisation	X		
Dispensing of study medication*	X		
Harefield Cleansing Scale©		X	
Distribution of PDF	X		
PDF collection and review		X	
Colonoscopy		X	
Drug accountability		X	
Adverse events		X	
Follow-up of adverse events			X

*Administration of the study medication: performed by the patients, starting on the day before Visit 2

19 References

1. Cohen LB, Sanyal SM, von Althann C, Bodian C, Whitson M, Bamji N et al. Clinical trial: 2-L polyethylene glycol-based lavage solutions for colonoscopy preparation – a randomized, single-blind study of two formulations. Aliment Pharmacol Ther 2010; 32:637-44.
2. Froehlich F, Wietlisbach V, Gonvers JJ, Burnand B, Vader JP. Impact of colonic cleansing on quality and diagnostic yield of colonoscopy: the European Panel of Appropriateness of Gastrointestinal Endoscopy European multicenter study. Gastrointest Endosc. 2005; 61(3):378-84.
3. Harewood GC, Sharma VK, de Garmo P. Impact of colonoscopy preparation quality on detection of suspected colonic neoplasia. Gastrointest Endosc. 2003; 58(1):76-9.
4. Kaminski MF, Regula J, Kraszewska E, Polkowski M, Wojciechowska U, Didkowska J, et al. Quality Indicators for colonoscopy and the risk of interval cancer. N Engl J Med 2010; 362(19):1795-803.
5. Rex DK, Petrini JL, Baron TH, Chak A, Cohen J, Deal SE, et al. Quality indicators for colonoscopy. Gastrointest Endosc 2006; 63:Suppl:16-28.
6. Sanaka MR, Deepinder F, Thota PN, Lopez R, Burke CA. Adenomas are more often detected in morning than in afternoon colonoscopy. An J Gastroenterol 2009; 104(7):1659-64.
7. Shaukat A, Oancea C, Bond JH, Church TA, Allen JI. Variation in detection of adenomas and polyps by colonoscopy and change over time with a performance improvement program. Clin Gastroenterol Hepatol 2009; 7(12):1335-40.
8. Matro R, Shnitser A, Spodik M, Daskalakis C, Katz L, Murtha A, Kastenberg D. Efficacy of morning-only compared with split-dose polyethylene glycol electrolyte solution for afternoon colonoscopy: a randomized controlled single-blind study. Am J Gastroenterol 2010; 105(9):1954-61.
9. Bauer P, Köhne K. Evaluation of experiments with adaptive interim analyses. Biometrics 1994; 50:1029-41.
10. Parra-Blanco A, Nicolas-Perez D, Gimeno-Garcia A, Grosso B, Jimenez A, Ortega J, Quintero E. The timing of bowel preparation before colonoscopy determines the quality of cleansing, and is a significant factor contributing to the detection of flat lesions: a randomized study. World J Gastroenterol. 2006 Oct 14;12(38):6161-6.
11. Lee A, Iskander JM, Gupta N, Borg BB, Zuckerman G, Banerjee B, Gyawali CP. Queue Position in the Endoscopic Schedule Impacts. Effectiveness of Colonoscopy. Am J Gastroenterol. 2011 Mar 29 (electronic publication).

20 APPENDICES

20.1 PARIS Classification for Colon Polyps

Extract from the IIS proposal

Adenomatous lesions are classified by appearance according to the Paris classification in peduncular, sessile (together usually called polypoid lesions), flat or depressed lesions (see appendix). Non-polypoid adenomas that are flat or depressed account for 22 to 36 percent of identified adenomas.[8-10] Flat and depressed lesions are difficult to detect as they are recognizable only by subtle distortion of the mucosal pattern and special stains.[8] Large flat adenomas may be more likely to contain dysplastic changes or cancer than polypoid ones of comparable size. [10] Other types of colonic polyps are hyperplastic polyps which up to recently were considered not to have a tendency to become malignant, serrated adenomas which are polyps with both hyperplastic and adenomatous features at histological examination, and inflammatory polyps which are seen in patients after active inflammation mainly due to inflammatory bowel disease. To determine the type of polyp, all polyps are removed during colonoscopy for pathological evaluation.

8. Rembacken BJ, Fujii T, Cairns A, Dixon MF, Yoshida S, Chalmers DM, et al. Flat and depressed colonic neoplasms: a prospective study of 1000 colonoscopies in the UK. Lancet. 2000 Apr 8;355(9211):1211-4.

9. Saitoh Y, Waxman I, West AB, Popnikolov NK, Gatalica Z, Watari J, et al. Prevalence and distinctive biologic features of flat colorectal adenomas in a North American population. Gastroenterology. 2001 Jun;120(7):1657-65.

10. Soetikno RM, Kaltenbach T, Rouse RV, Park W, Maheshwari A, Sato T, et al. Prevalence of nonpolypoid (flat and depressed) colorectal neoplasms in asymptomatic and symptomatic adults. Jama. 2008 Mar 5;299(9):1027-35.

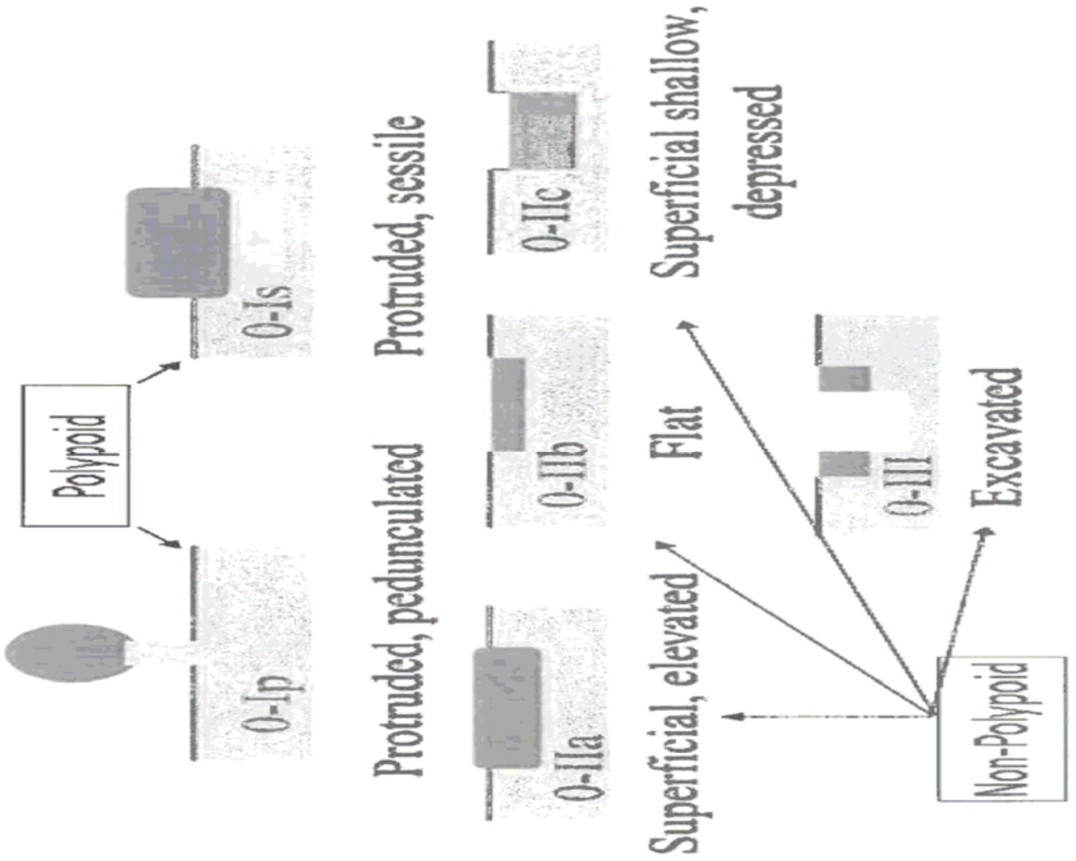

The Paris Endoscopic Classification of Superficial Neoplastic Lesions: Esophagus, Stomach, and Colon
November 30 to December 1, 2002

20.2 Labels for Study Medication:

Each formulation will be packed and will be labelled in accordance with European Guidelines (F2/BL D (2003)) Volume 4 Good Manufacturing Practice Annex 13). A template label is illustrated below. The final label will be found in the Trial Master File.

20.2.1 Label for MOVIPREP®

Label for secondary pack:

Study Number: NOR-01/2011 (PDR)
EudraCT No.: 2011-002364-25
Treatment Number: *XXX*
Batch Number: *XXXXXX*
Expiry/Use by Date *DD MMM YYYY*
Sponsor: Norgine Ltd., Norgine House, Widewater Place, Moorhall Road, Harefield, Uxbridge, UB9 6NS, United Kingdom Tel +44 1895 826600
CRO: Pierrel Research Germany GmbH, Zeche Katharina 6, 45307 Essen, Germany Tel: +49 201 89 90-0

MOVIPREP® powder for oral solution in sachets

Contents: two sachets A and two sachets B of MOVIPREP®.

Sachet A contains 112 g powder of which:

Macrogol 3350	100 g
Sodium Sulphate Anhydrous	7.500 g
Sodium Chloride	2.691 g
Potassium Chloride	1.015 g
Flavouring and sweeteners	0.690 g

This product contains 0.233 g aspartame per sachet A

Sachet B contains 10.6 g powder of which:

Ascorbic Acid	4.700 g
Sodium Ascorbate	5.900 g

Directions for use:
Follow the reconstitution instructions on the information leaflet.
Take one litre of MOVIPREP® in the evening before and one in the early morning of the examination.
Add the contents of ONE Sachet A and ONE sachet B into the shaker.
Pour in water up to the one litre mark and stir until all the powder has dissolved and the MOVIPREP® solution is clear or slightly hazy

Store sachets below 25 ºC in the original package.
Stored reconstituted solution below 25 ºC, solution may be refrigerated.
Keep covered, use the solution within 24 hours.
Return unused medication to the Sponsor.

Investigator: ____________________________
Address ____________________________
Tel ____________________________
For Clinical Trial Use Only
Keep out of reach and sight of children.

Sachet A label:

Study Number: NOR-01/2011 (PDR)
EudraCT No: 2011-002364-25
Treatment Number XXX **SACHET A**
Batch Number *XXXXXXXXX*
Expiry/Use By Date *DD MMM YYYY*

Contents: contains 112. g powder for oral solution of which:

Macrogol 3350	100 g
Sodium Sulphate Anhydrous	7.500 g
Sodium Chloride	2.691 g
Potassium Chloride	1.015 g
Flavouring and sweeteners	0.892 g

Directions for use: Add the contents of one sachet A and one sachet B to the shaker. Pour in water up to the 1 litre mark and stir until all of the powder has dissolved.

Sponsor: Norgine Limited
CRO: Pierrel Research Germany GmbH

Sachet B label:

Study Number: NOR-01/2011 (PDR)
EudraCT No: 2011-002364-25
Treatment Number XXX **SACHET B**
Batch Number *XXXXXXXXX*
Expiry/Use By Date *DD MMM YYYY*

Contents: contains 10.6 g powder for oral solution of which:

Ascorbic Acid	4.700 g
Sodium Ascorbate	5.900 g

Directions for use: Add the contents of one sachet A and one sachet B to the shaker. Pour in water up to the 1 litre mark and stir until all of the powder has dissolved.

Sponsor: Norgine Limited
CRO: Pierrel Research Germany GmbH

20.2.2 Label for Sodium Picosulfate and Magnesium Citrate preparation (CitraFleet®)

Label secondary pack:

Study Number: NOR-01/2011 (PDR)
EudraCT No.: 2011-002364-25
Treatment Number: *XXX*
Batch Number: *XXXXXX*
Retest/Expiry/Use by Date *DD MMM YYYY*
Sponsor: Norgine Ltd., Norgine House, Widewater Place, Moorhall Road, Harefield, Uxbridge, UB9 6NS, United Kingdom Tel +44 1895 826600
CRO: Pierrel Research Germany GmbH, Zeche Katharina 6,
45307 Essen, Germany Tel: +49 201 89 90-0

CitraFleet® powder for oral solution in sachet

Contents: two sachets of CitraFleet®.

Each sachet 15.08 g contains the following active ingredients:

Sodium picosulfate	10.0 mg
Light Magnesium Oxide	3.5 g
Citric Acid Anhydrous	10.97 g

This product contains 195 mg magnesium
Additional ingredients (0.405 g):
Potassium hydrogen carbonate
Saccharin sodium
Lemon Flavour

Directions for use:
Follow the reconstitution instructions on the information leaflet.
Take one sachet of CitraFleet® in the morning (before 8am) and the second sachet 6 to 8 hours later.
Reconstitute the contents of one sachet in a cup of water (approximately 150 ml). The resulting solution appears turbid. Stir for 2-3 minutes and drink the solution. If it becomes hot, wait until it cools sufficiently to drink.
To avoid dehydration an additional 250 mL of clear liquid should be consumed per hour during the washout period.

Store sachets below 25 ºC, do not refrigerate or freeze. Store in the original package.
Use immediately after reconstitution.
Return unused medication to the Sponsor.

Investigator: ______________________________
Address ______________________________
Tel ______________________________
For Clinical Trial Use Only
Keep out of reach and sight of children.

Sachet label:

Study Number: NOR-01/2011 (PDR)
EudraCT No: 2011-002364-25
Treatment Number XXX
Batch Number *XXXXXXXXX*
Retest/Expiry/Use By Date *DD MMM YYYY*

Contents: contains 15.08 g powder for oral solution of which:

Sodium picosulfate	10.0 mg
Light Magnesium Oxide	3.5 g
Citric Acid Anhydrous	10.97 g

This product contains 195 mg magnesium

Directions for use: Add the contents of this sachet to a cup of water (approximately 150 ml). The resulting solution appears turbid. Stir for 2-3 minutes and drink the solution. If it becomes hot, wait until it cools sufficiently to drink.
To avoid dehydration an additional 250 mL of clear liquid should be consumed per hour during the washout period.

Sponsor: Norgine Limited
CRO: Pierrel Research Germany GmbH

20.3 Template for Patient Documentation Form

20.3.1 Patient documentation form for MOVIPREP®

(continued on next page)

NORGINE	Date [Datum]<br><br>I__I__I I__I__I I__I__I__I__I<br><br>**PATIENT DOCUMENTATION FORM [STUDIEN DOKUMENTATION]**<br><br>**QUESTIONNAIRE**<br>**[FRAGEBOGEN]**	NOR-01/2011 (PDR)<br><br>I__I__I - I__I__I__I<br>Patient Number<br>[Patientennummer]

Treatment Number:
[Behandlungsnummer] ☐☐☐

Study Centre:
[Studienzentrum] ☐☐

Protocol: NOR-01/2011 (PDR)

This questionnaire must be completed during the gut cleansing intake and prior to the planned colonoscopy
[Dieser Fragebogen muss während Ihrer Darmvorbereitung vor der geplanten Koloskopie ausgefüllt werden.]

Dear Study Participant:

During this clinical trial, you will have a screening colonoscopy. You will need to consume the MOVIPREP® solution to prepare your colon for this procedure. Please complete the enclosed questionnaire during the gut preparation and prior to the planned colonoscopy.

This questionnaire is essential to assess the clinical tolerance and acceptability of the gut preparation prior to the colonoscopy.

Your doctor will review the questionnaire to make sure all questions are answered, which is important for the analysis of the study results. All information that you provide in this questionnaire is strictly confidential.

[Lieber Studienteilnehmer:
Sie werden eine Koloskopie im Rahmen einer Darmkrebsvorsorgeuntersuchung haben. Für die Koloskopie müssen Sie den Darm vorbereiten. Wir möchten Sie bitten, während Ihrer Darmvorbereitung den folgenden Fragebogen vollständig auszufüllen.

Der Fragebogen ist wichtig für die Auswertung der Studie, da hiermit die Verträglichkeit und Akzeptanz der Darmvorbereitung erfasst wird. Ihr Arzt wird den Fragebogen auf Vollständigkeit überprüfen.

Alle Informationen unterliegen der Geheimhaltung Ihrer Daten im Rahmen der Studie.]

NORGINE	Date [Datum]<br>I__I__I I__I__I I__I__I__I__I<br>**PATIENT DOCUMENTATION FORM [STUDIEN DOKUMENTATION]**<br>**QUESTIONNAIRE [FRAGEBOGEN]**	NOR-01/2011 (PDR)<br>I__I__I - I__I__I__I<br>Patient Number [Patientennummer]

Study Documentation during the intake of MOVIPREP® [Studien Dokumentation während der Einnahme der Darmspüllösung MOVIPREP®]

Intake of the first litre of the MOVIPREP® gut cleansing solution on the evening before the planned colonoscopy:

[Einnahme des ersten Liters der Darmspüllösung MOVIPREP® am Vorabend der geplanten Koloskopie:]

When did you drink the first litre?
[Wann haben Sie den ersten Liter getrunken?]

☐☐ ☐☐ ☐☐ From [Von] ☐☐:☐☐ to [Bis] ☐☐:☐☐

Plan: between [zwischen] 16:00 pm [nachmittags] to [bis] 23:00 pm [abends]

Day [Tag | Month Monat | Year Jahr | Hour Stunde | Min. Min. | Hour Stunde | Min. Min.]

Was the complete litre drunk?
[Haben Sie den gesamten Liter getrunken?]

☐ Yes [Ja]

☐ No, if so, why not: ____________________________
[Nein, weshalb nicht :]

How much was left? ☐☐☐☐ mL
[Wieviel ist übrig geblieben?]

NORGINE	Date [Datum]<br>I__I__I I__I__I I__I__I__I__I<br>**PATIENT DOCUMENTATION FORM [STUDIEN DOKUMENTATION]**<br>**QUESTIONNAIRE [FRAGEBOGEN]**	NOR-01/2011 (PDR)<br>I__I__I - I__I__I__I<br>Patient Number [Patientennummer]

How much additional clear liquid did you consume?
[Wieviel an zusätzlicher klarer Flüssigkeit haben Sie getrunken?]

☐☐☐☐ mL From [Von] ☐☐:☐☐ to [Bis] ☐☐:☐☐

Plan: at least 500 mL [mindestens 500 ml]

Amount [Menge — Hour Stunde — Min. Min. — Hour Stunde — Min. Min.]

How do you judge the taste of the gut cleansing solution after the first litre?
[Wie beurteilen Sie den Geschmack des ersten Liters der Darmspüllösung?]

0 Very bad [Sehr schlecht] ________________________________ 100 Very good [Sehr gut]

(Please indicate your taste judgment with a single vertical line on the above scale.)
[(Bitte zutreffendes Maß mit einem einzigen senkrechten Strich auf der obigen Skala kennzeichnen.)]

When did you experience the first bowel motion after taking the first litre of MOVIPREP®?
[Wann erfolgte der erste Stuhlgang nach Einnahme des ersten Liters der Darmspüllösung?]

Time [Uhrzeit]: ☐☐:☐☐

Hour [Std. — Min. Min.]

NORGINE	Date [Datum]<br><br>I__I__I I__I__I I__I__I__I__I<br><br>**PATIENT DOCUMENTATION FORM [STUDIEN DOKUMENTATION]**<br><br>**QUESTIONNAIRE**<br>**[FRAGEBOGEN]**	NOR-01/2011 (PDR)<br><br>I__I__I - I__I__I__I<br>Patient Number<br>[Patientennummer]

Which of the following symptoms occurred during the intake of the first litre of MOVIPREP®?
[Welche der folgenden Beschwerden traten bei Ihnen während der Einnahme des ersten Liters der Darmspüllösung auf?]

☐	☐	☐	☐	☐
None	Nausea	Vomiting	Abdominal Discomfort	Abdominal Pain
[Keine	Übelkeit	Erbrechen	Magen-beschwerden	Bauch-schmerzen]

How did you tolerate the first litre of the MOVIPREP® gut cleansing solution?
[Wie haben Sie den ersten Liter der MOVIPREP® Darmspüllösung vertragen?]

☐	☐	☐	☐	☐
Very Good	Good	Acceptable	Bad	Very Bad
[Sehr gut	Gut	Zufriedenstellend	Schlecht	Sehr schlecht]

NORGINE	Date [Datum]<br><br>I__I__I I__I__I I__I__I__I__I<br><br>**PATIENT DOCUMENTATION FORM [STUDIEN DOKUMENTATION]**<br><br>**QUESTIONNAIRE [FRAGEBOGEN]**	NOR-01/2011 (PDR)<br><br>I__I__I - I__I__I__I<br>Patient Number [Patientennummer]

Intake of the second litre of the MOVIPREP® gut cleansing solution on the morning of the day for the planned screening colonoscopy:

[Einnahme der Darmspüllösung MOVIPREP® am Morgen der geplanten Koloskopie]

When did you drink the second litre?
[Zeitangaben zur Einnahme des zweiten Liters]

☐☐ ☐☐ ☐☐ From [Von] ☐☐:☐☐ to [Bis] ☐☐:☐☐
Plan: starting [ab] 5:00 am [morgens]

Day [Tag | Month Monat | Year Jahr | Hour Stunde | Min. Min. | Hour Stunde | Min. Min.]

Was the complete litre drunk?
[Haben Sie den gesamten zweiten Liter getrunken?]

☐ Yes [Ja]

☐ No, if so, why not: ____________________________
[Nein, weshalb nicht:]

How much was left? ☐☐☐☐ mL
[Wieviel ist übrig geblieben?]

How much additional clear liquid did you consume?
[Wieviel an zusätzlicher klarer Flüssigkeit haben Sie getrunken?]

☐☐☐☐ mL From [Von] ☐☐:☐☐ to [Bis] ☐☐:☐☐
Plan: at least 500 mL [mindestens 500 ml]

Amount [Menge | Hour Stunde | Min. Min. | Hour Stunde | Min. Min.]

NORGINE	Date [Datum]<br><br>I__I__I I__I__I I__I__I__I__I<br><br>**PATIENT DOCUMENTATION FORM [STUDIEN DOKUMENTATION]**<br><br>**QUESTIONNAIRE [FRAGEBOGEN]**	NOR-01/2011 (PDR)<br><br>I__I__I - I__I__I__I<br>Patient Number [Patientennummer]

How do you judge the taste of the gut cleansing solution after the second litre?
[Wie beurteilen Sie den Geschmack des zweiten Liters der Darmspüllösung?]

0 ____ 100

Very bad [Sehr schlecht] Very good [Sehr gut]

(Please indicate your taste judgment with a single vertical line on the above scale.)
[(Bitte zutreffendes Maß mit einem einzigen senkrechten Strich auf der obigen Skala kennzeichnen.)]

Which of the following symptoms occurred during the intake of the second litre of MOVIPREP®?
[Welche der folgenden Beschwerden traten bei Ihnen während der Einnahme des zweiten Liters der Darmspüllösung auf?]

☐	☐	☐	☐	☐
None<br><br>[Keine	Nausea<br><br>Übelkeit	Vomiting<br><br>Erbrechen	Abdominal Discomfort<br>Magen-beschwerden	Abdominal Pain<br>Bauch-schmerzen]

How did you tolerate the second litre of the MOVIPREP® gut cleansing solution?
[Wie haben Sie den zweiten Liter der MOVIPREP® Darmspüllösung vertragen?]

☐	☐	☐	☐	☐
Very Good<br>[Sehr gut	Good<br>Gut	Acceptable<br>Zufriedenstellend	Bad<br>Schlecht	Very Bad<br>Sehr schlecht]

NORGINE	Date [Datum]<br><br>I__I__I I__I__I I__I__I__I__I<br><br>**PATIENT DOCUMENTATION FORM [STUDIEN DOKUMENTATION]**<br><br>**QUESTIONNAIRE**<br>**[FRAGEBOGEN]**	NOR-01/2011 (PDR)<br><br>I__I__I - I__I__I__I<br>Patient Number<br>[Patientennummer]

Overall ratings at the end of the gut preparation

[Zusammenfassende Beurteilung am Ende der Darmvorbereitung]

Did you experience problems whilst drinking gut cleansing solution?
[Hatten Sie Probleme beim Trinken der vorgesehenen Menge der Darmspüllösung?]

☐ None [Keine] ☐ Some [Einige] ☐ Many [Viele]

How did you tolerate the intake of MOVIPREP®?
[Wie haben Sie die Darmvorbereitung mit MOVIPREP® vertragen?]

____

0	100
Extremely poorly tolerated<br>[Überhaupt nicht vertragen]	Perfectly well tolerated<br>[Sehr gut vertragen]

(Please indicate your taste judgment with a single vertical line on the above scale.)
[(Bitte zutreffendes Maß mit einem einzigen senkrechten Strich auf der obigen Skala kennzeichnen.)]

How did you find MOVIPREP® as a gut cleansing solution?
[Waren Sie mit der Darmspüllösung zufrieden?]

____

0	100
Totally unacceptable<br>[Überhaupt nicht zufrieden]	Fully acceptable<br>[Sehr zufrieden]

(Please indicate your taste judgment with a single vertical line on the above scale.)
[(Bitte zutreffendes Maß mit einem einzigen senkrechten Strich auf der obigen Skala kennzeichnen.)]

NORGINE	Date [Datum]<br><br>I__I__I I__I__I I__I__I__I__I<br><br>**PATIENT DOCUMENTATION FORM [STUDIEN DOKUMENTATION]**<br><br>**QUESTIONNAIRE [FRAGEBOGEN]**	NOR-01/2011 (PDR)<br><br>I__I__I - I__I__I__I<br>Patient Number<br>[Patientennummer]

Were you satisfied with the whole gut cleansing preparation?
[Waren Sie zufrieden mit der gesamten Darmvorbereitung?]

0 — 100

Totally dissatisfied [Überhaupt nicht zufrieden] — Very satisfied [Sehr zufrieden]

(Please indicate the judgment of gut cleansing procedure with a single vertical line on the above scale.)
[(Bitte zutreffendes Maß mit einem einzigen senkrechten Strich auf der obigen Skala kennzeichnen.)]

The taste of the gut cleansing solution is:
[Der Geschmack der Lösung ist:]

☐ Good [Gut] ☐ Okay [Zufriedenstellend] ☐ Bad [Schlecht]

Drinking the MOVIPREP® solution, as explained in the instructions, was:
[Die Einnahme der Darmspüllösung gemäß der Anleitung war:]

☐ Very easy [Sehr einfach] ☐ Easy [Einfach] ☐ Quite difficult [Nicht so einfach] ☐ Very difficult [Schwierig]

My tolerance of the MOVIPREP® gut cleansing solution was:
Die Verträglichkeit der Darmvorbereitung mit der Darmspüllösung MOVIPREP® war:

☐ Very good [Sehr gut] ☐ Good [Gut] ☐ Okay [Zufriedenstellend] ☐ Bad [Schlecht] ☐ Very bad [Sehr schlecht]

Thank you for your help!
[Vielen Dank für Ihre Mitarbeit!]

Norgine Ltd. Confidential Page 8 of 9
Version 1.0 Final 27 June 2011 (EN/Ger)

NORGINE	Date [Datum]<br>I__I__I I__I__I I__I__I__I__I<br>**PATIENT DOCUMENTATION FORM [STUDIEN DOKUMENTATION]**<br>**QUESTIONNAIRE [FRAGEBOGEN]**	NOR-01/2011 (PDR)<br>I__I__I - I__I__I__I<br>Patient Number [Patientennummer]

Your colonoscopy is scheduled for:
[Ihre Koloskopie findet statt am:] ☐☐ ☐☐ ☐☐ **at [um]** ☐☐:☐☐
Day [Tag] Month [Monat] Year [Jahr] Hour [Std.] Min. [Min.]

Questionnaire reviewed by:
[Fragebogen überprüft:] ☐☐ ☐☐ ☐☐ ____________________
Day [Tag] Month [Monat] Year [Jahr] Investigator Signature [Unterschrift Prüfarzt]

Have any clinically relevant Adverse Events been identified? [Sind im bisherigen Verlauf klinisch relevante unerwünschte Ereignisse aufgetreten?] ☐ **Yes [Ja]** ☐ **No [Nein]**

Has the CRF been updated with the identified clinically relevant Adverse Events? ☐ **Yes** ☐ **No**

[Wurde die CRF-Dokumentation mit den identifizierten klinisch relevanten unerwünschten Ereignissen komplettiert?] **[Ja]** **[Nein]**

Date [Datum] ☐☐ ☐☐ ☐☐ ____________________
Day [Tag] Month [Monat] Year [Jahr] Investigator Signature [Unterschrift Prüfarzt]

20.3.2 Patient documentation form for CitraFleet®

(continued on next page)

NORGINE	PATIENT DOCUMENTATION FORM [STUDIEN DOKUMENTATION]<br><br>QUESTIONNAIRE [FRAGEBOGEN]	NOR-01/2011 (PDR)<br><br>I__I__I - I__I__I__I<br>Patient Number [Patientennummer]

Treatment Number: [Behandlungsnummer] ☐☐☐

Study Centre: [Studienzentrum] ☐☐

Protocol: NOR-01/2011 (PDR)

This questionnaire must be completed during the gut cleansing intake and prior to the planned colonoscopy
[Dieser Fragebogen muss während Ihrer Darmvorbereitung vor der geplanten Koloskopie ausgefüllt werden.]

Dear Study Participant:

During this clinical trial, you will have a screening colonoscopy. You will need to consume the CitraFleet® solution to prepare your colon for this procedure. Please complete the enclosed questionnaire during the gut preparation and prior to the planned colonoscopy.

This questionnaire is essential to assess the clinical tolerance and acceptability of the gut preparation prior to the colonoscopy.

Your doctor will review the questionnaire to make sure all questions are answered, which is important for the analysis of the study results. All information that you provide in this questionnaire is strictly confidential.

[Lieber Studienteilnehmer:
Sie werden eine Koloskopie im Rahmen einer Darmkrebsvorsorgeuntersuchung haben. Für die Koloskopie müssen Sie den Darm vorbereiten. Wir möchten Sie bitten, während Ihrer Darmvorbereitung den folgenden Fragebogen vollständig auszufüllen.

Der Fragebogen ist wichtig für die Auswertung der Studie, da hiermit die Verträglichkeit und Akzeptanz der Darmvorbereitung erfasst wird. Ihr Arzt wird den Fragebogen auf Vollständigkeit überprüfen.

Alle Informationen unterliegen der Geheimhaltung Ihrer Daten im Rahmen der Studie.]

NORGINE	Date [Datum]<br><br>I__I__I I__I__I I__I__I__I__I<br><br>**PATIENT DOCUMENTATION FORM [STUDIEN DOKUMENTATION]**<br><br>**QUESTIONNAIRE [FRAGEBOGEN]**	NOR-01/2011 (PDR)<br><br>I__I__I - I__I__I__I<br>Patient Number [Patientennummer]

Study Documentation during the Intake of CitraFleet® [Studien Dokumentation während der Einnahme der Darmspüllösung CitraFleet®]

Intake of the first 150 mL of the CitraFleet® gut cleansing solution in the morning of the day before the planned colonoscopy:

[Einnahme der ersten 150 ml der Darmspüllösung CitraFleet® am Morgen des Vortags der geplanten Koloskopie:]

When did you drink the first 150 mL?
[Wann haben Sie die ersten 150 ml getrunken?]

☐☐ ☐☐ ☐☐ Time [Zeit] ☐☐:☐☐
Plan: about [etwa um] 7:00 am [morgens]

Day [Tag | Month Monat | Year Jahr | Hour Stunde | Min. Min.]

Were the complete 150 mL drunk?
[Haben Sie die gesamten 150 ml getrunken?]

☐ Yes [Ja]

☐ No, if so, why not: ___________________________
[Nein, weshalb nicht :]

How much was left? ☐☐☐ mL
[Wieviel ist übrig geblieben?]

NORGINE	Date [Datum]<br><br>I__I__I I__I__I I__I__I__I__I<br><br>**PATIENT DOCUMENTATION FORM [STUDIEN DOKUMENTATION]**<br><br>**QUESTIONNAIRE [FRAGEBOGEN]**	NOR-01/2011 (PDR)<br><br>I__I__I - I__I__I__I<br>Patient Number [Patientennummer]

How much additional clear liquid did you consume?
[Wieviel an zusätzlicher klarer Flüssigkeit haben Sie getrunken?]

☐☐☐☐ mL From [Von] ☐☐:☐☐ to [Bis] ☐☐:☐☐

Plan: 250 mL per hour [250 ml pro Stunde]

Amount [Menge   Hour Stunde   Min. Min.   Hour Stunde   Min. Min.]

How do you judge the taste of the gut cleansing solution after the first 150 mL?
[Wie beurteilen Sie den Geschmack der ersten 150 ml der Darmspüllösung?]

____

0 Very bad [Sehr schlecht]   100 Very good [Sehr gut]

(Please indicate your taste judgment with a single vertical line on the above scale.)
[(Bitte zutreffendes Maß mit einem einzigen senkrechten Strich auf der obigen Skala kennzeichnen.)]

When did you experience the first bowel motion after taking the first 150 mL of CitraFleet®?
[Wann erfolgte der erste Stuhlgang nach Einnahme der ersten 150 ml der Darmspüllösung?]

Time [Uhrzeit]: ☐☐:☐☐

Hour [Std.   Min. Min.]

NORGINE	Date [Datum]<br><br>I__I__I I__I__I I__I__I__I__I<br><br>**PATIENT DOCUMENTATION FORM [STUDIEN DOKUMENTATION]**<br><br>**QUESTIONNAIRE [FRAGEBOGEN]**	NOR-01/2011 (PDR)<br><br>I__I__I - I__I__I__I<br>Patient Number<br>[Patientennummer]

Which of the following symptoms occurred during the intake of the first 150 mL of CitraFleet®?
[Welche der folgenden Beschwerden traten bei Ihnen während der Einnahme der ersten 150 ml der Darmspüllösung auf?]

☐	☐	☐	☐	☐
None	Nausea	Vomiting	Abdominal Discomfort	Abdominal Pain
[Keine	Übelkeit	Erbrechen	Magen-beschwerden	Bauch-schmerzen]

How did you tolerate the first 150 mL of the CitraFleet® gut cleansing solution?
[Wie haben Sie die ersten 150 ml der CitraFleet® Darmspüllösung vertragen?]

☐	☐	☐	☐	☐
Very Good	Good	Acceptable	Bad	Very Bad
[Sehr gut	Gut	Zufriedenstellend	Schlecht	Sehr schlecht]

NORGINE	Date [Datum]<br><br>I__I__I I__I__I I__I__I__I__I<br><br>**PATIENT DOCUMENTATION FORM**<br>**[STUDIEN DOKUMENTATION]**<br><br>**QUESTIONNAIRE**<br>**[FRAGEBOGEN]**	NOR-01/2011 (PDR)<br><br>I__I__I - I__I__I__I<br>Patient Number<br>[Patientennummer]

Intake of the second 150 mL of the CitraFleet® gut cleansing solution in the afternoon of the day before the planned screening colonoscopy:

[Einnahme der zweiten 150 ml Darmspüllösung CitraFleet® am Nachmittag des Vortages der geplanten Koloskopie]

When did you drink the second 150 mL?
[Wann haben Sie die zweiten 150 ml getrunken?]

☐☐ ☐☐ ☐☐ Time [Zeit] ☐☐:☐☐
Plan: between [zwischen] 2:00 pm and 4:00 pm [nachmittags]

Day [Tag | Month Monat | Year Jahr | Hour Stunde | Min. Min.]

Were the complete 150 mL drunk?
[Haben Sie die gesamten 150 ml getrunken?]

☐ Yes [Ja]

☐ No, if so, why not: ____________________________
[Nein, weshalb nicht:]

How much was left? ☐☐☐ mL
[Wieviel ist übrig geblieben?]

How much additional clear liquid did you consume?
[Wieviel an zusätzlicher klarer Flüssigkeit haben Sie getrunken?]

☐☐☐☐ mL From [Von] ☐☐:☐☐ to [Bis] ☐☐:☐☐
Plan: 250 mL per hour [250 ml pro Stunde]

Amount [Menge | Hour Stunde | Min. Min. | Hour Stunde | Min. Min.]

NORGINE	Date [Datum]<br><br>I__I__I I__I__I I__I__I__I__I<br><br>**PATIENT DOCUMENTATION FORM [STUDIEN DOKUMENTATION]**<br><br>**QUESTIONNAIRE [FRAGEBOGEN]**	NOR-01/2011 (PDR)<br><br>I__I__I - I__I__I__I<br>Patient Number [Patientennummer]

How do you judge the taste of the gut cleansing solution after the second 150 mL?
[Wie beurteilen Sie den Geschmack der zweiten 150 ml der Darmspüllösung?]

____

0 — Very bad [Sehr schlecht]
100 — Very good [Sehr gut]

(Please indicate your taste judgment with a single vertical line on the above scale.)
[(Bitte zutreffendes Maß mit einem einzigen senkrechten Strich auf der obigen Skala kennzeichnen.)]

Which of the following symptoms occurred during the intake of the second 150 mL of CitraFleet®?
[Welche der folgenden Beschwerden traten bei Ihnen während der Einnahme der zweiten 150 ml der Darmspüllösung auf?]

☐	☐	☐	☐	☐
None	Nausea	Vomiting	Abdominal Discomfort	Abdominal Pain
[Keine	Übelkeit	Erbrechen	Magen-beschwerden	Bauch-schmerzen]

How did you tolerate the second 150 mL of the CitraFleet® gut cleansing solution?
[Wie haben Sie die zweiten 150 ml der CitraFleet®-Darmspüllösung vertragen?]

☐	☐	☐	☐	☐
Very Good	Good	Acceptable	Bad	Very Bad
[Sehr gut	Gut	Zufriedenstellend	Schlecht	Sehr schlecht]

<table>
<tr><td rowspan="2">NORGINE</td><td>Date [Datum]

I__I__I I__I__I I__I__I__I__I

PATIENT DOCUMENTATION FORM
[STUDIEN DOKUMENTATION]

QUESTIONNAIRE
[FRAGEBOGEN]</td><td>NOR-01/2011 (PDR)

I__I__I - I__I__I__I
Patient Number
[Patientennummer]</td></tr>
</table>

Overall ratings at the end of the gut preparation

[Zusammenfassende Beurteilung am Ende der Darmvorbereitung]

Did you experience problems whilst drinking gut cleansing solution?
[Hatten Sie Probleme beim Trinken der vorgesehenen Menge der Darmspüllösung?]

☐ None [Keine] ☐ Some [Einige] ☐ Many [Viele]

How did you tolerate the intake of CitraFleet®?
[Wie haben Sie die Darmvorbereitung mit CitraFleet® vertragen?]

____

0	100
Extremely poorly tolerated<br>[Überhaupt nicht vertragen]	Perfectly well tolerated<br>[Sehr gut vertragen]

(Please indicate your taste judgment with a single vertical line on the above scale.)
[(Bitte zutreffendes Maß mit einem einzigen senkrechten Strich auf der obigen Skala kennzeichnen.)]

How did you find CitraFleet® as a gut cleansing solution?
[Waren Sie mit der Darmspüllösung zufrieden?]

____

0	100
Totally unacceptable<br>[Überhaupt nicht zufrieden]	Fully acceptable<br>[Sehr zufrieden]

(Please indicate your taste judgment with a single vertical line on the above scale.)
[(Bitte zutreffendes Maß mit einem einzigen senkrechten Strich auf der obigen Skala kennzeichnen.)]

NORGINE	Date [Datum]<br><br>I__I__I I__I__I I__I__I__I__I<br><br>**PATIENT DOCUMENTATION FORM [STUDIEN DOKUMENTATION]**<br><br>**QUESTIONNAIRE [FRAGEBOGEN]**	NOR-01/2011 (PDR)<br><br>I__I__I - I__I__I__I<br>Patient Number<br>[Patientennummer]

Were you satisfied with the whole gut cleansing preparation?
[Waren Sie zufrieden mit der gesamten Darmvorbereitung?]

0 — 100
Totally dissatisfied [Überhaupt nicht zufrieden] — Very satisfied [Sehr zufrieden]

(Please indicate the judgment of gut cleansing procedure with a single vertical line on the above scale.)
[(Bitte zutreffendes Maß mit einem einzigen senkrechten Strich auf der obigen Skala kennzeichnen.)]

The taste of the gut cleansing solution is:
[Der Geschmack der Lösung ist:]

☐ Good [Gut] ☐ Okay [Zufriedenstellend] ☐ Bad [Schlecht]

Drinking the CitraFleet® solution, as explained in the instructions, was:
[Die Einnahme der Darmspüllösung gemäß der Anleitung war:]

☐ Very easy [Sehr einfach] ☐ Easy [Einfach] ☐ Quite difficult [Nicht so einfach] ☐ Very difficult [Schwierig]

My tolerance of the CitraFleet® gut cleansing solution was:
Die Verträglichkeit der Darmvorbereitung mit der Darmspüllösung CitraFleet® war:

☐ Very good [Sehr gut] ☐ Good [Gut] ☐ Okay [Zufriedenstellend] ☐ Bad [Schlecht] ☐ Very bad [Sehr schlecht]

Thank you for your help!
[Vielen Dank für Ihre Mitarbeit!]

Norgine Ltd. Confidential Page 8 of 9
Version 1.0 Final 27 June 2011 (EN/Ger)

NORGINE	Date [Datum]<br><br>I__I__I I__I__I I__I__I__I__I<br><br>**PATIENT DOCUMENTATION FORM [STUDIEN DOKUMENTATION]**<br><br>**QUESTIONNAIRE [FRAGEBOGEN]**	NOR-01/2011 (PDR)<br><br>I__I__I - I__I__I__I<br>Patient Number<br>[Patientennummer]

Your colonoscopy is scheduled for:
[Ihre Koloskopie findet statt am:] ☐☐ ☐☐ ☐☐ **at [um]** ☐☐:☐☐
Day [Tag] Month [Monat] Year [Jahr] Hour [Std.] Min. [Min.]

Questionnaire reviewed by:
[Fragebogen überprüft:] ☐☐ ☐☐ ☐☐ ____________________
Day [Tag] Month [Monat] Year [Jahr] Investigator Signature [Unterschrift Prüfarzt]

**Have any clinically relevant Adverse Events been identified? [Sind im bisherigen Verlauf klinisch relevante unerwünschte Ereignisse aufgetreten?]**	☐	**Yes [Ja]**	☐	**No [Nein]**
**Has the CRF been updated with the identified clinically relevant Adverse Events? [Wurde die CRF-Dokumentation mit den identifizierten klinisch relevanten unerwünschten Ereignissen komplettiert?]**	☐	**Yes [Ja]**	☐	**No [Nein]**

Date
[Datum] ☐☐ ☐☐ ☐☐ ____________________
Day [Tag] Month [Monat] Year [Jahr] Investigator Signature [Unterschrift Prüfarzt]

20.4 SmPC for MOVIPREP®

20.4.1 English Version

SUMMARY OF PRODUCT CHARACTERISTICS

1. QUALITATIVE AND QUANTITATIVE COMPOSITION

MOVIPREP®, powder for oral solution in sachets

2. QUALITATIVE UND QUANTITATIVE ZUSAMMENSETZUNG

The ingredients of MOVIPREP® are contained in two separate sachets.

Sachet A contains the following active substances:

Macrogol 3350	100 g
Sodium sulphate anhydrous	7.500 g
Sodium chloride	2.691 g
Potassium chloride	1.015 g

Sachet B contains the following active substances:

Ascorbic acid	4.700g
Sodium ascorbate	5.900g

The concentration of electrolyte ions when both sachets are made up to one litre of solution is as follows:

Sodium	181.6 mmol/l (of which not more than 56.2mmol is absorbable)
Sulphate	52.8 mmol/l
Chloride	59.8 mmol/l
Potassium	14.2 mmol/l
Ascorbate	29.8 mmol/l

This product contains 0.233 g of aspartame per sachet A.

For a full list of excipients, see section 6.1.

3. PHARMACEUTICAL FORM

Powder for oral solution.

Free flowing white to yellow powder in Sachet A.

Free flowing white to light brown powder in Sachet B.

4. CLINICAL PARTICULARS

4.1 Therapeutic indications

For bowel cleansing prior to any clinical procedures requiring a clean bowel, e.g. bowel endoscopy or radiology.

4.2 Posology and method of administration

Adults and elderly: A course of treatment consists of two litres of MOVIPREP. It is strongly recommended that one litre of clear liquid, which may include, water, clear soup, fruit juice without pulp, soft drinks, tea and/or coffee without milk, is also taken during the course of treatment.

A litre of MOVIPREP consists of one 'Sachet A' and one 'Sachet B' dissolved together in one litre of water. This reconstituted solution should be drunk over a period of one to two hours. This should be repeated with a second litre of MOVIPREP.

This course of treatment can be taken:

either divided as one litre of MOVIPREP® in the evening before and one litre of MOVIPREP® in the early morning of the day of the clinical procedure,

or, in the evening preceding the clinical procedure.

There should be at least one hour between the end of intake of fluid (MOVIPREP® or clear liquid) and the start of colonoscopy.

No solid food should be taken from the start of the course of treatment until after the clinical procedure.

Children: Not recommended for the use in children below 18 year of age, as

MOVIPREP® has not been studied in the paediatric population.

4.3 Contraindications

Do not use in patients with known or suspected:

- gastrointestinal obstruction or perforation
- disorders of gastric emptying (e.g. gastroparesis)
- ileus
- phenylketonuria (due to presence of aspartame)
- glucose-6-phosphate dehydrogenase deficiency (due to presence of ascorbate)
- hypersensitivity to any of the ingredients
- toxic megacolon which complicates very severe inflammatory conditions of the intestinal tract including Crohn's disease and ulcerative colitis.

Do not use in unconscious patients.

4.4 Special warnings and precautions for use

Diarrhoea is an expected effect resulting from the use of MOVIPREP®.

MOVIPREP® should be administered with caution to fragile patients in poor health or patients with serious clinical impairment such as:

- impaired gag reflex, or with a tendency to aspiration or regurgitation
- impaired consciousness
- severe renal insufficiency (creatinine clearance <30ml/min)
- cardiac impairment (NYHA grade III of IV)
- dehydration
- severe acute inflammatory disease

The presence of dehydration should be corrected before the use of MOVIPREP®.

Semi-conscious patients or patients prone to aspiration or regurgitation should be closely observed during administration, especially if this is via a nasogastric route.

If patients develop any symptoms indicating shifts of fluid/electrolytes (e.g. oedema, shortness of breath, increasing fatigue, cardiac failure), plasma electrolytes should be measured and any abnormality treated appropriately.

In debilitated fragile patients, patients with poor health, those with clinically significant

renal impairment and those at risk of electrolyte imbalance, the physician should consider performing a baseline and post-treatment electrolyte and renal function test.

If patients experience symptoms such as severe bloating, abdominal distention, abdominal pain or any other reaction which makes it difficult to continue the preparation, they may slow down or temporarily stop consuming MOVIPREP® and should consult their doctor.

4.5 Interaction with other medicinal products and other forms of interaction

Oral medication should not be taken within one hour of administration of MOVIPREP as it may be flushed from the gastro-intestinal tract and not absorbed. The therapeutic effect of drugs with a narrow therapeutic index or short half-life may be particularly affected.

4.6 Pregnancy and lactation

There are no data on the use of MOVIPREP during pregnancy or lactation and it should only be used if considered essential by the physician.

4.7 Effects on ability to drive and use machines

There is no known effect on the ability to drive and use machines.

4.8 Undesirable effects

Diarrhoea is an expected outcome of bowel preparation. Due to the nature of the intervention, undesirable effects occur in the majority of patients during the process of bowel preparation. Whilst these vary between preparations, nausea, vomiting, bloating, abdominal pain, anal irritation and sleep disturbance commonly occur in patients undergoing bowel preparation.

As with other macrogol containing products, allergic reactions including rash, urticaria, pruritus, angioedema and anaphylaxis are a possibility.

Data from clinical studies are available in a population of 825 patients treated with MOVIPREP in which undesirable effect data were actively elicited. Additionally, adverse events reported in postmarketing are included.

The frequency of adverse reactions to MOVIPREP® is defined using the following convention:

Very common ≥ 1/10 (≥ 10%)

Common ≥ 1/100, < 1/10 (≥ 1%, < 10%)

Uncommon ≥ 1/1000, < 1/100 (≥ 0.1%, <1%)

Rare ≥ 1/10,000, < 1/1,000 (≥ 0.01%, < 0.1%)

Very rare < 1/10,000 (< 0.01%)

Not known (cannot be estimated from the available data)

**Body System**	**Frequency**	**Adverse Drug Reaction**
Immune System Disorders	Not known	Anaphylaxis
Psychiatric Disorders	Common	Sleep disorder
Nervous System Disorders	Common	Dizziness, headache
	Not known	Convulsions associated with severe hyponatraemia
Cardiac Disorders	Not known	Transient increase in blood pressure
Gastrointestinal Disorders	Very common	Abdominal pain, nausea, abdominal distension, anal discomfort
	Common	Vomiting, dyspepsia
	Uncommon	Dysphagia
	Not known	Flatulence, retching
Hepatobiliary Disorders	Uncommon	Abnormal liver function tests
Skin and Subcutaneous Tissue Disorders	Not known	Pruritus, Urticaria, Rash
General Disorders and Administration Site Conditions	Very common	Malaise
	Common	Rigors, thirst, hunger
	Uncommon	Discomfort
Investigations	Not known	Electrolytes disturbances including blood bicarbonate decreased, hyper and hypocalcaemia, hypophosphataemia,

		hyponatraemia (occurs more commonly in patients taking concomitant medication affecting the kidneys such as ACE inhibitors and diuretics) and changes in the blood chloride levels.

4.9 Overdose

In case of gross accidental overdosage, where diarrhoea is severe, conservative measures are usually sufficient; generous amounts of fluid, especially fruit juices, should be given. In the rare event of overdose provoking severe metabolic derangement, intravenous rehydration may be used.

5. PHARMACOLOGICAL PROPERTIES

5.1 Pharmacodynamic properties

Pharmacotherapeutic group: A06A D

The oral administration of macrogol-based electrolyte solutions causes moderate diarrhoea and results in rapid emptying of the colon.

Macrogol 3350, sodium sulphate and high doses of ascorbic acid exert an osmotic action in the gut, which induce a laxative effect.

Macrogol 3350 increases the stool volume, which triggers colon motility via neuromuscular pathways.

The physiological consequence is a propulsive colonic transportation of the softened stools.

The electrolytes present in the formulation and the supplementary clear liquid intake are included to prevent clinically significant variations of sodium, potassium or water, and thus reduce dehydration risk.

5.2 Pharmacokinetic properties

Macrogol 3350, is unchanged along the gut. It is virtually unabsorbed from the gastro-intestinal tract. Any macrogol 3350 that is absorbed is excreted via the urine.

Ascorbic acid is absorbed mainly at the small intestine level by a mechanism of active transport, which is sodium dependant and saturable. There is an inverse relationship between the ingested dose and the percentage of the absorbed dose. For oral doses between 30 and 180mg an amount of about 70-85% of the dose is absorbed. Following oral intake of up to 12g ascorbic acid , it is known that only 2g is absorbed.

After high oral doses of ascorbic acid and when plasma concentrations exceed 14mg/litre, the absorbed ascorbic acid is mainly eliminated unchanged in the urine.

5.3 Preclinical safety data

Preclinical studies provide evidence that macrogol 3350, ascorbic acid and sodium sulphate have no significant systemic toxicity potential.

No studies have been carried out on the genotoxicity, carcinogenicity or toxic effect on reproduction with this product.

6. PHARMACEUTICAL PARTICULARS

6.1 List of excipients

Aspartame (E951)

Acesulfame Potassium (E950)

Lemon flavour containing maltodextrin, citral, lemon oil, xanthan gum, vitamin E.

6.2 Incompatibilities

Not applicable

6.3 Shelf life

Sachets	3 years
Reconstituted solution	24 hours

6.4 Special precautions for storage

Sachets: Store below 25°C. Store in the original package.

Reconstituted Solution: Store below 25°C. The solution may be refrigerated. Keep the solution covered.

6.5 Nature and contents of container

A paper / low density polyethylene / aluminium / low density polyethylene sachet containing 112g of powder ('sachet A') and a paper / low density polyethylene / aluminium / low density polyethylene sachet containing 11g of powder ('sachet B'). Both sachets are contained in a transparent bag. One pack of MOVIPREP contains a single treatment of two bags.

Pack sizes of 1, 10, 40, 80, 160 and 320 packs of a single treatment. Hospital packs of 40 single treatments. Not all pack sizes may be marketed.

6.6 Special precautions for disposal and other handling

Reconstitution of MOVIPREP® in water may take up to 5 minutes and is best performed by adding the powder to the mixing vessel first followed by the water. The patient should wait until all the powder has dissolved before drinking the solution.

After reconstitution in water MOVIPREP® consumption may begin immediately or if preferred it may be cooled before use.

7. MARKETING AUTHORISATION HOLDER

Norgine B.V.

Hogehilweg 7

1101 CA Amsterdam ZO

The Netherlands

8. MARKETING AUTHORISATION NUMBER(S)

PL: 20142/0005

9. DATE OF FIRST AUTHORISATION/RENEWAL OF THE AUTHORISATION

19/01/2006

10. DATE OF REVISION OF THE TEXT

July 2010

LEGAL CATEGORY

P

20.4.2 German Version

FACHINFORMATION

ZUSAMMENFASSUNG DER MERKMALE DES ARZNEIMITTELS

1. BEZEICHNUNG DES ARZNEIMITTELS

MOVIPREP®,

Pulver zur Herstellung einer Lösung zum Einnehmen

2. QUALITATIVE UND QUANTITATIVE ZUSAMMENSETZUNG

Die Inhaltsstoffe von MOVIPREP® sind in zwei verschiedenen Beuteln enthalten.

Beutel A enthält die folgenden Wirkstoffe:

Macrogol 3350	100 g
Natriumsulfat	7,500 g
Natriumchlorid	2,691 g
Kaliumchlorid	1,015 g

Beutel B enthält die folgenden Wirkstoffe:

Ascorbinsäure	4,700 g
Natriumascorbat	5,900 g

Eine 1-Liter-Lösung des Inhalts beider Beutel weist die folgenden Elektrolytionenkonzentrationen auf:

Natrium	181,6 mmol/l (davon nicht mehr als 56,2 mmol resorbierbar)
Sulfat	52,8 mmol/l
Chlorid	59,8 mmol/l
Kalium	14,2 mmol/l
Ascorbat	29,8 mmol/l

Dieses Produkt enthält pro Beutel A 0,233 g Aspartam.

Die vollständige Auflistung der sonstigen Bestandteile siehe Abschnitt 6.1.

3. DARREICHUNGSFORM

Pulver zur Herstellung einer Lösung zum Einnehmen.

Frei fließendes, weißes bis gelbes Pulver in Beutel A.

Frei fließendes, weißes bis hellbraunes Pulver in Beutel B.

4. KLINISCHE ANGABEN

4.1 Anwendungsgebiete

Zur Darmvorbereitung vor klinischen Maßnahmen, die einen sauberen Darm erfordern, beispielsweise endoskopische oder radiologische Untersuchungen des Darms.

4.2 Dosierung, Art und Dauer Der Anwendung

Erwachsene und ältere Patienten:

Eine Darmvorbereitung besteht aus der Verabreichung von zwei Litern MOVIPREP®. Es wird nachdrücklich empfohlen, während der Darmvorbereitung zusätzlich einen Liter klare Flüssigkeit, beispielsweise Wasser, klare Suppe, Fruchtsaft (ohne Fruchtfleisch), Softdrinks oder Tee und/oder Kaffee (ohne Milch) zu sich zu nehmen.

Ein Liter MOVIPREP® enthält den in einem Liter Wasser aufgelösten Inhalt von je einem Beutel A und Beutel B. Diese gebrauchsfertige Lösung sollte der Patient innerhalb von einer bis zwei Stunden trinken. Dieser Vorgang wird mit einem zweiten Liter MOVIPREP® wiederholt.

Die Darmvorbereitung kann wie folgt ablaufen:

- entweder Einnahme von einem Liter MOVIPREP® am Vorabend und einem weiteren Liter MOVIPREP® am frühen Morgen des Tages, an dem die klinische Maßnahme erfolgen soll,

oder

- Einnahme von zwei Litern MOVIPREP® am Vorabend der klinischen Maßnahme.

Mit der Koloskopie sollte frühestens eine Stunde nach Beenden der Flüssigkeits-Einnahme (MOVIPREP® oder klare Flüssigkeit) begonnen werden.

Ab dem Beginn der MOVIPREP®-Einnahme bis zum Ende der klinischen Maßnahme darf der Patient keine feste Nahrung zu sich nehmen.

Kinder:

Die Anwendung bei Kindern unter 18 Jahren wird nicht empfohlen, da keine Studien mit MOVIPREP® bei Kindern durchgeführt wurden.

4.3 Gegenanzeigen

Nicht anwenden bei Patienten, bei denen Folgendes bekannt ist oder vermutet wird:

- Gastrointestinale Obstruktion oder Perforation
- Störungen der Magenentleerung (z.B. Magenatonie)
- Ileus
- Phenylketonurie (MOVIPREP® enthält Aspartam)
- Glukose-6-Phosphatdehydrogenase-Mangel (MOVIPREP® enthält Ascorbat)
- Überempfindlichkeit gegen einen der Bestandteile
- Toxisches Megakolon als Komplikation schwerer entzündlicher Darmerkrankungen wie z.B. Morbus Crohn und Colitis ulcerosa.

MOVIPREP® darf nicht bei bewusstlosen Patienten angewendet werden.

4.4 Besondere Warnhinweise und Vorsichtsmaßnahmen für die Anwendung

Durchfall ist eine erwartete Wirkung bei der Anwendung von MOVIPREP®.

MOVIPREP® sollte bei anfälligen Patienten mit schlechtem Gesundheitszustand oder schwerwiegenden Gesundheitsbeeinträchtigungen wie z.B.:

- Eingeschränktem Würgereflex oder Aspirations- oder Regurgitationstendenz
- Getrübtem Bewusstseinszustand
- Schwerer Niereninsuffizienz (Kreatinin-Clearance < 30 ml/min)
- Herzinsuffizienz (NYHA-Klasse III oder IV)
- Dehydratation
- Schwerer akuter entzündlicher Darmerkrankung

nur mit Vorsicht angewendet werden.

Vor dem Einsatz von MOVIPREP® sollte eine eventuell vorhandene Dehydratation korrigiert werden.

Patienten, die nicht bei vollem Bewusstsein sind, und Patienten, bei denen es zu einer

Aspiration oder Regurgitation kommen könnte, sollten während der Anwendung engmaschig überwacht werden, insbesondere bei Verabreichung über eine nasogastrale Sonde.

Falls Anzeichen von Flüssigkeits- und Elektrolytverschiebungen auftreten (z.B. Ödeme, Atemnot, zunehmende Müdigkeit, Herzinsuffizienz), sollten die Elektrolyt-Plasmakonzentrationen bestimmt und eventuelle Abweichungen adäquat behandelt werden.

Bei geschwächten und gebrechlichen Patienten, Patienten mit einem schlechten Gesundheitszustand, mit klinisch signifikanter Niereninsuffizienz und einem Risiko für Elektrolytstörungen, sollte der Arzt vor und nach der Behandlung eine Überprüfung der Elektrolytwerte und die Durchführung eines Nierenfunktionstests in Betracht ziehen.

Falls Symptome wie schwerer Meteorismus, Blähungen, Bauchschmerzen oder andere Reaktionen auftreten, die das Fortsetzen der Darmvorbereitung erschweren, sollte der Patient das Trinken von MOVIPREP® verlangsamen oder vorübergehend unterbrechen und den behandelnden Arzt konsultieren.

4.5 Wechselwirkungen mit anderen Arzneimitteln und sonstige Wechselwirkungen

Oral einzunehmende Medikamente sollten innerhalb einer Stunde nach Anwendung von MOVIPREP® nicht eingenommen werden, weil diese sonst aus dem Verdauungstrakt herausgespült und daher nicht resorbiert werden könnten. Insbesondere die Wirkung von Arzneimitteln mit geringer therapeutischer Breite oder kurzer Halbwertszeit kann davon betroffen sein.

4.6 Schwangerschaft und Stillzeit

Es liegen keine Daten zur Anwendung von MOVIPREP® während der Schwangerschaft oder Stillzeit vor. MOVIPREP® sollte daher nur dann angewendet werden, wenn es der Arzt für unbedingt erforderlich hält.

4.7 Auswirkungen auf die Verkehrstüchtigkeit und die Fähigkeit zum Bedienen von Maschinen

Auswirkungen auf die Verkehrstüchtigkeit und die Fähigkeit zum Bedienen von Maschinen sind nicht bekannt.

4.8 Nebenwirkungen

Durchfall ist ein erwartetes Ergebnis der Darmvorbereitung. Wegen der Art der Intervention treten bei den meisten Patienten während der Darmvorbereitung unerwünschte Reaktionen auf. Diese sind im Einzelfall unterschiedlich, häufig kommt

es während der Darmvorbereitung bei den Patienten zu Übelkeit, Erbrechen, Blähungen, Bauchschmerzen, analen Irritationen und Schlafstörungen.

Wie bei anderen Macrogol enthaltenden Produkten ist die Möglichkeit allergischer Reaktionen wie Hautausschläge, Urticaria, Puritus, Angioödeme und Anaphylaxie gegeben.

Daten aus klinischen Studien liegen für insgesamt 825 mit MOVIPREP® behandelte Patienten vor, bei denen aktiv Daten zu unerwünschten Wirkungen erhoben wurden. Zusätzlich sind Nebenwirkungen einbezogen, von denen nach Markteinführung berichtet wurde.

Bei der Bewertung von Nebenwirkungen werden folgende Häufigkeitsangaben zugrunde gelegt:

Sehr häufig (>1/10)

Häufig (>1/100 bis <1/10)

Gelegentlich (>1/1.000 bis <1/100)

Selten (>1/10.000 bis <1/1.000)

Sehr selten (<1/10.000)

nicht bekannt (Häufigkeit auf Grundlage der verfügbaren Daten nicht abschätzbar)

**Organklasse**	**Häufigkeit**	**Nebenwirkungen**
Erkrankungen des Immunsystems	Nicht bekannt	Anaphylaxie
Psychiatrische Erkrankungen	Häufig	Schlafstörungen
Erkrankungen des Nervensystems	Häufig	Schwindel, Kopfschmerzen
	Nicht bekannt	Krampfanfälle im Rahmen einer ausgeprägten Hyponatriämie
Herzerkrankungen	Nicht bekannt	vorübergehender Anstieg des Blutdrucks

Erkrankungen des Gastrointestinaltrakts	Sehr häufig	Bauchschmerzen, Übelkeit, Blähungen, Analreizungen
	Häufig	Erbrechen, Dyspepsie
	Gelegentlich	Dysphagie
	Nicht bekannt	Aufgeblähtheit, Würgreflex
Leber- und Gallenerkrankungen	Gelegentlich	Leberfunktionstests anormal
Erkankungen der Haut und des Unterhautzellgewebes	Nicht bekannt	Pruritus, Nesselsucht, Hautausschlag
Allgemeine Erkrankungen und Beschwerden am Verabreichungsort	Sehr häufig	Unwohlsein
	Häufig	Rigor, Durst, Hunger
	Nicht bekannt	Unbehagen
Untersuchungen	Nicht bekannt	Elektrolytverschiebungen, einschließlich Bikarbonatkonzentration im Blut vermindert, Hyper- und Hypocalciämie, Phosphatkonzentration im Blut vermindert, Hyponatriämie (tritt häufiger bei Patienten auf, die gleichzeitig Medikamente einnehmen, die einen Einfluss auf die Niere haben, wie beispielsweise ACE-Inhibitoren und Diuretika) sowie Änderungen der Chloridkonzentration im Blut

4.9 Überdosierung

Bei schwerer Diarrhoe infolge massiver versehentlicher Überdosierung sind in der Regel konservative Maßnahmen ausreichend; es sollten große Mengen Flüssigkeit, insbesondere Fruchtsäfte, verabreicht werden. Im selten vorkommenden Fall einer mit schweren Stoffwechselentgleisungen einhergehenden Überdosierung kann eine intravenöse Rehydratation erfolgen.

5. PHARMAKOLOGISCHE EIGENSCHAFTEN

5.1 Pharmakodynamische Eigenschaften

Pharmakotherapeutische Gruppe: A06A D.

Die orale Einnahme Macrogol-basierender Elektrolytlösungen verursacht einen moderaten Durchfall und führt zu einer schnellen Entleerung des Darms.

Macrogol 3350, Natriumsulfat und hohe Dosen von Ascorbinsäure haben im Darm einen osmotischen Effekt, der eine abführende Wirkung induziert.

Macrogol 3350 erhöht das Stuhlvolumen, was über neuromuskuläre Wege eine gesteigerte Motilität des Kolons auslöst.

Die physiologische Folge hieraus ist ein verbesserter propulsiver Transport von aufgeweichtem Stuhl.

Die in der Formulierung vorliegenden Elektrolyte und die zusätzliche Zufuhr klarer Flüssigkeit verhindern, dass es zu klinisch relevanten Veränderungen des Natrium-, Kalium- oder Wasserhaushalts kommt. Daher ist das Dehydratationsrisiko verringert.

5.2 Pharmakokinetische Eigenschaften

Macrogol 3350 passiert den Darm, ohne verändert zu werden. Es wird im Gastrointestinaltrakt praktisch nicht resorbiert. Resorbiertes Macrogol 3350 wird über den Urin ausgeschieden.

Die Resorption von Ascorbinsäure erfolgt hauptsächlich im Dünndarm über einen aktiven Transportmechanismus, der natriumabhängig und sättigbar ist. Die eingenommene Dosis ist der resorbierten Dosis umgekehrt proportional. Bei oraler Gabe von 30-180 mg werden circa 70-85% der verabreichten Dosis resorbiert. Nach oraler Gabe von bis zu 12 g Ascorbinsäure werden nachweislich nur 2 g resorbiert.

Beträgt nach hochdosierter Gabe von Ascorbinsäure deren Plamakonzentration mehr als 14 mg/Liter, wird die resorbierte Ascorbinsäure überwiegend unverändert über den Urin ausgeschieden.

5.3 Präklinische Daten zur Sicherheit

Die präklinischen Studien belegen, dass weder Macrogol 3350 noch Ascorbinsäure oder Natriumsulfat eine signifikante systemische Toxizität aufweisen.

Es wurden keine Studien zur Genotoxizität, Kanzerogenität oder Reproduktionstoxizität mit diesem Präparat durchgeführt.

6. PHARMAZEUTISCHE ANGABEN

6.1 Liste der sonstigen Bestandteile

Aspartam (E951)

Acesulfam-Kalium (E950)

Zitronenaroma, enthält: Maltodextrin, Citral, Zitronenöl, Limonenöl, Xanthangummi, Vitamin E.

6.2 Inkompatibilitäten

Nicht zutreffend.

6.3 Dauer der Haltbarkeit

Beutel	3 Jahre
Gebrauchsfertige Lösung	24 Stunden

6.4 Besondere Vorsichtsmaßnahmen für die Aufbewahrung

Beutel: Nicht über 25ºC lagern. In der Originalverpackung aufbewahren.

Zubereitete Lösung: Nicht über 25ºC lagern. Die Lösung kann im Kühlschrank aufbewahrt werden. Lösung abgedeckt halten.

6.5 Art und Inhalt des Behältnisses

Der aus Papier/Polyethylen niedriger Dichte/Aluminium/Polyethylen niedriger Dichte bestehende "Beutel A" enthält 112 g Pulver, der ebenfalls aus Papier/Polyethylen niedriger Dichte/Aluminium/Polyethylen niedriger Dichte bestehende "Beutel B" enthält 11 g Pulver. Diese beiden Beutel sind zusammen in einem transparenten Umbeutel abgepackt. Eine MOVIPREP®-Packung enthält die für eine Darmvorbereitung erforderlichen zwei Umbeutel.

Packungsgrößen von 1, 10, 40, 80,160 und 320 Packungen einer einzelnen Anwendung.

Klinikpackung mit 40 einzelnen Anwendungen.

6.6 Besondere Vorsichtsmaßnahmen für die Beseitigung und sonstige Hinweise zur Handhabung

Das Auflösen von MOVIPREP® in Wasser kann bis zu 5 Minuten dauern; zuerst wird das Pulver in das Zubereitungsgefäß gegeben und nachfolgend das Wasser hinzugefügt. Sobald sich das Pulver völlig aufgelöst hat, kann die Lösung vom Patienten getrunken werden.

Nach Auflösen in Wasser kann MOVIPREP® sofort eingenommen werden oder, falls gewünscht, kann die Lösung vor der Anwendung gekühlt werden.

7. INHABER DER ZULASSUNG

Norgine B.V.

Hogehilweg 7

1101 CA Amsterdam ZO

Niederlande

8. ZULASSUNGSNUMMER(N)

65776.00.00

9. DATUM DER ERTEILUNG DER ZULASSUNG / VERLÄNGERUNG DER ZULASSUNG

07.12.2006

10. STAND DER INFORMATION

03/2010

11. VERKAUFSABGRENZUNG

Apothekenpflichtig

20.5 SmPC of CitraFleet®

20.5.1 English version, translated from German version (see section 20.5.2)

1 NAME OF THE PHARMACEUTICAL

CitraFleet® powder in a packet to make a solution for oral consumption

2 QUALITATIVE AND QUANTITATIVE COMPOSITION

One packet (15.08 g) contains the following active ingredients:

Sodium picosulphate	10.0 mg
Light magnesium oxide	3.5 g
Citric acid, anhydrous	10.97 g

Every packet also contains 5 mmol (or 195 mg) potassium (see Section 4.4).

For a complete list of all other ingredients, see Section 6.1.

3 ADMINISTRATION FORM

CitraFleet® powder in a packet to make a solution for oral consumption.

White crystalline powder with lemon flavour.

4 CLINICAL INFORMATION

4.1 Areas of application

For intestinal cleansing before every diagnostic examination which can only be effectively carried out with a well-purged intestinal tract (e.g. a colonoscopy or x-ray exam).

4.2 Dosage, method and duration of administration

Method of administration: a low-fibre diet is recommended on the day before the examination. To prevent dehydration from the CitraFleet® treatment, it is recommended to drink approx. 250 ml of water or other clear liquid per hour for the duration of the intestinal purging.

Instructions for reconstitution: See Section 6.6.

Adults (including older patients) over 18 years:

The content of a packet reconstituted in water according to the instructions should be

consumed orally before 8.00 a.m. on the day before the examination. The second packet should be taken accordingly 6-8 hours later.

4.3 Contraindications

Hypersensitivity against the ingredients of this drug product, congestive heart failure, grave dehydration, hypomagnesaemia, retention of stomach content, gastrointestinal ulceration, toxic colitis, toxic mega-colon, ileus, nausea and vomiting, ascites, abdominal surgical emergency (e.g. acute appendicitis), as well as known gastrointestinal obstruction or perforation or the suspicion thereof.

May not be used by patients with rhabdomyolysis, since muscle relaxants elicit rhabdomyolysis and therefore can lead to exacerbation of this disorder.

May not be used by patients with active inflammatory intestinal disorders, such as Morbus Crohn or Colitis ulcerosa.

It can lead to an accumulation of magnesium in the plasma of patients with greatly impaired renal function. In such cases, switch to another drug product.

4.4 Warnings and precautions for application

CitraFleet® may not be used as a "normal" laxative.

For fragile or weakened older patients, in rare cases the administration of CitraFleet® can lead to serious and possibly fatal electrolyte disorders. Therefore a careful benefit-risk estimation must be made before beginning administration of CitraFleet® to a patient in this risk group.

Special caution is required for the prescription of CitraFleet® with regard to the possible presence of a known contraindication. Especially note the great significance of sufficient hydration (drink plenty of fluids!) and necessary caution with the high-risk patients cited below, being sure to determine electrolyte levels before and after treatment.

Particular caution is also advised for older and debilitated patients as well as for people with increased risk of hypokalaemia or hyponatraemia.

Administer CitraFleet® with particular caution to patients with known water and/or electrolyte balance disorders or patients undergoing treatment with drug products which could influence the water and/or electrolyte balance, such as diuretics, corticosteroids, or lithium (see Section 4.5).

Caution is also required for patients who have recently undergone surgery in the gastrointestinal area, as well as for patients with kidney insufficiency, light to medium serious dehydration, hypotension or cardiac disorders.

The duration of the intestinal purging should not exceed 24 hours, since a longer

preparation can increase the risk of disturbances in the body's water and electrolyte balance.

CitraFleet® can modify the resorption of drug products regularly ingested orally. Caution is therefore required here as well. For example, among epilepsy patients previously well adjusted with antiepileptics there are individual reports of the drug product triggering cerebral convulsions (see 4.5 and 4.8).

This drug product contains 5 mmol (195 mg) potassium in each packet. This must be considered for patients with impaired renal function or patients on a low-potassium diet.

4.5 Interactions with other pharmaceuticals and other interactions

As a laxative, CitraFleet® accelerates passage through the gastrointestinal tract. Therefore the resorption of other orally ingested drug products such as antiepileptics, contraceptives, antidiabetic agents, antibiotics, can be changed during treatment with this agent (see Section 4.4).

To prevent the forming of a complex with magnesium, antibiotics from the tetracycline and fluoroquinolone group as well as penicillamine are to be taken at least 2 hours before and at least 6 hours after the CitraFleet® dosage.

The effectiveness of CitraFleet® is reduced by muscle relaxants from the group of swelling agents.

Caution is required for patients who are already being treated with drug products which can accompany hyperaemia (such as diuretics or corticosteroids) or when there is any special risk of hyperaemia (such as cardiac glycosides). Caution is also required for administration of CitraFleet® by patients under treatment with non-steroidal antiphlogistics (NSAID) or drug products with known SIADH-inducing effects, such as tricyclic antidepressants, selective serotonin-resorption inhibitors, neuroleptics, and carbamazepin, since these substances can increase the risk of water retention and/or electrolyte disorders.

4.6 Pregnancy and nursing

There are no available clinical data for CitraFleet® on exposed pregnancies or reproduction toxicity. Since picosulphates are stimulating laxatives, the administration of CitraFleet® during pregnancy should be avoided when possible for safety reasons.

No data are available on the administration of CitraFleet® while nursing. Based on the pharmaceutical kinetic properties of the active ingredient, a treatment with CitraFleet® can be considered during nursing.

4.7 Effects on the ability to drive and use machines

CitraFleet® can cause tiredness or dizziness, probably as a result of the dehydration, whereby this can have only a small or moderate impact on the fitness to drive and

operate machines.

4.8 Adverse effects

In the scope of clinical trials, the most frequent adverse events occurred during administration of combinations of sodium picosulphate plus magnesium citrate as direct effects on the intestinal track (stomach pain and nausea), or the consequences of diarrhoea and dehydration (disturbed sleep, xerostomia, thirstiness, headache and tiredness).

Adverse drug reactions are presented according to a system of organ classes as per MedDRA and "Preferred Terms", whereby the frequency details are given according to the following convention: very frequently (≥ 1/10); frequently (≥ 1/100, < 1/10): occasionally (≥ 1/1000, < 1/100). The calculations for frequency are based on data from evaluations of clinical studies. "Frequency not known" is stated for adverse drug reactions not reported in the scope of these clinical trials.

Disorders of the immune system

Frequency not known: anaphylactoid reactions, hypersensitivity

Metabolic and nutritional disorders

Frequency not known: hyponatraemia

Psychiatric disorders

Frequently: insomnia

Disorders of the nervous system

Frequently: headache

Occasionally: dizziness

Frequency not known: epilepsy, Grand-mal-attack, convulsion, state of bewilderment

Vascular disorders

Occasionally: orthostatic hypotension

Disorders of the gastrointestinal tract

Very frequently: stomach pain

Frequently: xerostomia, nausea, bloated belly, anal complaints, proctalgia

Occasionally: vomiting, faecal incontinence

Frequency not known: diarrhoea*, flatulence

* Diarrhoea is the primary clinical effect of CitraFleet®

Disorders of the skin and the subcutaneous cellular tissue

Frequency not known: rashes (including erythematous and maculopapular rash), urticaria, pruritus, purpura

General disorders and complaints at the site of administration

Frequently: thirst, tiredness

Frequency not known: pain

Hyponatraemia with or without its associated convulsions has been reported (see Section 4.4). For epileptic patients, cerebral convulsions or Grand-mal seizures without simultaneously existing hyponatraemia have been reported (see 4.4 and 4.5).

4.9 Overdose

No case studies are available on overdose of CitraFleet® or similar combinations of sodium picosulphate and magnesium citrate. Based on the drug product's mechanism of action, however, it must be assumed that an overdose of CitraFleet® leads to strong diarrhoea with dehydration and electrolyte loss. The dehydration could also lead to orthostatic hypotension and dizziness. Dehydration and disorders in the electrolyte balance can be remedied by hydration and electrolyte substitution.

5 PHARMACOLOGICAL PROPERTIES

5.1 Pharmacodynamic properties

Pharmacotherapeutic group: sodium picosulphate, combinations, ATC Code: A06A B58

CitraFleet® contains sodium picosulphate as an active ingredient, a stimulating laxative that acts locally in the colon, and magnesium citrate, which is an osmotic laxative which draws liquid into the large intestine. Together these active ingredients cause a pronounced bowel movement and stimulation of peristalsis. The intestines are thoroughly emptied and purged before an x-ray examination, colonoscopy or surgery. The product is not intended for use as a "normal" laxative.

5.2 Pharmacokinetic properties

Both active substances are locally effective in the large intestine and are not reabsorbed in detectable quantities.

In patients with strongly impaired renal function, the drug product can lead to an accumulation of magnesium in the plasma.

5.3 Preclinical data on safety

In prenatal development studies of rats and rabbits, no teratogenic potential could be observed after oral administration of sodium picosulphate in dosages of up to 100 mg/kg/day; however at this dosage level, embryo toxic effects were found for both species. For rats during late pregnancy (foetal development) and during lactation, the administered daily dose of 10 mg/kg led to a decrease of body weight and overall survival rate of the descendants. The male and female fertility rates were not impaired

with oral doses of sodium picosulphate of up to 100 mg/kg.

6. PHARMACEUTIC INFORMATION

6.1 List of other components

Potassium bicarbonate

Saccharin sodium

Lemon flavouring (citric flavouring, maltodextrin, RRR-alpha-Tocopherol E 307).

6.2 Incompatibilities

Not applicable.

6.3 Durability

Unopened packet: 18 months.

Use immediately after reconstitution as solution.

6.4 Special precautionary measures for storage

Do not store above 25°C.

6.5 Type and content of the container

The powder is provided in packets with single doses of 15.08 g each. The packets are produced in folding packs with 2, 50, 100, 200, 500 and 1000 packets or 50 packets (clinic package). The packet consists of a laminate with a polyester facing, an aluminium intermediate layer and an inner polyethylene layer.

It is possible that not all packaging sizes have been placed on the market.

6.6 Special precautionary measures for disposal and other notices on handling

Instructions for reconstitution:

Reconstitute the contents of a packet in a cup of water (about 150 ml). It produces a turbid solution. Stir the mixture for 2-3 minutes and then drink.

If the solution becomes hot, wait until it is sufficiently cooled down.

7 HOLDER OF THE PERMIT

E. C. De Witt & Company Limited
Aegon House
Daresbury Park
Daresbury

Warrington
Cheshire
WA4 4HS
United Kingdom

Co-marketing with:

Eisai GmbH
Lyoner Str. 36
60528 Frankfurt
Tel: 069/665850
Fax: 069/6658525

8 APPROVAL NUMBER

68805.00.00

9 DATE OF ISSUE OF THE APPROVAL

03 March 2008

10 STATUS OF THE INFORMATION

08/2009

11 SALES RESTRICTIONS

Pharmacy-only

20.5.2 German version

1 BEZEICHNUNG DES ARZNEIMITTELS

CitraFleet® Pulver zur Herstellung einer Lösung zum Einnehmen in einem Beutel

2 QUALITATIVE UND QUANTITATIVE ZUSAMMENSETZUNG

Ein Beutel (15,08 g) enthält folgende Wirkstoffe:

Natriumpicosulfat 10,0 mg

Leichtes Magnesiumoxid 3,5 g

Citronensäure, wasserfrei 10,97 g

Jeder Beutel enthält außerdem 5mmol (bzw. 195mg) Kalium (siehe Abschnitt 4.4).

Die vollständige Auflistung der sonstigen Bestandteile siehe Abschnitt 6.1.

3 DARREICHUNGSFORM

Pulver zur Herstellung einer Lösung zum Einnehmen in einem Beutel.

Weißes kristallines Pulver mit Zitronengeschmack.

4 KLINISCHE ANGABEN

4.1 Anwendungsgebiete

Zur Darmreinigung vor jeder diagnostischen Untersuchung, die nur bei einem gut gereinigten Darm sinnvoll durchgeführt werden kann, z. B. eine Koloskopie oder Röntgenuntersuchung.

4.2 Dosierung, Art und Dauer der Anwendung

Art der Anwendung: zum Einnehmen

Am Tag vor der Untersuchung wird eine ballaststoffarme Kost empfohlen. Zur Verhinderung einer Dehydratation unter der Behandlung mit CitraFleet® wird für die Dauer der darmentleerenden Wirkung empfohlen, pro Stunde ca. 250ml Wasser oder einer sonstigen klaren Flüssigkeit zu trinken.

Anleitung zur Rekonstitution:

Siehe Abschnitt 6.6.

Erwachsene (einschließlich ältere Patienten) ab 18 Jahren:

Der nach Anleitung inWasser rekonstituierte Inhalt eines Beutels wird vor 8.00 Uhr morgens am Tag vor der Untersuchung getrunken. Der zweite Beutel entsprechend 6 – 8 Stunden später.

4.3 Gegenanzeigen

Überempfindlichkeit gegen einen der Bestandteile dieses Präparates, dekompensierte Herzinsuffizienz, schwere Dehydratation, Hypermagnesiämie, Retention von Mageninhalt, gastrointestinale Ulzerationen, toxische Kolitis, toxisches Megakolon, Ileus, Übelkeit und Erbrechen, Aszites, abdominalchirurgischer Notfall, z. B. akute Appendizitis, sowie bekannte gastrointestinale Obstruktion oder Perforation bzw. Verdacht darauf.

Darf nicht angewendet werden bei Patienten mit Rhabdomyolyse, da Laxantien eine Rhabdomyolyse hervorrufen und daher zu einer Exazerbation dieser Erkrankung führen können.

Darf nicht angewendet werden bei Patienten mit aktiver entzündlicher Darmerkrankung, wie z. B. Morbus Crohn oder Colitis ulcerosa.

Bei Patienten mit stark eingeschränkter Nierenfunktion kann es zu einer Anhäufung von Magnesium im Plasma kommen. In solchen Fällen ist auf ein anderes Präparat auszuweichen.

4.4 Besondere Warnhinweise und Vorsichtsmaßnahmen für die Anwendung

CitraFleet® darf nicht als „normales“ Abführmittel angewendet werden. Unter CitraFleet® könnte es bei gebrechlichen oder geschwächten älteren Patienten in seltenen Fällen zu schweren und möglicherweise tödlich verlaufenden Elektrolytstörungen kommen. Daher ist vor Beginn der Anwendung von CitraFleet® bei einem Patienten in dieser Risikogruppe eine sorgfältige Nutzen-Risiko-Abwägung vorzunehmen.

Besondere Vorsicht ist geboten bei der Verordnung von CitraFleet® im Hinblick auf das eventuelle Vorliegen bekannter Gegenanzeigen. Besonders zu achten ist dabei auch auf den hohen Stellenwert einer ausreichenden Hydratation sowie bei den (nachfolgend genannten) Risikopatienten außerdem auf die Durchführung von Elektrolytbestimmungen vor und nach der Behandlung.

Besondere Vorsicht ist unter Umständen geboten bei älteren und geschwächten Patienten sowie bei Personen mit erhöhtem Hypokalämie- oder Hyponatriämierisiko.

Eine Anwendung vonCitraFleet® bei Patienten mit bekannten Störungen des Wasserund/ oder Elektrolythaushaltes bzw. bei Patienten unter einer Behandlung mit Arzneimitteln, die den Wasser- und/oder Elektrolythaushalt beeinflussen könnten, wie z. B. Diuretika, Kortikosteroide oder Lithium, darf nur mit Vorsicht erfolgen (siehe

4.5).

Vorsicht ist auch geboten bei Patienten, bei denen erst vor kurzem ein chirurgischer Eingriff im Magen-Darm-Bereich durchgeführt wurde, ferner bei Patienten mit Niereninsuffizienz, leichter bis mittelschwerer Dehydratation, Hypotonie oder einer Herzerkrankung.

Die Dauer der Darmreinigung sollte 24 h nicht überschreiten, da sich bei einer längeren Vorbereitung das Risiko für Störungen im Wasser- und Elektrolythaushalt erhöhen kann.

CitraFleet® kann die Resorption regelmäßig oral eingenommener Arzneimittel verändern. Daher ist hier Vorsicht geboten. So liegen beispielsweise bei zuvor mit Antiepileptika gut eingestellten Epilepsiepatienten Einzelfallmeldungen über die Auslösung zerebraler Krampfanfälle vor (siehe 4.5 und 4.8).

Dieses Arzneimittel enthält pro Beutel 5mmol (entsprechend 195mg) Kalium. Dies ist bei Patienten mit eingeschränkter Nierenfunktion oder Patienten unter einer kaliumarmen Diät zu beachten.

4.5 Wechselwirkungen mit anderen Arzneimitteln und sonstige Wechselwirkungen

Als Abführmittel beschleunigt CitraFleet® die Magen-Darm-Passage. Daher kann die Resorption anderer oral eingenommener Arzneimittel (z. B. Antiepileptika, Kontrazeptiva, Antidiabetika, Antibiotika) während der Behandlungmit diesemMittel verändert sein (siehe 4.4).

Antibiotika aus der Gruppe der Tetrazykline und Fluorchinolone sowie Penicillamin sind zur Vermeidung einer Komplexbildung mit Magnesium mindestens 2 Stunden vor und wenigstens 6 Stunden nach der CitraFleet®- Dosis einzunehmen.

Die Wirksamkeit von CitraFleet® wird durch Laxantien aus der Gruppe der Quellmittel herabgesetzt.

Vorsicht ist geboten bei Patienten, die bereits mit Arzneimitteln behandelt werden, die mit Hypokaliämie einhergehen können (wie z. B. Diuretika oder Kortikosteroide) oder bei deren Anwendung eine Hypokaliämie ein besonderes Risiko darstellt (z. B. Herzglykoside). Vorsicht ist auch geboten bei der Anwendung von CitraFleet® bei Patienten, die mit nichtsteroidalen Antiphlogistika (NSAID) oder Arzneimitteln mit bekannter SIADH-induzierender Wirkung behandelt werden, wie z. B. trizyklische Antidepressiva, selektive Serotonin-Wiederaufnahmehemmer, Neuroleptika und Carbamazepin, da diese Substanzen das Risiko einer Wasserretention und/oder von Elektrolytstörungen erhöhen können.

4.6 Schwangerschaft und Stillzeit

Zu CitraFleet® liegen weder klinische Daten zu exponierten Schwangerschaften noch Daten zur Reproduktionstoxizität vor. Da es sich bei Picosulfat um ein stimulierendes

Abführmittel handelt, ist auf die Anwendung von CitraFleet® während der Schwangerschaft aus Sicherheitsgründen möglichst zu verzichten.

Zur Anwendung von CitraFleet® während der Stillzeit liegen keine Erfahrungen vor. Aufgrund der pharmakokinetischen Eigenschaften der Wirkstoffe kann in der Stillzeit allerdings eine Behandlung mit CitraFleet® in Betracht gezogen werden.

4.7 Auswirkungen auf die Verkehrstüchtigkeit und die Fähigkeit zum Bedienen von Maschinen

Wahrscheinlich infolge der Dehydratation kann CitraFleet® zu Müdigkeit oder Schwindel führen, wobei dies einen geringen oder mäßigen Einfluss auf die Verkehrstüchtigkeit und die Fähigkeit zum Bedienen von Maschinen haben kann.

4.8 Nebenwirkungen

Bei den häufigsten im Rahmen klinischer Studien unter der Kombination Natriumpicosulfat plus Magnesiumcitrat aufgetretenen unerwünschten Ereignissen handelte es sich um direkte Wirkungen auf den Darm (Bauchschmerzen und Übelkeit) bzw. um Folgen von Durchfall und Dehydratation (Schlafstörungen, Mundtrockenheit, Durstgefühl, Kopfschmerzen und Müdigkeit).

Nebenwirkungen sind nachfolgend dargestellt nach Systemorganklassen gemäß MedDRA und „Preferred Terms", wobei die Häufigkeitsangaben nach folgender Konvention erfolgen: sehr häufig (≥1/10); häufig (≥1/100, 51/10); gelegentlich (≥1/1000, 51/100). Die Berechnungen zur Häufigkeit basieren auf Daten aus Auswertungen von klinischen Studien. Bei im Rahmen dieser klinischen Prüfungen nicht gemeldeten Nebenwirkungen wird „Häufigkeit nicht bekannt" angegeben.

Erkrankungen des Immunsystems

Häufigkeit nicht bekannt: Anaphylaktoide Reaktion, Überempfindlichkeit

Stoffwechsel- und Ernährungsstörungen

Häufigkeit nicht bekannt: Hyponatriämie

Psychiatrische Erkrankungen

Häufig: Schlafstörung

Erkrankungen des Nervensystems

Häufig: Kopfschmerzen

Gelegentlich: Schwindel

Häufigkeit nicht bekannt: Epilepsie, Grandmal- Anfall, Konvulsionen, Verwirrtheitszustand

Gefäßerkrankungen

Gelegentlich: Orthostatische Hypotonie

Erkrankungen des Gastrointestinaltrakts

Sehr häufig: Bauchschmerzen

Häufig: Mundtrockenheit, Übelkeit, Blähbauch, Analbeschwerden, Proktalgie

Gelegentlich: Erbrechen, Stuhlinkontinenz

Häufigkeit nicht bekannt: Durchfall*, Flatulenz

* Bei Durchfall handelt es sich um die

primäre klinischeWirkung von CitraFleet®

Erkankungen der Haut und des Unterhautzellgewebes

Häufigkeit nicht bekannt: Hautausschläge (einschließlich erythematöser und makulopapulöser Rash), Urtikaria, Pruritus, Purpura

Allgemeine Erkrankungen und Beschwerden am Verabreichungsort

Häufig: Durstgefühl, Müdigkeit

Häufigkeit nicht bekannt: Schmerzen

Über Hyponatriämie mit oder ohne damit einhergehende Konvulsionen wurde berichtet (siehe 4.4). Bei Epileptikern wurde über zerebrale Krampfanfälle / Grand-mal-Anfälle ohne gleichzeitig bestehende Hyponatriämie berichtet (siehe 4.4 und 4.5).

4.9 Überdosierung

Zu Überdosierungen mit CitraFleet® oder ähnlichen Kombinationen aus Natriumpicosulfat undMagnesiumcitrat liegen keine Fallberichte vor. Aufgrund des Wirkmechanismus des Präparats ist allerdings davon auszugehen, dass es bei einer Überdosierung mit CitraFleet® zu starken Durchfällen mit Dehydratation und Elektrolytverlusten kommt. Die Dehydratation könnte auch zu orthostatischer Hypotonie und Schwindel führen. Eine Dehydratation und Störungen im Elektrolythaushalt sind ggf. durch Flüssigkeitszufuhr und Elektrolytsubstitution zu korrigieren.

5 PHARMAKOLOGISCHE EIGENSCHAFTEN

5.1 Pharmakodynamische Eigenschaften

Pharmakotherapeutische Gruppe:

Natriumpicosulfat, Kombinationen,

ATC-Code: A06A B58.

Als Wirkstoffe enthält CitraFleet® Natriumpicosulfat, ein lokal im Kolon wirkendes stimulierendes Abführmittel, undMagnesiumcitrat, das als osmotisch wirkendes

Abführmittel Flüssigkeit in den Dickdarm zieht. Zusammen bewirken diese Wirkstoffe einen ausgeprägten Darmentleerungseffekt und eine Anregung der Peristaltik. Dadurch wird der Darm vor einer Röntgenuntersuchung, Koloskopie oder OP gründlich entleert und gereinigt. Für den Gebrauch als „normales“ Abführmittel ist das Präparat nicht vorgesehen.

5.2 Pharmakokinetische Eigenschaften

Beide Wirkstoffe sind im Dickdarm lokal wirksam und werden nicht in nachweisbaren Mengen resorbiert.

Bei Patienten mit stark eingeschränkter Nierenfunktion kann es zu einer Anhäufung von Magnesium im Plasma kommen.

5.3 Präklinische Daten zur Sicherheit

In pränatalen Entwicklungsstudien an Ratten und Kaninchen konnte nach oraler Verabreichung von Natriumpicosulfat in Dosierungen von bis zu 100mg/kg/Tag kein teratogenes Potential beobachtet werden; allerdings fanden sich bei dieser Dosisstufe bei beiden Spezies embryotoxische Wirkungen. An Ratten während der Spätträchtigkeit (fetale Entwicklung) und der Laktation verabreichte Tagesdosen von 10mg/kg führten zu einer Abnahme des Körpergewichts und der Überlebensrate bei den Nachkommen. Die männliche und weibliche Fertilität wurde von oralen Dosen von Natriumpicosulfat von bis zu 100mg/kg nicht beeinträchtigt.

6. PHARMAZEUTISCHE ANGABEN

6.1 Liste der sonstigen Bestandteile

Kaliumhydrogencarbonat

Saccharin-Natrium

Zitronenaroma (Zitronenaroma,Maltodextrin, RRR-alpha-Tocopherol E 307).

6.2 Inkompatibilitäten

Nicht zutreffend.

6.3 Dauer der Haltbarkeit

Ungeöffnete Beutel: 18Monate.

Unmittelbar nach erfolgter Rekonstitution anwenden.

6.4 Besondere Vorsichtsmaßnahmen Für Die Aufbewahrung

Nicht über 25 °C lagern.

6.5 Art und Inhalt des Behältnisses

Das Pulver wird in Beuteln mit Einzeldosen zu jeweils 15,08 g angeboten. Die Beutel werden in Faltschachteln zu 2, 50, 100, 200, 500 und 1000 Beuteln oder 50 Beuteln (Klinikpackung) konfektioniert. Die Beutel bestehen aus einem Laminat mit einer Polyesteraußenschicht, einer Aluminiumzwischenschicht und einer Polyethyleninnenschicht.

Es werden möglicherweise nicht alle Packungsgrößen in den Verkehr gebracht.

6.6 Besondere Vorsichtsmaßnahmen für die Beseitigung und sonstige Hinweise zur Handhabung

Anleitung zur Rekonstitution:

Den Inhalt eines Beutels in einer Tasse Wasser (circa 150ml) rekonstituieren. Es entsteht eine trübe Lösung. Diese ist 2 – 3 Minuten lang umzurühren und dann zu trinken Wenn die Lösung heiß wird, so lange warten, bis sie ausreichend abgekühlt ist.

7 INHABER DER ZULASSUNG

E. C. De Witt & Company Limited
Aegon House
Daresbury Park
Daresbury
Warrington
Cheshire
WA4 4HS
United Kingdom

Mitvertrieb durch:

Eisai GmbH
Lyoner Str. 36
60528 Frankfurt
Telefon: 069/665850
Telefax: 069/6658525

8 ZULASSUNGSNUMMER(N)

68805.00.00

9 DATUM DER ERTEILUNG DER ZULASSUNG / VERLÄNGERUNG DER ZULASSUNG

03 März 2008

10 STAND DER INFORMATION

08/2009

11 VERKAUFSABGRENZUNG

Apothekenpflichtig

20.6 Declaration of Helsinki:

WORLD MEDICAL ASSOCIATION DECLARATION OF HELSINKI
Ethical Principles for Medical Research Involving Human Subjects

Adopted by the 18th WMA General Assembly, Helsinki, Finland, June 1964, and amended by the:

29th WMA General Assembly, Tokyo, Japan, October 1975

35th WMA General Assembly, Venice, Italy, October 1983

41st WMA General Assembly, Hong Kong, September 1989

48th WMA General Assembly, Somerset West, Republic of South Africa, October 1996

52nd WMA General Assembly, Edinburgh, Scotland, October 2000

53rd WMA General Assembly, Washington 2002 (Note of Clarification on paragraph 29 added)

55th WMA General Assembly, Tokyo 2004 (Note of Clarification on Paragraph 30 added)

59th WMA General Assembly, Seoul, October 2008

A. Introduction

1. The World Medical Association (WMA) has developed the Declaration of Helsinki as a statement of ethical principles for medical research involving human subjects, including research on identifiable human material and data.

The Declaration is intended to be read as a whole and each of its constituent paragraphs should not be applied without consideration of all other relevant paragraphs.

2. Although the Declaration is addressed primarily to physicians, the WMA encourages other participants in medical research involving human subjects to adopt these principles.

3. It is the duty of the physician to promote and safeguard the health of patients, including those who are involved in medical research. The physician's knowledge and conscience are dedicated to the fulfilment of this duty.

4. The Declaration of Geneva of the WMA binds the physician with the words, “The health of my patient will be my first consideration,” and the International Code of

Medical Ethics declares that, “A physician shall act in the patient's best interest when providing medical care”.

5. Medical progress is based on research that ultimately must include studies involving human subjects. Populations that are underrepresented in medical research should be provided appropriate access to participation in research.

6. In medical research involving human subjects, the well-being of the individual research subject must take precedence over all other interests.

7. The primary purpose of medical research involving human subjects is to understand the causes, development and effects of diseases and improve preventive, diagnostic and therapeutic interventions (methods, procedures and treatments). Even the best current interventions must be evaluated continually through research for their safety, effectiveness, efficiency, accessibility and quality.

8. In medical practice and in medical research, most interventions involve risks and burdens.

9. Medical research is subject to ethical standards that promote respect for all human subjects and protect their health and rights. Some research populations are particularly vulnerable and need special protection. These include those who cannot give or refuse consent for themselves and those who may be vulnerable to coercion or undue influence.

10. Physicians should consider the ethical, legal and regulatory norms and standards for research involving human subjects in their own countries as well as applicable international norms and standards. No national or international ethical, legal or regulatory requirement should reduce or eliminate any of the protections for research subjects set forth in this Declaration.

B. Principles for all Medical Research

11. It is the duty of physicians who participate in medical research to protect the life, health, dignity, integrity, right to self-determination, privacy, and confidentiality of personal information of research subjects.

12. Medical research involving human subjects must conform to generally accepted scientific principles, be based on a thorough knowledge of the scientific literature, other relevant sources of information, and adequate laboratory and, as appropriate, animal experimentation. The welfare of animals used for research must be respected.

13. Appropriate caution must be exercised in the conduct of medical research that may harm the environment.

14. The design and performance of each research study involving human subjects must be clearly described in a research protocol. The protocol should contain a statement of the ethical considerations involved and should indicate how the principles in this Declaration have been addressed. The protocol should include information regarding funding, sponsors, institutional affiliations, other potential conflicts of interest, incentives for subjects and provisions for treating and/or compensating subjects who are harmed as a consequence of participation in the research study. The protocol should describe arrangements for post-study access by study subjects to interventions identified as beneficial in the study or access to other appropriate care or benefits.

15. The research protocol must be submitted for consideration, comment, guidance and approval to a research ethics committee before the study begins. This committee must be independent of the researcher, the sponsor and any other undue influence. It must take into consideration the laws and regulations of the country or countries in which the research is to be performed as well as applicable international norms and standards but these must not be allowed to reduce or eliminate any of the protections for research subjects set forth in this Declaration. The committee must have the right to monitor ongoing studies. The researcher must provide monitoring information to the committee, especially information about any serious adverse events. No change to the protocol may be made without consideration and approval by the committee.

16. Medical research involving human subjects must be conducted only by individuals with the appropriate scientific training and qualifications. Research on patients or healthy volunteers requires the supervision of a competent and appropriately qualified physician or other health care professional. The responsibility for the protection of research subjects must always rest with the physician or other health care professional and never the research subjects, even though they have given consent.

17. Medical research involving a disadvantaged or vulnerable population or community is only justified if the research is responsive to the health needs and priorities of this population or community and if there is a reasonable likelihood that this population or community stands to benefit from the results of the research.

18. Every medical research study involving human subjects must be preceded by careful assessment of predictable risks and burdens to the individuals and communities involved in the research in comparison with foreseeable benefits to them and to other individuals or communities affected by the condition under investigation.

19. Every clinical trial must be registered in a publicly accessible database before recruitment of the first subject.

20. Physicians may not participate in a research study involving human subjects unless they are confident that the risks involved have been adequately assessed and can be satisfactorily managed. Physicians must immediately stop a study when the risks are found to outweigh the potential benefits or when there is conclusive proof of positive and beneficial results.

21. Medical research involving human subjects may only be conducted if the importance of the objective outweighs the inherent risks and burdens to the research subjects.

22. Participation by competent individuals as subjects in medical research must be voluntary. Although it may be appropriate to consult family members or community leaders, no competent individual may be enrolled in a research study unless he or she freely agrees.

23. Every precaution must be taken to protect the privacy of research subjects and the confidentiality of their personal information and to minimize the impact of the study on their physical, mental and social integrity.

24. In medical research involving competent human subjects, each potential subject must be adequately informed of the aims, methods, sources of funding, any possible conflicts of interest, institutional affiliations of the researcher, the anticipated benefits and potential risks of the study and the discomfort it may entail, and any other relevant aspects of the study. The potential subject must be informed of the right to refuse to participate in the study or to withdraw consent to participate at any time without reprisal. Special attention should be given to the specific information needs of individual potential subjects as well as to the methods used to deliver the information. After ensuring that the potential subject has understood the information, the physician or another appropriately qualified individual must then seek the potential subject's freely-given informed consent, preferably in writing. If the consent cannot be expressed in writing, the non-written consent must be formally documented and witnessed.

25. For medical research using identifiable human material or data, physicians must normally seek consent for the collection, analysis, storage and/or reuse. There may be situations where consent would be impossible or impractical to obtain for such research or would pose a threat to the validity of the research. In such situations the research may be done only after consideration and approval of a research ethics committee.

26. When seeking informed consent for participation in a research study the physician should be particularly cautious if the potential subject is in a dependent relationship with the physician or may consent under duress. In such situations the informed consent should be sought by an appropriately qualified individual who is completely independent of this relationship.

27. For a potential research subject who is incompetent, the physician must seek informed consent from the legally authorized representative. These individuals must not be included in a research study that has no likelihood of benefit for them unless it is intended to promote the health of the population represented by the potential subject, the research cannot instead be performed with competent persons, and the research entails only minimal risk and minimal burden.

28. When a potential research subject who is deemed incompetent is able to give assent to decisions about participation in research, the physician must seek that assent in

addition to the consent of the legally authorized representative. The potential subject's dissent should be respected.

29. Research involving subjects who are physically or mentally incapable of giving consent, for example, unconscious patients, may be done only if the physical or mental condition that prevents giving informed consent is a necessary characteristic of the research population. In such circumstances the physician should seek informed consent from the legally authorized representative. If no such representative is available and if the research cannot be delayed, the study may proceed without informed consent provided that the specific reasons for involving subjects with a condition that renders them unable to give informed consent have been stated in the research protocol and the study has been approved by a research ethics committee. Consent to remain in the research should be obtained as soon as possible from the subject or a legally authorized representative.

30. Authors, editors and publishers all have ethical obligations with regard to the publication of the results of research. Authors have a duty to make publicly available the results of their research on human subjects and are accountable for the completeness and accuracy of their reports. They should adhere to accepted guidelines for ethical reporting. Negative and inconclusive as well as positive results should be published or otherwise made publicly available. Sources of funding, institutional affiliations and conflicts of interest should be declared in the publication. Reports of research not in accordance with the principles of this Declaration should not be accepted for publication.

C. Additional Principles for Medical Research Combined with Medical Care

31. The physician may combine medical research with medical care only to the extent that the research is justified by its potential preventive, diagnostic or therapeutic value and if the physician has good reason to believe that participation in the research study will not adversely affect the health of the patients who serve as research subjects.

32. The benefits, risks, burdens and effectiveness of a new intervention must be tested against those of the best current proven intervention, except in the following circumstances:

• The use of placebo, or no treatment, is acceptable in studies where no current proven intervention exists; or

• Where for compelling and scientifically sound methodological reasons the use of placebo is necessary to determine the efficacy or safety of an intervention and the patients who receive placebo or no treatment will not be subject to any risk of serious or irreversible harm. Extreme care must be taken to avoid abuse of this option.

33. At the conclusion of the study, patients entered into the study are entitled to be informed about the outcome of the study and to share any benefits that result from it, for example, access to interventions identified as beneficial in the study or to other appropriate care or benefits.

34. The physician must fully inform the patient which aspects of the care are related to the research. The refusal of a patient to participate in a study or the patient's decision to withdraw from the study must never interfere with the patient-physician relationship.

35. In the treatment of a patient, where proven interventions do not exist or have been ineffective, the physician, after seeking expert advice, with informed consent from the patient or a legally authorized representative, may use an unproven intervention if in the physician's judgement it offers hope of saving life, re-establishing health or alleviating suffering. Where possible, this intervention should be made the object of research, designed to evaluate its safety and efficacy. In all cases, new information should be recorded and, where appropriate, made publicly available.
