# Supplementary material for: Impact of the Quality of Bowel Cleansing on the Efficacy of Colonic Cancer Screening: A Prospective, Randomized, Blinded Study
Source: PLoS One. 2015 May 7;10(5):e0126067. doi: 10.1371/journal.pone.0126067 (PMC4423835; doi:10.1371/journal.pone.0126067)
Supplement: S1 Protocol Amendment — (PDF) [file pone.0126067.s003.pdf]

NOR-01/2011 (PDR)

Protocol Amendment 1

A multi-centre, randomised, investigator-blinded study comparing the polyp detection rate of two different types of bowel preparation: a 2-litre solution (MOVIPREP®) versus a hyperosmotic and stimulant combined low volume bowel preparation (Sodium Picosulfate and Magnesium Citrate)

EudraCT No. 2011-002364-25

Amendment 1, Version 2.0, Date: 23 February 2012

Acts as an amendment to:
Protocol, Version 2.0, Date: 14 October 2011

Sponsor: Norgine Ltd.
Norgine House, Widewater Place, Moorhall Road
Harefield, Uxbridge, UB9 6NS.
United Kingdom

Sponsor's Representative: [redacted]

Sponsor Medical Representative: Hans Rudolf Kloess
Norgine GmbH
Marburg, Germany.
Tel.: +49 (0) 642 19 85 222

Coordinating Investigator: Prof. Dr. med. Wolfgang Fischbach
Klinikum Aschaffenburg, Innere Medizin
Am Hasenkopf 1, 63739 Aschaffenburg, Germany.
Tel.: +49 (0) 6021 32 3011

Contract Research Organisation: Pierrel Research Europe GmbH
Zeche Katharina 6, 45307 Essen, Germany.
Tel.: +49 (0) 201 89 90 0

Confidentiality Statement

The information contained within this document is confidential and is the property of Norgine Ltd. This material should only be used in connection with matters authorised by Norgine Ltd. The information should not be disclosed to others without prior written authorisation from Norgine's representative.

Table of Contents

1.0 AUTHORISATION 3
2.0 RATIONALE FOR CHANGE 4
3.0 DETAILS OF CHANGES 5

1.0 AUTHORISATION

Authorisation of the protocol and obligations: The undersigned confirm that the protocol and all amendments, the CRFs and the appendices contain the necessary information and guidelines for the conduct of this study. The study will be performed and recorded according to this protocol and its approved amendments and all legal obligations and agreements will be followed as laid out below.

We have read the attached amendment number 1 dated 23 February 2012 to the protocol "A multi-centre, randomised, investigator-blinded study comparing the polyp detection rate of two different types of bowel preparation: a 2-litre solution (MOVIPREP®) versus a hyperosmotic and stimulant combined low volume bowel preparation (Sodium Picosulfate and Magnesium Citrate)" dated 14 October 2011 (Version 2), and agree to abide by all provisions set forth therein. We agree to comply with the International Conference on Harmonisation Guideline for Good Clinical Practice (ICH-GCP), EU Clinical Trials Directive, national and local regulations and the Declaration of Helsinki.

Sponsor's Representative
[redacted](Therapy Area Director): Date:

.... 23 FEB 2012

Sponsor's Medical Representative
Hans Rudolf Kloess (Medical Director): Date:

.... 24. FEB 2012

Coordinating Investigator
Prof. Dr. med. Wolfgang Fischbach: Date:

.... 27.2.2012

Principal Investigator: Date:

....

Sub-Investigator: Date:

....

2.0 RATIONALE FOR CHANGE

This protocol amendment has been created to include changes to the following:

- Inclusion Criteria – This section has been restructured to clarify:
  - the age groups within the study and,
  - how the reasons for which colonoscopy are indicated is recorded.

- Conduct of Study; Colonoscopy Visit – This section has been amended to clarify the criteria for documenting the outcomes (i.e. adverse events) in association with colonoscopy.

These changes are considered non-substantial and will be incorporated in the protocol via this amendment, which has been agreed by the Sponsor and Coordinating Investigator.

Details of these changes are outlined in Section 3.0.

The original text undergoing changes are underlined.

The revised text is in bold.

3.0 DETAILS OF CHANGES

Original text: Section 1, Synopsis; Study Population; Page 8:

1. Patient's written informed consent must be obtained prior to inclusion.
2. Male or female outpatients or inpatients aged 40 to 80 years <u>with an indication for complete colonoscopy.</u>
3. <u>Willing to undergo a colonoscopy for diagnostic or surveillance purposes</u>
4. <u>Patients with a known personal of familial risk of colon neoplasia, willing to undergo a screening colonoscopy</u>
5. Willing, able and competent to complete the entire procedure and to comply with study instructions.
6. Females of childbearing potential must employ an adequate method of contraception.

Revised text:

1. Patient's written informed consent must be obtained prior to inclusion.
2. Male or female outpatients or inpatients aged 40 to 80 years **willing to undergo a colonoscopy for:**
    1. **Diagnostic or surveillance purposes**
       or
    2. **Screening purposes where patient**
        a. **Has a known personal or familial risk of colon neoplasia,**
           or
        b. **Is aged 55-80, willing to undergo a screening colonoscopy**
3. Willing, able and competent to complete the entire procedure and to comply with study instructions.
4. Females of childbearing potential must employ an adequate method of contraception.

Original text: Section 8.1, Inclusion Criteria; Page 25:

1. Patient's written informed consent must be obtained prior to inclusion.

2. Male or female outpatients or inpatients aged 40 to 80 years <u>with an indication for complete colonoscopy.</u>
3. <u>Willing to undergo a colonoscopy for diagnostic or surveillance purposes</u>
4. <u>Patients with a known personal of familial risk of colon neoplasia, willing to undergo a screening colonoscopy</u>
5. Willing, able and competent to complete the entire procedure and to comply with study instructions.
6. Females of childbearing potential must employ an adequate method of contraception.

Revised text:

1. Patient's written informed consent must be obtained prior to inclusion.
2. Male or female outpatients or inpatients aged 40 to 80 years **willing to undergo a colonoscopy for:**
    1. **Diagnostic or surveillance purposes**
    or
    2. **Screening purposes where patient**
        a. **Has a known personal or familial risk of colon neoplasia,**
        or
        b. **Is aged 55-80, willing to undergo a screening colonoscopy**
3. Willing, able and competent to complete the entire procedure and to comply with study instructions.
4. Females of childbearing potential must employ an adequate method of contraception.

Original text: Section 12.1.2, Visit 2 – Colonoscopy Visit; Page 39:

On the day prior to colonoscopy, patients will start their bowel preparation. After ingestion of each dose of bowel preparation solution, patients will answer the corresponding questions in the PDF.

In the morning of the colonoscopy day, patients will come to the study site to perform the planned colonoscopy. The following assessments will be performed by the investigator prior to the colonoscopy:

- Review of concomitant medication,

- Review of AEs since the screening visit,
- Assessment of study medication compliance,
- PDF collection and review,
- Body weight, height, blood pressure, pulse rate, and body temperature,
- Drug accountability.

The patient will then undergo a full colonoscopy, to be completed by 2 p.m. The procedure will be conducted by an experienced gastroenterologist independent from the investigator responsible for dispense of study medication. This gastroenterologist will be blinded to the study medication.

The following assessments will be performed by the gastroenterologist responsible for colonoscopy:

- Documentation of polyps, adenomas and carcinomas,
- Colon cleansing quality using the Harefield Cleansing Scale©.

If a complete colonoscopy is not possible due to insufficient colon cleansing, a repeated bowel preparation is not allowed within the scope of this study. If a repetition of the colonoscopy is deemed medically necessary by the investigator, any data documented during such a procedure must not be used for the present study.

Standard care after the colonoscopy will be performed before the patient can leave the study site. Prior to discharge from the study site, the investigator will conduct a clinical assessment after the colonoscopy to ensure complete documentation of all side effects related to the gut preparation and colonoscopy procedure. Discharge from the colonoscopy unit without any outstanding medical concerns will represent the end of the study for each patient.

Revised text:

On the day prior to colonoscopy, patients will start their bowel preparation. After ingestion of each dose of bowel preparation solution, patients will answer the corresponding questions in the PDF.

In the morning of the colonoscopy day, patients will come to the study site to perform the planned colonoscopy. The following assessments will be performed by the investigator prior to the colonoscopy:

- Review of concomitant medication,

- Review of AEs since the screening visit,
- Assessment of study medication compliance,
- PDF collection and review,
- Body weight, height, blood pressure, pulse rate, and body temperature,
- Drug accountability.

The patient will then undergo a full colonoscopy, to be completed by 2 p.m. The procedure will be conducted by an experienced gastroenterologist independent from the investigator responsible for dispense of study medication. This gastroenterologist will be blinded to the study medication.

The following assessments will be performed by the gastroenterologist responsible for colonoscopy:

- Documentation of polyps, adenomas and carcinomas,
- Colon cleansing quality using the Harefield Cleansing Scale©.

If a complete colonoscopy is not possible due to insufficient colon cleansing, a repeated bowel preparation is not allowed within the scope of this study. If a repetition of the colonoscopy is deemed medically necessary by the investigator, any data documented during such a procedure must not be used for the present study.

Standard care after the colonoscopy will be performed before the patient can leave the study site. Prior to discharge from the study site, the investigator will conduct a clinical assessment after the colonoscopy to ensure complete documentation of all side effects related to the gut preparation and colonoscopy procedure. **Neoplasia discovered during the colonoscopy will not be recorded and analysed as AEs as the objective of the colonoscopy is to document polyps, adenomas and carcinomas.** Discharge from the colonoscopy unit without any outstanding medical concerns will represent the end of the study for each patient.
