# Supplementary material for: Impact of the Quality of Bowel Cleansing on the Efficacy of Colonic Cancer Screening: A Prospective, Randomized, Blinded Study
Source: PLoS One. 2015 May 7;10(5):e0126067. doi: 10.1371/journal.pone.0126067 (PMC4423835; doi:10.1371/journal.pone.0126067)
Supplement: S1 Table — (DOCX) [file pone.0126067.s004.docx]

**Supporting Information**

**S1 Table. Concomitant Diseases By System Organ Class and Preferred Term Occurring in ≥2% of All Patients (Safety Population, N=398)**

|  | **MOVIPREP^®^**  **(N = 201)** | | | **CitraFleet^®^**  **(N = 197)** | | | | **All**  **(N = 398)** | | |
| --- | --- | --- | --- | --- | --- | --- | --- | --- | --- | --- |
| **SOC/PT** | **n_pat_** | **%** | **n_epi_** | **n_pat_** | **%** | **n_epi_** | **n_pat_** | | **%** | **n_epi_** |
| All PT´s | 150 | 74.6% | 397 | 147 | 74.6% | 380 | 297 | | 74.6% | 777 |
| **Vascular disorders** | 87 | 43.3% | 88 | 79 | 40.1% | 81 | 166 | | 41.7% | 169 |
| Hypertension | 85 | 42.3% | 85 | 79 | 40.1% | 79 | 164 | | 41.2% | 164 |
| **Metabolism and nutrition disorders** | 68 | 33.8% | 91 | 62 | 31.5% | 87 | 130 | | 32.7% | 178 |
| Obesity | 28 | 13.9% | 28 | 24 | 12.2% | 24 | 52 | | 13.1% | 52 |
| Diabetes mellitus | 18 | 9.0% | 18 | 12 | 6.1% | 12 | 30 | | 7.5% | 30 |
| Hyperlipidaemia | 14 | 7.0% | 14 | 16 | 8.1% | 16 | 30 | | 7.5% | 30 |
| Hypercholesterolaemia | 13 | 6.5% | 13 | 13 | 6.6% | 13 | 26 | | 6.5% | 26 |
| Hyperuricaemia | 10 | 5.0% | 10 | 11 | 5.6% | 11 | 21 | | 5.3% | 21 |
| Type 2 diabetes mellitus | 3 | 1.5% | 3 | 5 | 2.5% | 5 | 8 | | 2.0% | 8 |
| **Endocrine disorders** | 43 | 21.4% | 43 | 34 | 17.3% | 35 | 77 | | 19.3% | 78 |
| Hypothyroidism | 34 | 16.9% | 34 | 26 | 13.2% | 26 | 60 | | 15.1% | 60 |
| Goitre | 8 | 4.0% | 8 | 7 | 3.6% | 7 | 15 | | 3.8% | 15 |
| **Gastrointestinal disorders** | 38 | 18.9% | 50 | 36 | 18.3% | 45 | 74 | | 18.6% | 95 |
| Gastrooesophageal reflux disease | 15 | 7.5% | 15 | 13 | 6.6% | 13 | 28 | | 7.0% | 28 |
| Gastritis | 7 | 3.5% | 7 | 7 | 3.6% | 7 | 14 | | 3.5% | 14 |
| **Cardiac disorders** | 16 | 8.0% | 17 | 16 | 8.1% | 19 | 32 | | 8.0% | 36 |
| Coronary artery disease | 10 | 5.0% | 10 | 6 | 3.0% | 6 | 16 | | 4.0% | 16 |
| Atrial fibrillation | 1 | 0.5% | 1 | 7 | 3.6% | 7 | 8 | | 2.0% | 8 |
| **Musculoskeletal and connective tissue disorders** | 16 | 8.0% | 17 | 14 | 7.1% | 14 | 30 | | 7.5% | 31 |
| **Psychiatric disorders** | 6 | 3.0% | 6 | 17 | 8.6% | 17 | 23 | | 5.8% | 23 |
| Depression | 5 | 2.5% | 5 | 10 | 5.1% | 10 | 15 | | 3.8% | 15 |
| **Surgical and medical procedures** | 8 | 4.0% | 8 | 15 | 7.6% | 15 | 23 | | 5.8% | 23 |
| Thyroidectomy | 3 | 1.5% | 3 | 6 | 3.0% | 6 | 9 | | 2.3% | 9 |
| **Nervous system disorders** | 14 | 7.0% | 16 | 3 | 1.5% | 3 | 17 | | 4.3% | 19 |
| **Reproductive system and breast disorders** | 11 | 5.5% | 11 | 6 | 3.0% | 6 | 17 | | 4.3% | 17 |
| Benign prostatic hyperplasia | 7 | 3.5% | 7 | 4 | 2.0% | 4 | 11 | | 2.8% | 11 |
| **Respiratory, thoracic and mediastinal disorders** | 9 | 4.5% | 9 | 8 | 4.1% | 8 | 17 | | 4.3% | 17 |
| Chronic obstructive pulmonary disease | 5 | 2.5% | 5 | 4 | 2.0% | 4 | 9 | | 2.3% | 9 |
| **Social circumstances** | 8 | 4.0% | 8 | 9 | 4.6% | 9 | 17 | | 4.3% | 17 |
| Postmenopause | 3 | 1.5% | 3 | 6 | 3.0% | 6 | 9 | | 2.3% | 9 |
| **Immune system disorders** | 6 | 3.0% | 11 | 6 | 3.0% | 10 | 12 | | 3.0% | 21 |
| **Neoplasms benign, malignant and unspecified (incl cysts and polyps**) | 4 | 2.0% | 4 | 6 | 3.0% | 7 | 10 | | 2.5% | 11 |

N = number of patients, npat = number of patients, % = percentage of patients based on population, nepi = number of episodes, SOC = system organ class, PT = preferred term

Note: Indications with the same low level term code are counted as often as they appear.
