# Supplementary material for: Impact of the Quality of Bowel Cleansing on the Efficacy of Colonic Cancer Screening: A Prospective, Randomized, Blinded Study
Source: PLoS One. 2015 May 7;10(5):e0126067. doi: 10.1371/journal.pone.0126067 (PMC4423835; doi:10.1371/journal.pone.0126067)
Supplement: S2 Table — (DOCX) [file pone.0126067.s005.docx]

**Supporting Information**

S2 Table. Colon Cleansing Quality Assessed by the Harefield Cleansing Scale (ITT Population)^[a]^

|  | | **PEG+Asc**  **N=201**  **n (%)** | **NaPic/MgCit**  **N=193**  **n (%)** | **Overall**  **N=394**  **n (%)** |
| --- | --- | --- | --- | --- |
| **Ascending Colon** | Grade 4 | 90 (44.8) | 6 (3.1) | 96 (24.4) |
|  | Grade 3 | 84 (41.8) | 34 (17.6) | 118 (29.9) |
|  | Grade 2 | 24 (11.9) | 75 (38.9) | 99 (25.1) |
|  | Grade 1 | 2 (1.0) | 59 (30.6) | 61 (15.5) |
|  | Grade 0 | 0 | 16 (8.3) | 16 (4.1) |
|  | Grade N/A | 0 | 2 (1.0) | 2 (0.5) |
|  | Missing | 1 (0.5) | 1 (0.5) | 2 (0.5) |
| **Transverse Colon Right Half** | Grade 4 | 106 (52.7) | 13 (6.7) | 119 (30.2) |
|  | Grade 3 | 77 (38.3) | 57 (29.5) | 134 (34.0) |
|  | Grade 2 | 17 (8.5) | 87 (45.1) | 104 (26.4) |
|  | Grade 1 | 0 | 24 (12.4) | 24 (6.1) |
|  | Grade 0 | 0 | 9 (4.7) | 9 (2.3) |
|  | Grade N/A | 0 | 2 (1.0) | 2 (0.5) |
|  | Missing | 1 (0.5) | 1 (0.5) | 2 (0.5) |
| **Transverse Colon Left Half** | Grade 4 | 114 (56.7) | 17 (8.8) | 131 (33.2) |
|  | Grade 3 | 70 (34.8) | 72 (37.3) | 142 (36.0) |
|  | Grade 2 | 15 (7.5) | 79 (40.9) | 94 (23.9) |
|  | Grade 1 | 1 (0.5) | 16 (8.3) | 17 (4.3) |
|  | Grade 0 | 0 | 7 (3.6) | 7 (1.8) |
|  | Grade N/A | 0 | 2 (1.0) | 2 (0.5) |
|  | Missing | 1 (0.5) | 0 | 1 (0.3) |
| **Descending Colon** | Grade 4 | 103 (51.2) | 16 (8.3) | 119 (30.2) |
|  | Grade 3 | 81 (40.3) | 80 (41.5) | 161 (40.9) |
|  | Grade 2 | 15 (7.5) | 71 (36.8) | 86 (21.8) |
|  | Grade 1 | 1 (0.5) | 17 (8.8) | 18 (4.6) |
|  | Grade 0 | 0 | 7 (3.6) | 7 (1.8) |
|  | Grade N/A | 0 | 1 (0.5) | 1 (0.3) |
|  | Missing | 1 (0.5) | 1 (0.5) | 2 (0.5) |
| **Sigmoid Colon** | Grade 4 | 102 (50.7) | 20 (10.4) | 122 (31.0) |
|  | Grade 3 | 78 (38.8) | 71 (36.8) | 149 (37.8) |
|  | Grade 2 | 18 (9.0) | 76 (39.4) | 94 (23.9) |
|  | Grade 1 | 1 (0.5) | 17 (8.8) | 18 (4.6) |
|  | Grade 0 | 1 (0.5) | 7 (3.6) | 8 (2.0) |
|  | Grade N/A | 0 | 2 (1.0) | 2 (0.5) |
|  | Missing | 1 (0.5) | 0 | 1 (0.3) |
| **Rectum** | Grade 4 | 113 (56.2) | 26 (13.5) | 139 (35.3) |
|  | Grade 3 | 71 (35.3) | 83 (43.0) | 154 (39.1) |
|  | Grade 2 | 15 (7.5) | 56 (29.0) | 71 (18.0) |
|  | Grade 1 | 1 (0.5) | 19 (9.8) | 20 (5.1) |
|  | Grade 0 | 0 | 8 (4.1) | 8 (2.0) |
|  | Grade N/A | 0 | 1 (0.5) | 1 (0.3) |
|  | Missing | 1 (0.5) | 0 | 1 (0.3) |

Grade 4 = Very good (colon empty and clean), Grade 3 = Good (presence of clear liquid in the bowel, but easily removed by suction), Grade 2 = Moderate (Brown liquid or semisolid remaining amounts of stool, fully removable by suction or displaceable, thus allowing a complete visualisation of the bowel mucosa), Grade 1 = Bad (Semisolid amounts of stool, only partially removable with a risk of incomplete visualisation of bowel mucosa), Grade 0 = Very bad (Semisolid or solid amounts of stool, consequently colonoscopy incomplete or needs to be terminated), N = total number of patients included in treatment group, n = number of patients, N/A = not applicable; NaPic/MgCit, sodium picosulfate/magnesium citrate; PEG+Asc, polyethylene glycol plus ascorbate.

[a] Assessed during withdrawal of the colonoscope.
