# Supplementary material for: Impact of the Quality of Bowel Cleansing on the Efficacy of Colonic Cancer Screening: A Prospective, Randomized, Blinded Study
Source: PLoS One. 2015 May 7;10(5):e0126067. doi: 10.1371/journal.pone.0126067 (PMC4423835; doi:10.1371/journal.pone.0126067)
Supplement: S3 Table — (DOCX) [file pone.0126067.s006.docx]

**Supporting Information**

**S3 Table. Incidence of TEAEs by System Organ Class and Preferred Term (Safety Population, N=398)**

|  | **MOVIPREP^®^**  **(N = 201)** | | | **CitraFleet^®^**  **(N = 197)** | | | **All**  **(N = 398)** | | |
| --- | --- | --- | --- | --- | --- | --- | --- | --- | --- |
| **SOC/PT** | **n_pat_** | **%** | **n_epi_** | **n_pat_** | **%** | **n_epi_** | **n_pat_** | **%** | **n_epi_** |
| All PT´s | 66 | 32.8% | 95 | 34 | 17.3% | 39 | 100 | 25.1% | 134 |
| **Gastrointestinal disorders** | 59 | 29.4% | 86 | 31 | 15.7% | 34 | 90 | 22.6% | 120 |
| Nausea | 29 | 14.4% | 34 | 15 | 7.6% | 15 | 44 | 11.1% | 49 |
| Abdominal pain | 22 | 10.9% | 23 | 8 | 4.1% | 8 | 30 | 7.5% | 31 |
| Abdominal pain upper | 12 | 6.0% | 16 | 3 | 1.5% | 3 | 15 | 3.8% | 19 |
| Vomiting | 7 | 3.5% | 7 | 2 | 1.0% | 2 | 9 | 2.3% | 9 |
| Haemorrhoids | 1 | 0.5% | 1 | 2 | 1.0% | 2 | 3 | 0.8% | 3 |
| Abdominal discomfort | 1 | 0.5% | 1 | 1 | 0.5% | 1 | 2 | 0.5% | 2 |
| Diverticulum | 1 | 0.5% | 1 | 0 | 0.0% | 0 | 1 | 0.3% | 1 |
| Dyspepsia | 1 | 0.5% | 1 | 0 | 0.0% | 0 | 1 | 0.3% | 1 |
| Flatulence | 1 | 0.5% | 1 | 0 | 0.0% | 0 | 1 | 0.3% | 1 |
| Lower gastrointestinal haemorrhage | 1 | 0.5% | 1 | 0 | 0.0% | 0 | 1 | 0.3% | 1 |
| Anal pruritus | 0 | 0.0% | 0 | 1 | 0.5% | 1 | 1 | 0.3% | 1 |
| Constipation | 0 | 0.0% | 0 | 1 | 0.5% | 1 | 1 | 0.3% | 1 |
| Diverticulum intestinal | 0 | 0.0% | 0 | 1 | 0.5% | 1 | 1 | 0.3% | 1 |
| **Injury, poisoning and procedural complications** | 3 | 1.5% | 3 | 1 | 0.5% | 1 | 4 | 1.0% | 4 |
| Post procedural complication | 1 | 0.5% | 1 | 1 | 0.5% | 1 | 2 | 0.5% | 2 |
| Post procedural haemorrhage | 2 | 1.0% | 2 | 0 | 0.0% | 0 | 2 | 0.5% | 2 |
| **Vascular disorders** | 2 | 1.0% | 2 | 1 | 0.5% | 1 | 3 | 0.8% | 3 |
| Hypertensive crisis | 1 | 0.5% | 1 | 1 | 0.5% | 1 | 2 | 0.5% | 2 |
| Hypertension | 1 | 0.5% | 1 | 0 | 0.0% | 0 | 1 | 0.3% | 1 |
| **Cardiac disorders** | 1 | 0.5% | 1 | 0 | 0.0% | 0 | 1 | 0.3% | 1 |
| Sinus tachycardia | 1 | 0.5% | 1 | 0 | 0.0% | 0 | 1 | 0.3% | 1 |
| **General disorders and administration site conditions** | 1 | 0.5% | 1 | 0 | 0.0% | 0 | 1 | 0.3% | 1 |
| Pyrexia | 1 | 0.5% | 1 | 0 | 0.0% | 0 | 1 | 0.3% | 1 |
| **Infections and infestations** | 1 | 0.5% | 1 | 0 | 0.0% | 0 | 1 | 0.3% | 1 |
| Herpes zoster | 1 | 0.5% | 1 | 0 | 0.0% | 0 | 1 | 0.3% | 1 |
| **Skin and subcutaneous tissue disorders** | 1 | 0.5% | 1 | 0 | 0.0% | 0 | 1 | 0.3% | 1 |
| Dermatitis allergic | 1 | 0.5% | 1 | 0 | 0.0% | 0 | 1 | 0.3% | 1 |
| **Nervous system disorders** | 0 | 0.0% | 0 | 3 | 1.5% | 3 | 3 | 0.8% | 3 |
| Headache | 0 | 0.0% | 0 | 3 | 1.5% | 3 | 3 | 0.8% | 3 |

N = number of patients; npat = number of patients; % = percentage of patients based on population; nepi = number of episodes; PT = preferred term; SOC = system organ class; TEAE = treatment-emergent adverse event
