# Supplementary material for: Impact of the Quality of Bowel Cleansing on the Efficacy of Colonic Cancer Screening: A Prospective, Randomized, Blinded Study
Source: PLoS One. 2015 May 7;10(5):e0126067. doi: 10.1371/journal.pone.0126067 (PMC4423835; doi:10.1371/journal.pone.0126067)
Supplement: S4 Table — (DOCX) [file pone.0126067.s007.docx]

**Supporting Information**

S4 Table. Patient Tolerability and Acceptability of PEG+Asc and NaPic/MgCit Bowel Preparations Assessed by Patient Completed Questionnaire (Safety Population)

|  | **PEG+Asc (N=201)**  **n (%)** | | **NaPic/MgCit (N=197)**  **n (%)** | |
| --- | --- | --- | --- | --- |
| **How did you tolerate the 1^st^/2^nd^ L/150 mL of the cleansing solution?** | | | | |
|  | **1^st^ Liter** | **2^nd^ Liter** | **1^st^ 150 mL** | **2^nd^ 150 mL** |
| Very good | 54 (26.9) | 49 (24.4) | 99 (50.3) | 92 (46.7) |
| Good | 103 (51.2) | 95 (47.3) | 86 (43.7) | 83 (42.1) |
| Acceptable | 35 (17.4) | 41 (20.4) | 7 (3.6) | 11 (5.6) |
| Bad | 5 (2.5) | 9 (4.5) | 0 | 6 (3.0) |
| Very bad | 0 | 1 (0.5) | 0 | 0 |
| Missing | 4 (2.0) | 6 (3.0) | 5 (2.5) | 5 (2.5) |
| **Did you experience problems during the drinking of the bowel cleansing solution?** | | | | |
| None | 128 (63.7) | | 190 (96.4) | |
| Some | 65 (32.3) | | 2 (1.0) | |
| Many | 4 (2.0) | | 0 | |
| Missing | 4 (2.0) | | 5 (2.5) | |
| **Tolerance of the PEG+Asc/NaPic/MgCit bowel cleansing solution was:** | | | | |
| Very good | 44 (21.9) | | 87 (44.2) | |
| Good | 114 (56.7) | | 90 (45.7) | |
| Acceptable | 32 (15.9) | | 14 (7.1) | |
| Bad | 7 (3.5) | | 1 (0.5) | |
| Very bad | 0 | | 0 | |
| Missing | 4 (2.0) | | 5 (2.5) | |
| **Which of the following symptoms occurred during the intake of the 1^st^/2^nd^ L/150 mL of PEG+Asc/NaPic/MgCit?** | | | | |
|  | **1^st^ Litre** | **2^nd^ Litre** | **1^st^ 150 mL** | **2^nd^ 150 mL** |
| None | 141 (70.1) | 148 (73.6) | 174 (88.3) | 172 (87.3) |
| Nausea | 23 (11.4) | 34 (16.9) | 10 (5.1) | 11 (5.6) |
| Vomiting | 3 (1.5) | 5 (2.5) | 0 | 3 (1.5) |
| Abdominal discomfort | 6 (3.0) | 2 (1.0) | 2 (1.0) | 3 (1.5) |
| Abdominal pain | 34 (16.9) | 14 (7.0) | 8 (4.1) | 7 (3.6) |
